# One-Shot Tagging During Wake and Cueing During Sleep With Spatiotemporal Patterns of Transcranial Electrical Stimulation Can Boost Long-Term Metamemory of Individual Episodes in Humans

**DOI:** 10.3389/fnins.2019.01416

**Published:** 2020-01-10

**Authors:** Praveen K. Pilly, Steven W. Skorheim, Ryan J. Hubbard, Nicholas A. Ketz, Shane M. Roach, Itamar Lerner, Aaron P. Jones, Bradley Robert, Natalie B. Bryant, Arno Hartholt, Teagan S. Mullins, Jaehoon Choe, Vincent P. Clark, Michael D. Howard

**Affiliations:** ^1^Center for Human-Machine Collaboration, Information and Systems Sciences Laboratory, HRL Laboratories, LLC, Malibu, CA, United States; ^2^Center of Molecular and Behavior Neuroscience, Rutgers University Newark, Newark, NJ, United States; ^3^Department of Psychology, The University of Texas at San Antonio, San Antonio, TX, United States; ^4^Psychology Clinical Neuroscience Center, Department of Psychology, University of New Mexico, Albuquerque, NM, United States; ^5^Institute for Creative Technologies, University of Southern California, Los Angeles, CA, United States

**Keywords:** memory consolidation, non-invasive stimulation, learning and memory, metamemory, targeted memory reactivation

## Abstract

Targeted memory reactivation (TMR) during slow-wave oscillations (SWOs) in sleep has been demonstrated with sensory cues to achieve about 5–12% improvement in post-nap memory performance on simple laboratory tasks. But prior work has not yet addressed the one-shot aspect of episodic memory acquisition, or dealt with the presence of interference from ambient environmental cues in real-world settings. Further, TMR with sensory cues may not be scalable to the multitude of experiences over one’s lifetime. We designed a novel non-invasive non-sensory paradigm that tags one-shot experiences of minute-long naturalistic episodes in immersive virtual reality (VR) with unique spatiotemporal amplitude-modulated patterns (STAMPs) of transcranial electrical stimulation (tES). In particular, we demonstrated that these STAMPs can be re-applied as brief pulses during SWOs in sleep to achieve about 10–20% improvement in the metamemory of targeted episodes compared to the control episodes at 48 hours after initial viewing. We found that STAMPs can not only facilitate but also impair metamemory for the targeted episodes based on an interaction between pre-sleep metamemory and the number of STAMP applications during sleep. Overnight metamemory improvements were mediated by spectral power increases following the offset of STAMPs in the slow-spindle band (8–12 Hz) for left temporal areas in the scalp electroencephalography (EEG) during sleep. These results prescribe an optimal strategy to leverage STAMPs for boosting metamemory and suggest that real-world episodic memories can be modulated in a targeted manner even with coarser, non-invasive spatiotemporal stimulation.

## Introduction

The ability to recall previously experienced events and to introspect about them are important aspects of our daily living. However, a mechanistic understanding of how memories of one-shot experiences of real-world episodes are formed, recalled, and monitored in the human brain is still lacking. Metamemory is an executive function that monitors and judges the ability to recall memories accurately (Nelson and Narens, 1990), such as when providing eyewitness testimony in a criminal case or deciding when study material has been sufficiently learned. One is said to have higher metamemory when recall accuracy is proportional to subjective confidence (i.e., more confident when correct and less confident when wrong). In other words, metamemory measures the ability to introspect and discriminate between correct and incorrect memory recalls, avoiding either over- or under-confidence (Galvin et al., 2003; Fleming and Lau, 2014). The neural mechanisms underlying memory monitoring and control have been suggested to work in concert with those involved in the encoding, consolidation, and recall of the memory content (Nelson and Narens, 1990).

Hippocampus is known to play an important role in the online rapid encoding of episodic memories for short-term storage, which subsequently drives offline consolidation for long-term storage in distributed neocortical areas (McClelland et al., 1995; Buzsáki, 1996). But it is also possible for neocortical activations during offline periods to trigger memory replays in the hippocampus (Ji and Wilson, 2007; Rothschild et al., 2017). Consistent with this latter view, there have been a number of demonstrations of offline targeted memory reactivation (TMR) in animals and humans using olfactory and auditory cues to modulate the ability to learn contexts and individual memories (e.g., Rasch et al., 2007; Rudoy et al., 2009; Antony et al., 2012; Bendor and Wilson, 2012). However, these studies have not yet addressed the one-shot aspect of episodic memory acquisition, or dealt with the presence of interference from ambient environmental cues in real-world settings. And a majority of these studies assessed memory performance over less than a day, with about 5–12% improvement in post-nap memory performance on simple laboratory tasks (e.g., Rudoy et al., 2009; Antony et al., 2012), and employed fixed-dose cueing during offline periods. Further, TMR with sensory cues may not be scalable to the multitude of experiences over one’s lifetime. Our study overcomes these limitations by investigating long-term behavioral and physiological effects of non-sensory transcranial electrical stimulation (tES) for TMR of naturalistic episodic memories with one-shot acquisition.

Prior work on non-sensory cueing showed that transcranial magnetic stimulation can reactivate the experience of a visual stimulus after repeated pairing with it (Liao et al., 2013). And transcranial alternating current stimulation (tACS) of prefrontal cortex (PFC) at a given frequency (60 or 90 Hz) during encoding can boost subsequent performance for old vs. new recognition of learned words when reapplied at the same frequency during either retrieval (Javadi et al., 2017) or slow-wave (SW) sleep (Crowley and Javadi, 2019). Employing intracranial recordings in awake non-human primates, we showed that transcranial direct current stimulation (tDCS) of PFC alters functional connectivity between brain areas in a frequency-specific manner (Krause et al., 2017). Recently, we showed that tACS can reliably entrain the spiking activity of single neurons in deep structures such as the hippocampus and basal ganglia in a spatially-localized and frequency-dependent manner (Krause et al., 2019b). Building on these prior results, we postulated that spatiotemporal amplitude-modulated patterns (STAMPs) of tES could alter the functional connectivity as well as the spike timing within the brain in unique ways and thereby be leveraged for TMR in more potent ways than sensory cues. In particular, we investigated whether STAMPs of tES could be used to tag specific naturalistic episodes during one-shot viewing in immersive virtual reality (VR) and subsequently cue them during sleep to boost their memory recall over 48 hours in a targeted manner. The overarching goal of this study was to assess if coarser, non-invasive stimulation is sufficient to effectively modulate complex episodic memories in humans.

## Materials and Methods

### STAMPs

Each STAMP is defined as an array of currents across 32 stimulation electrodes located in the 64-channel layout according to the international 10-10 system (see Supplementary Figure S1). A library of 256 spatial stimulation patterns was computed based on the criterion that the induced electric fields in the 3D brain volume of a realistic adult human head template (with a cortical mesh of 190,521 dimensions) are as mutually orthogonal as possible. Gradient descent optimization was used to minimize the norm of the cross-correlation function for electric fields across the library. The optimization procedure penalized both correlations and anti-correlations for the electric fields induced across solutions to accommodate both tDCS and tACS STAMPs with the solved spatial patterns as amplitudes. The total injected current was set to 2.5 mA, with maximum 1.5 mA and minimal 150 μA current at any electrode (to avoid impedance issues). Different initializations of the gradient descent search, in terms of the number of starting non-zero current electrodes, yielded STAMP solutions with different sparseness amounts. Solutions using more initial non-zero current electrodes led to STAMP sets with lower overall cross-correlation and lower currents across the montages. STAMPs used in the current study were solved based on initialization of 18 non-zero current electrodes. Of the 256 computed amplitude patterns, 14 were randomly chosen for use in the current study as tDCS and 40 Hz tACS STAMPs.

### Subjects

Subjects were 18–40 years of age, used English as a first language, completed high school, and had no history of head injury with loss of consciousness for longer than 5 min. They were right-handed according to the Edinburgh Handedness Inventory (Oldfield, 1971), had no history of neurological or psychiatric disorder, had no history of alcohol or drug abuse, were non-smoking, had no excessive alcohol or caffeine consumption, were not currently taking any medication significantly affecting the central nervous system, had no implanted metal, had no sensitivity or allergy to latex, had good or corrected hearing and vision, and reported no sleep disturbances. Women who were pregnant, or thought they might be, were also excluded.

A total of 30 healthy subjects completed the experiment. They were recruited using flyers placed around campus of the University of New Mexico and surrounding community, and received monetary compensation upon completion of the study. Of these, six subjects were excluded from the analyses due to either equipment failure during an “Active” night, or non-compliance in following task instructions. All subjects provided signed informed consent to participate in the study, which was approved by the Chesapeake Institutional Review Board. The remaining *N* = 24 subjects (15 female) had a mean age of 23.96 years with a standard deviation of 6.08 years.

### Behavioral Paradigm

We employed immersive VR to produce simulated realistic environments for the purpose of systematically investigating the modulation of human episodic memories with STAMPs. VR-derived results have greater predictive validity and relevance for real-world applications when compared to results from standard training and testing tools on a personal computer. In addition, on a more pragmatic level, rather than relying on costly physical mock-ups of functional environments, VR offers the option to produce and distribute identical standard simulation environments.

Subjects were able to freely look around a fully rendered 3D environment on the HTC Vive^®^ platform. Their virtual vantage point was situated on a balcony across from an apartment building set in a non-descript Middle Eastern town. Supplementary Movie S1, “Fire Response”, is an illustrative episode. The task was to actively surveil the inhabitants of the building and the passers-by so that they would be able to later recall the events. We designed 28 distinct memorable episodes in the VR, each about a minute long. The series of events in an episode generally centered around two main characters, often with one or two less involved characters. Ten declarative statements were composed for each episode to test the ability of the subjects to recall facts about the experienced events. Subjects were instructed to respond to salient events in each episode as they happened by orienting a reticle in the head-mounted display (HMD) toward those events and taking pictures with a virtual camera triggered by a hand-held controller.

In preparation for the experiment, the episodes and questions (i.e., declarative statements) were gradually improved using feedback from 12 pilot subjects (who are different from the subjects for the main experiment). They watched one episode at a time and immediately rated the difficulty of each of the 10 questions on a scale from 1 (easy) to 10 (difficult), and the overall memorability of the episode on a scale from 1 (least memorable) to 10 (most memorable). Average difficulty rating in the subjects’ responses tended to covary with the accuracy of their responses across the episodes. We employed an iterative process aimed at subjectively equalizing the overall memorability and question difficulty across the episodes. Each iteration involved getting responses from four pilot subjects, after which the less memorable episodes were altered to increase memorability by adding more salient events. Questions that were answered incorrectly and rated as difficult, across the subjects, were made easier, and those that were answered correctly and rated as easy were increased in difficulty. After three iterations, the 28 episodes were of similar memorability, and the questions were of similar difficulty (see Supplementary Note). Using data from one final cohort of four pilot subjects, the set of 28 finalized episodes was curated into four subgroups with the constraints that the average difficulty of the questions and the average memorability of the episodes in each subgroup were similar, and the themes of the events occurring in the episodes were roughly matched. Two groups of 14 episodes each (namely, A and B) were created by randomly choosing among these four subgroups. Episodes were given names but were not presented to the subjects and were used only for reference by the experimenters (see Supplementary Table S1).

**FIGURE 1:**
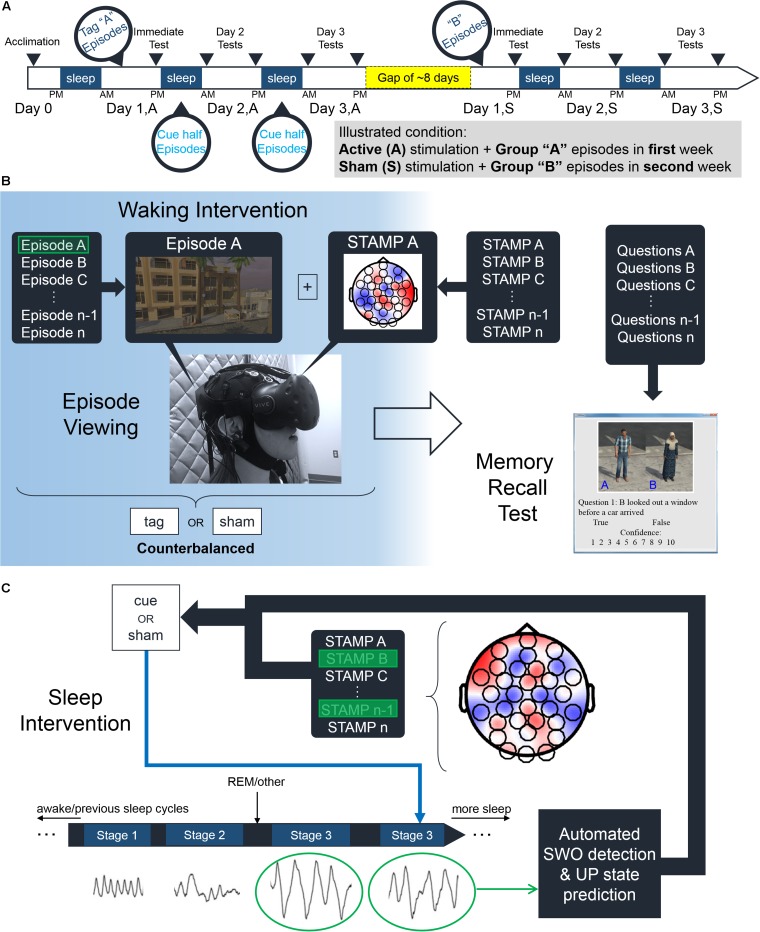
Experimental paradigm. **(A)** Subjects participated in a within-subjects counterbalanced study over 2 weeks. After an initial acclimation night, subjects viewed various episodes belonging to either “Group A” or “Group B” episodes in an immersive virtual reality environment. These episodes were accompanied by either “Active” or “Sham” STAMPs with half of those repeating during sleep the following two nights. After a gap of about 8 days, subjects viewed the episodes from the remaining Group (e.g., “Group B” if they viewed “Group A” before) and received the other stimulation condition (e.g., “Sham” if they received “Active” before). Memory recall of the episodes was assessed in five tests over the course of the 48 h of each stimulation condition before and after the gap. **(B)** Illustration of the experimental procedures during waking. For the “Active” stimulation condition, subjects were stimulated with a randomly chosen STAMP (from the library of 14) to temporally coincide with the viewing of each episode from their assigned group. Subjects in the “Sham” stimulation condition did not receive any currents as such. **(C)** Illustration of the experimental procedures during sleep. For the “Active” stimulation condition, half of the STAMPs used to tag episodes during viewing were re-applied to temporally coincide with predicted UP states of automatically detected SWOs.

**FIGURE 2:**
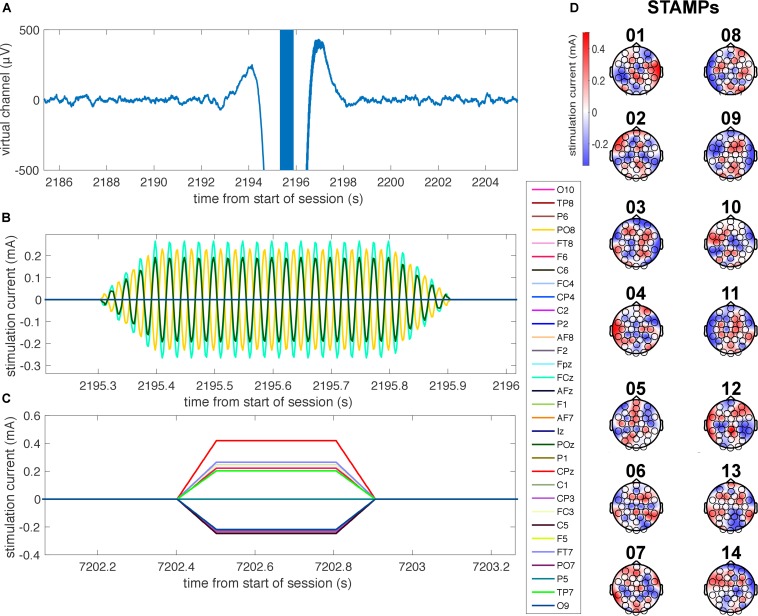
Illustration of STAMPs used in the experiment. **(A)** Time-locked plot of the EEG virtual channel, bandpass filtered between 0.5 and 50 Hz, as a 40 Hz tACS STAMP is applied during SWOs for a representative subject. Note the high-amplitude stimulation artifacts surrounding the application of the STAMP. **(B)** Time-locked plot of the applied currents for the 40 Hz tACS STAMP shown in **A** at four electrode locations (FCz, AF8, O9, and POz), with 100 ms up and down ramps. **(C)** Plot of the applied currents for a representative tDCS STAMP, with 100 ms up and down ramps. **(D)** Scalp topographies of the amplitudes of currents for the 14 STAMPs that were used in the experiment to tag episodic memories.

The experiment was conducted over the course of seven days and included five nights in our sleep laboratory that comprised an acclimation night and four experimental nights (see Figure 1A). Two experimental nights followed the acclimation night, whereas the other two took place about 8 days (mean = 8.25, standard deviation = 4.92) later. *N* = 24 subjects were randomly assigned to one of four groups in a within-subjects, counterbalanced, single-blind design based on which episode group (“A,” “B”) and which stimulation condition (“Active,” “Sham”) were employed in the first week. For the “Active” stimulation condition, each of the 14 episodes was stimulated with a unique STAMP once during viewing (see Figure 1B). The pairing between the 14 STAMPs and episodes was arbitrary and randomly chosen for each subject. Only the STAMPs that were employed for tagging one of the two episode subgroups were re-applied during slow-wave oscillations (SWOs) through the subsequent two experimental nights (see Figure 1C). The corresponding half of episodes were termed “Tag & Cue,” and the other half of the episodes were termed “Tag & No Cue.” For the “Sham” stimulation condition, the 14 episodes were neither tagged during waking nor cued during sleep. Note that subjects were also counterbalanced in terms of which of the four episode subgroups was tagged and cued. See Figure 2 for an illustration of the STAMP intervention and the scalp topography of the 14 STAMPs.

For each stimulation condition, memory performance was assessed across 3 days over the course of 48 h: immediately following initial viewing of the episodes (termed “Day 1”), following Night 1 and prior to Night 2 (termed “Day 2”), and following Night 2 and prior to Night 3 (termed “Day 3”). To assess memory recall, subjects determined the veracity of two to four declarative statements for each of the 14 episodes by recalling the underlying story (see Figure 1B), and also rated their confidence in the correctness of their responses on a scale from 1 to 10. Textual prompts and pictures of characters were the only cues available to recall the pertinent episodes. Subjects were instructed to respond as quickly as possible without sacrificing accuracy. The presentation order of questions within each day of testing was pre-randomized such that a subject would answer one question for all 14 episodes before they would see the next question for a given episode. The pre-randomization was performed at the experiment outset such that every subject received questions in the same order for a given episode group.

Memory recall was assessed via a custom graphical user interface (GUI) coded in MATLAB and administered in the sleep laboratory on the same computer used for the VR episode viewing. The GUI was a single application window that cycled through three sections as the subject answered questions. The first section the subject encountered for an individual question contained a textual prompt that described the episode this question pertained to and a button labeled “View Question” that the subject pressed when they were ready to proceed to the next section. The second section was divided into two panels. The first panel contained the same textual prompt from the previous screen, a list of the two to four characters from the episode presented in a pre-randomized order with a name (e.g., “Character B”) and a 99 × 142 pixel image in a neutral location and position, a question pertaining to a specific detail of the episode, and two radio buttons for “True” or “False” selection. The second panel contained 10 radio buttons for the subject to rate their confidence in the answer they provided in the first panel with 1 being “Least Confident” and 10 being “Most Confident.” There was no deadline for these responses. Finally, a button labeled “Next Question” became active once the subject had answered the question and provided a confidence rating. This button led to a third screen that enforced a 4 s interval between questions and then automatically loaded the prompt screen of the next question. This process repeated until all questions for the session had been answered. No feedback on the accuracy of their answers was provided to the subjects at any time during the experiment. Subjects were instructed to respond as quickly as possible without sacrificing accuracy.

Data were automatically collected from the GUI during user interaction and saved for later analysis. Five data points were captured for each question: the “True”/“False” response, the confidence rating, the prompt screen viewing duration, the interval from the question screen presentation to the selection of a “True”/“False” response, and the interval from the question screen presentation to the selection of a confidence rating. Supplementary Movie S2 demonstrates the GUI used to assess the memory recall.

### Type-2 ROC Curve Analysis

**FIGURE 3:**
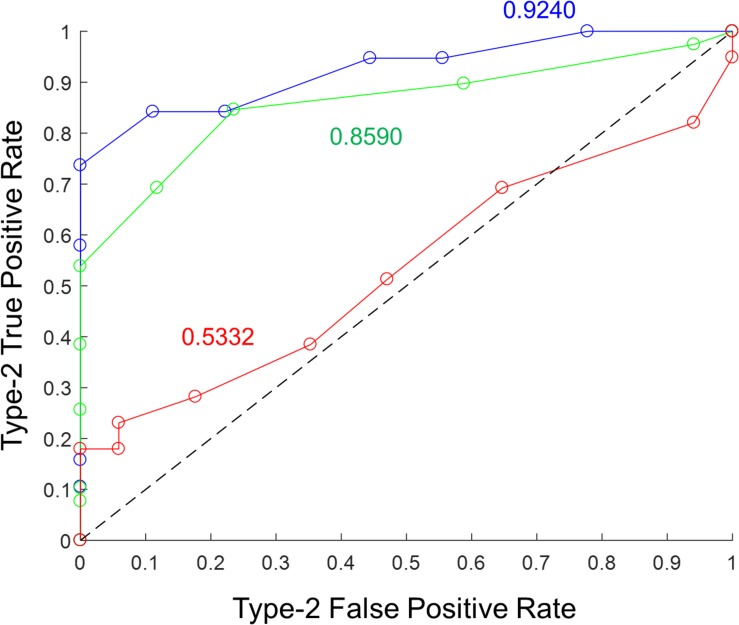
Illustration of Type-2 ROC curve analysis to assess episodic metamemory. Type-2 ROC curves from the 3 days of testing are shown for a representative subject in the “Sham” stimulation condition. Blue corresponds to “Day 1,” green to “Day 2,” and red to “Day 3.” The corresponding metamemory score (calculated as the area under the curve) is shown in the matching color.

Metamemory was calculated empirically from correct and incorrect recalls and the corresponding confidence ratings using standard procedure (Galvin et al., 2003; MacMillan and Creelman, 2005; Fleming and Lau, 2014). In each memory recall trial, the textual prompt and pictures of characters would help retrieve the corresponding episode. The declarative statement would then be matched with the retrieved episode to determine its veracity. The stronger the memory recall, the greater is the ability to correctly recall the events from the episode without confabulating any details. A correctly answered declarative statement (irrespective of the response category) was considered a Type-2 true positive, and an incorrectly answered declarative statement (irrespective of the response category) a Type-2 false positive. Probability density functions of confidence ratings (on the scale from 1 to 10) with 10 bins were computed for Type-2 true positives (i.e., correct recalls) and for Type-2 false positives (i.e., error recalls) separately. Next, the cumulative distribution functions were calculated for each in reverse direction (from end to beginning). The data points of the cumulative distribution function for Type-2 true positives were plotted against the corresponding data points of the cumulative distribution function for Type-2 false positives to obtain the Type-2 ROC curve. The area under the Type-2 ROC curve was employed as the metric of metamemory for the episodic memory recall (see Figure 3). The metric is essentially the probability that a randomly chosen correct recall has a higher confidence rating than a randomly chosen incorrect recall. As the ROC curve analysis is based on the computation of probability density functions, the data from morning and evening tests were combined for Days 2 and 3 to maximize the accuracy of the estimate of metamemory.

### Experimental Procedure

At the orientation session before the experiment began, subjects were invited to provide informed consent. Head measurements were also made (circumference, nasion to inion, and pre-auricular to pre-auricular) to fit a neoprene head cap. Subjects were next given a tour of the sleep laboratories and an explanation of the electroencephalography (EEG)/tES equipment and experimental procedures.

For the acclimation night, subjects were prepped and fitted with a neoprene head cap for polysomnographic (PSG) recording during sleep. EEG electrode locations were digitized using Polhemus FASTRAK System (Polhemus, Inc.) for data analysis purposes as well as to measure how much the cap may have shifted during the subsequent sleep session. Subjects were instructed to lie down in a supine position at approximately 22:00, when biocalibrations were performed to help identify sources of noise in later EEG acquisition. This included EEG data collection of eyes open for 1 min, closed for 1 min, looking up, down, right, and left, blinking slowly five times, clenching the jaw, and finally moving into a comfortable sleeping position. Lights out for the subjects occurred between 22:00–23:00, and they slept for up to 8 uninterrupted hours before being awoken. Upon waking, subjects could use the restroom and were offered water and snacks. They were then disconnected from the EEG/tES hardware and released.

In the evening of the acclimation night, subjects were familiarized with the VR environment and task procedures via a short practice session. During this session, subjects viewed one 3.5-min long example episode in the virtual world and then completed a 10-question sample memory recall test on a personal computer. Apart from acclimatizing the subjects to sleeping in the laboratory, the acclimation night was also leveraged for a pilot study to assess the effects of closed-loop tACS on a paired associates task, unrelated to the main experiment. Note that including this as a covariate did not change any of the findings reported in this paper.

The main experiment began the following evening. For the first experimental night, subjects arrived at the laboratory at approximately 19:00 and were immediately prepped for EEG data collection and STAMP stimulation. The subjects then sat in front of the computer, put on the HTC Vive^®^ headset, and heard the task instructions. They viewed 14 episodes from either Group A or B, depending on their assignment, in a random order within the VR environment. Following episode viewing, subjects rated three different types of sensations (itching, heat/burning, and tingling) on a 0–10 Likert scale, where 0 indicated no sensation at all and 10 indicated the most intense possible sensation. Any report of 7 or above would have resulted in immediate termination of the experiment without penalty to the subject. No subjects were lost due to this reason.

Next, subjects completed a test to assess their baseline memory recall. They were then prepped for PSG recording during sleep at roughly 21:00. Lights out occurred between 22:00 and 23:00, and rise time was between 06:00 and 07:00. During sleep, a trained research assistant monitored EEG data and started the cueing algorithm after observing 4 min of continuous “N2/N3” sleep, which was then allowed to run through the remainder of the night. Subjects either received “Active” (2.5 mA) or “Sham” (no current) STAMPs during predicted UP states for the entire duration of sleep. Stimulation was paused if the subject showed signs of waking and resumed after the subject was again in “N2/N3” sleep. Upon waking, subjects completed another test to assess the effect of sleep stimulation on memory recall. They were then disconnected from the EEG/tES hardware and released.

For the second experimental night, the PSG setup and STAMP stimulation procedures were identical to the first experimental night. However, subjects did not view the episodes from the previous evening again, but they completed two memory recall tests – one prior to sleep and the other upon waking. For the follow-up to the second experimental night, subjects arrived approximately 24 h after their previous day arrival (19:00), were prepped for EEG data collection, and completed a final test to assess the effect of sleep stimulation on more long-term memory recall.

After about 8 days, subjects returned to the laboratory for their third and fourth experiment nights, succeeded by the final follow-up. The timeline and procedures were identical to the first and second experimental nights and their follow-up, the only differences being the group of episodes viewed in the VR (“A,” “B”) and the stimulation condition (“Active,” “Sham”) were opposite of their assignments for the first and second experimental nights. Upon completion of the follow-up to the fourth experimental night, the subjects were debriefed about the experiment.

Only two subjects dropped out of the study, both of whom received “Active” stimulation in the first week. Only one of those opted out due to stimulation (in the last evening test for the “Active” stimulation condition); the other stopped due to unspecified reasons (in the first evening test for the “Active” stimulation condition).

### Waking Electroencephalographic (EEG) Data Collection

32-channel physiological data collection and simultaneous 32-channel stimulation were conducted using the StarStim64 device (Neuroelectrics, Inc.). The 64 electrodes were held in place using a neoprene head cap, according to the international 10-10 system (recording: *P7*, T7, *CP5*, *FC5*, *F7*, F3, *C3*, *P3*, *FC1*, *CP1*, *Pz*, PO4, *O2*, Oz, *O1*, PO3, *CP2*, *Cz*, *FC2*, *Fz*, AF3, *FP1*, *FP2*, AF4, *P8*, T8, *CP6*, *FC6*, *F8*, F4, *C4*, *P4*; stimulation: O10, TP8, P6, PO8, FT8, F6, C6, FC4, CP4, C2, P2, AF8, F2, FPz, FCz, AFz, F1, AF7, Iz, POz, P1, CPz, C1, CP3, FC3, C5, F5, FT7, PO7, P5, TP7, O9). Solidgeltrodes (NE028, Neuroelectrics, Inc.) and pistim electrodes (NE024, Neuroelectrics, Inc.) were used for physiological data collection and stimulation, respectively. EEG data were collected from 23 of these 32 sites (marked in italics above). The remaining electrodes (PO3, PO4, Oz, AF3, AF4, F3, F4, T7, T8) were repurposed to record electrocardiogram (ECG), electrooculogram (EOG), and electromyogram (EMG) to allow for the detection of artifacts and sleep stages. An ECG lead (PO3) was placed under the left collarbone, and both vertical (AF3) and horizontal (AF4) EOG were collected: one lead placed superior and lateral to the right outer canthus, and another lead inferior and lateral to the left outer canthus. The physiological data were sampled at 500 Hz. Common Mode Signal (CMS) and Driving Right Leg (DRL) reference electrodes (stricktrodes: NE025, Neuroelectrics, Inc.) were placed on the right preauricular. No online hardware filtering, except for line noise (60 Hz), was applied during collection.

### Polysomnographic (PSG) Data Collection

For PSG data collection during sleep, the setup was nearly identical to wake, with a few exceptions. First, two EMG electrodes were placed on and under the chin in accordance with PSG recording guidelines set forth by the American Academy for Sleep Medicine (Berry et al., 2012) to help with sleep scoring. Second, data were collected from 25 EEG electrodes, of which C3, C4, O1, O2, F3, and F4 were used for sleep staging.

### Waking STAMP Stimulation

For the “Active” stimulation condition, STAMP montages were delivered via the StarStim64 device (Neuroelectrics, Inc.) during the one-shot viewing of episodes in the VR. For all subjects, each episode was randomly assigned a unique STAMP from the set of 14 montages. A given STAMP was applied for the entire duration of the corresponding episode (about a minute long) with ramp up and ramp down times of 100 ms. Inter-episode interval was randomly sampled between 6 and 8 s. We employed tDCS STAMPs for eight subjects and 40 Hz tACS STAMPs for the remaining 16 subjects to demonstrate the generality of the concept of using spatiotemporal stimulation patterns for memory tagging and cueing.

### Stimulation During Slow-Wave Oscillations

Our stimulation algorithm was automatically triggered through the whole night to transiently apply “Tag & Cue” STAMPs during putative UP states of SWOs. The algorithm first detects the presence of SWOs, which consist of low-frequency synchronized upward and downward deflections of EEG. It next attempts to determine the frequency and phase of ongoing endogenous SWOs. For robust SWO detection, a virtual channel is computed by averaging 13 fronto-parieto-central EEG channels (Cz, FC1, FC2, CP1, CP2, Fz, C4, Pz, C3, F3, F4, P3, P4 in the international 10-10 system) to determine the overall synchronous activity of EEG recorded during sleep. The virtual channel allows the observation of moments of relatively high SW power, referred to as SW events, while averaging out activity of lesser magnitudes on individual channels unrelated to the pattern of SWOs. The included channels are stored in a running 5 s buffer. They undergo moving average subtraction with a 1 s window (to mean center the signals at 0 μV), and noisy channels exceeding 500 μV min-to-max amplitude across the 5 s are rejected before the virtual channel is computed. The buffer is updated with each discrete data fetch operation that gets the new latest data up to the point of data request. By the time the buffer is updated, there is a random transmission delay, which needs to be accounted for to plan and precisely time the stimulation in the near future.

The virtual channel data in the buffer are further processed to detect the presence of SWOs and predict a putative UP state. The algorithm applies a fast Fourier transform (FFT) to the buffered data to determine the power spectrum. Stimulation is planned when the ratio of the cumulative power in the SW band (0.5–1.2 Hz) is more than 30% of the total cumulative power from 0.1 to 250 Hz. If this SW relative power threshold is crossed, the data are bandpass filtered in the SW band with a second-order zero-lag Butterworth filter. The Hilbert transform is then applied to the filtered signal to obtain the analytic signal, and the phase of the analytic signal is shifted back by 90° to align it with the SWOs. Next a sine wave is fit to the imaginary component of this signal by optimizing the amplitude, offset, and phase parameter values and using the dominant frequency in the SW band from the power spectrum. The sine wave is then projected into the future, identifying the temporal targets that would synchronize STAMPs to the predicted endogenous SWOs. Throughout this process, the dynamic latency associated with data processing is timed using the system clock. Together with distributions of calibrated latencies for data fetch and stimulation commands (mean = 5 ms, standard deviation = 2 ms), which were measured offline, the algorithm estimates the correct time point to communicate with the hardware to initiate the stimulation. As an example, suppose that at a given moment the algorithm initiates data fetch to populate the buffer with the last 5 s of EEG data. The data then become available for processing a few ms (say, 6 ms) into the future based on sampling from the distribution for data fetch latency. Assume it then takes 100 ms for data processing to predict that the next putative UP state will occur 600 ms in the future from the starting time point. If it takes a few ms (say, 7 ms) to physically initiate stimulation based on sampling from the distribution for stimulation command latency, the algorithm will wait 487 ms (600 – 100 – 7 – 6 ms) after the EEG processing step to send the stimulation command to the device.

During “Active” nights, STAMPs assigned to “Tag & Cue” episodes were administered during SWOs through the sleep period to boost the probability of specific memory replays. Sometimes, due to hardware and/or processing delays, the targeted start of the stimulation was not possible. In these cases, the algorithm compared the current time to the (now deprecated) stimulation start time, and checked if at least 300 ms of stimulation was still possible within the putative UP state. If so, the stimulation was initiated immediately and continued through the remainder of the putative UP state. If this was not possible, the algorithm started stimulation at the next putative UP state based on further projection of the sine wave fit from the buffer. Once STAMP delivery was completed (i.e., after stimulation offset), the system idled for 3 s to avoid the collection of high-amplitude stimulation artifacts in the data buffer, then resumed the cycle of data update in the buffer in search of the next SW event, at which point another STAMP was administered, and so on. STAMPs had ramp up and ramp down times of 100 ms during sleep as well. A minimum interval of 8 s was imposed between two consecutive SW events. Further, the seven STAMPs for the “Tag & Cue” episodes were always applied sequentially in a batch of seven consecutive SW events, with their order randomized across batches through the sleep period. For the “Sham” stimulation condition, the same algorithm was applied to mark the potential stimulation times without any currents being actually applied.

### *Post hoc* Sleep EEG Analyses

For sleep staging, EEG data of electrodes C3, C4, O1, O2, Fp1, and Fp2 were bandpass filtered between 0.5 and 35 Hz, together with EMG data between 10 and 100 Hz. Each 30 s epoch was visually inspected by an experienced technician and assigned a stage of “Wake,” “N1,” “N2,” “N3/SWS,” “REM,” or “Movement” according to guidelines by the American Academy for Sleep Medicine (Berry et al., 2012). Time in each sleep stage was directly derived by summing up all epochs determined to belong to that sleep stage. Sleep efficiency was computed as the percentage of the sleep period that was spent in any sleep stage other than “Wake.” Note that analyses of sleep stage distributions and sleep efficiency did not include a subset of subjects from each experimental night for whom more than 15% of their sleep period was not scorable.

For sleep EEG biomarker analyses, the data were analyzed with custom-built scripts implemented in Matlab R2016a (The MathWorks) utilizing FieldTrip (Oostenveld et al., 2011) and EEGLab (Delorme and Makeig, 2004) functions. The data were epoched into pre- and post-SW event windows: pre-SW event windows captured −6.4 to −0 s before the onset of SW events, and post-SW event windows captured 0 to 12.8 s after the offset of SW events. For each epoch, a first-pass artifact correction procedure that identified large amplitude artifacts was performed by searching for peak-to-peak voltage changes of 500 μV within each channel in 200 ms sliding windows, and interpolating any segment that crossed this threshold using non-artifact time points before and after the segment. If more than 25% of segments of a time series of a channel was marked for correction, then the entire epoch for that channel was interpolated using data from neighboring channels. If 80% or more of the channels exceeded the 25% segment threshold, then the epoch was discarded entirely. A second-pass artifact correction was then performed such that any channel that exceeded the 500 μV (peak-to-peak voltage change) threshold across the time series within a trial was reconstructed by interpolation of its neighbors.

Following artifact correction, trials were selected with the constraint that each trial had enough usable data both pre- and post-SW event to have good time-frequency estimates of the lowest frequency of interest (i.e., 0.5 Hz); otherwise, they were rejected. All epochs were then truncated to −6.4 to −1 s before the SW event and 3 to 12.8 s after the SW event in order to avoid high-amplitude stimulation artifacts. This same truncation was applied to data from the “Sham” nights. Finally, all epochs were mean centered, bandpass filtered with a Butterworth filter between 0.1 and 125 Hz, and bandstop filtered between 59 and 61 Hz, and all channels were re-referenced to the global average across channels.

Time-frequency decomposition of the data was performed in FieldTrip. Prior to decomposition, symmetric (mirror) padding was applied to extend the pre- and post-SW event time series to reduce edge artifacts. EEG epochs were then convolved with Morlet wavelets starting with a width of 4 at the center frequency of 0.5 Hz and increasing in width logarithmically up to a maximum width of 7 in order to minimize the combined uncertainty in time and frequency domains. Simultaneously, subsequent center frequencies were chosen such that each wavelet was one standard deviation in frequency domain from the previous wavelet. This process resulted in a time-frequency representation of roughly 35 log-spaced frequency bins from 0.5 to 50 Hz and equally-spaced time bins of 20 ms. Once time-frequency data were calculated, pre-SW event data from −3.5 to −3 s in each frequency bin were concatenated across trials and used to estimate a mean and standard deviation. These values were then used as a baseline to *z*-score both the pre- and post-SW event power for each trial and within each frequency bin to compute spectral power changes without single-trial bias (Ciuparu and Mureşan, 2016). These values were then averaged across trials within “Active” and “Sham” nights to yield a single channel × time × frequency matrix per condition for each subject. Subject averages were created using a random subset of trials such that trial numbers were matched between the “Active” and “Sham” nights. Note that sleep EEG biomarker analyses could not be run on a subset of the subjects due of the lack of usable data from “Sham” nights despite artifact correction.

Significant differences in spectral power changes and correlations with behavior were assessed statistically using non-parametric cluster-based permutation tests to correct for multiple comparisons in the channel x time space (Maris and Oostenveld, 2007). Average spectral power changes were calculated within pre-defined frequency bands: SW (0.5–1.2 Hz), delta (1–4 Hz), theta (4–8 Hz), slow-spindle (8–12 Hz), and fast-spindle (12–15 Hz). For each frequency band, a paired-sample *t*-test was performed at each channel × time bin between 3 and 10 s from the offset of SW events, and clusters were created by grouping adjacent channels and time bins that had a *P*-value < 0.05. Each cluster was then characterized by the sum of its *t*-values, and cluster-level statistics were evaluated using a permutation distribution created by shuffling the subject labels and repeating the clustering procedure 2000 times to correct for multiple comparisons. Thus, a clusterwise significance value can be attributed to each observed cluster in reference to its position in the permutation-based surrogate distribution. Any cluster with a clusterwise *P*-value < 0.05 after a further application of Bonferroni correction for the five additional frequency band comparisons was considered significant.

Significant clusters from the first analysis (called “contrast clusters”) were then used as a mask to perform a second cluster-based permutation test on the correlation between the differences in overnight metamemory changes for “Tag & Cue” and “Sham” episodes and the corresponding differences in average spectral power change following SW events. This effectively limited the second cluster-based analysis to the channel × time bins that showed an *a priori* significant difference between the “Active” and “Sham” nights. For each “contrast cluster,” a correlation coefficient and the related *P*-value were calculated at each constituent channel × time bin. The same non-parametric cluster-based permutation test was then performed to group adjacent channel × time bins with *P*-values < 0.05 into so-called “correlation clusters” with a clusterwise *P*-value < 0.05.

## Results

### Sleep Architecture

We first analyzed the potential effects of STAMPs on the sleep architecture (i.e., time spent in various sleep stages) during the experimental nights using a linear mixed-effects model with subject as a random factor and with fixed factors for stimulation condition (“Active,” “Sham”), experimental night (“Night 1,” “Night 2”), sleep stage (“Wake,” “N1,” “N2,” “N3/SWS,” “REM,” “Movement”), all possible interactions among them, and covariates of stimulation condition order (“Active First,” “Sham First”) and STAMP type (“tDCS,” “tACS”). We only found a marginally significant effect of stimulation condition [*F*(1,394) = 3.66, *p* = 0.056] and a significant effect of sleep stage [*F*(5,394) = 1506.97, *p* < 2e-16]. All other effects were not significant. Importantly, there were no interactions involving stimulation condition and sleep stage. We also examined the potential effects of STAMPs on sleep efficiency using a similar linear mixed-effects model with subject as a random factor and with fixed factors for stimulation condition (“Active,” “Sham”), experimental night (“Night 1,” “Night 2”), an interaction between them, and covariates of stimulation condition order (“Active First,” “Sham First”) and STAMP type (“tDCS,” “tACS”). We did not find any significant effects. These results suggest that STAMPs did not modulate the sleep stage distributions or sleep efficiency (see Table 1).

**TABLE 1:** Sleep scoring statistics.

	**Night 1**		**Night 2**	
	**Active**	**Sham**	**Active**	**Sham**
	**(*N* = 16)**	**(*N* = 18)**	**(*N* = 16)**	**(*N* = 20)**
Wake	14.75 (4.59)	27.89 (8.60)	29 (6.25)	27.1 (4.79)
N1	13.75 (1.98)	18.92 (1.57)	15.31 (1.98)	20 (1.73)
N2	303.22 (8.54)	291.72 (8.92)	290.88 (8.60)	291.3 (7.23)
N3/SWS	89.28 (5.88)	92.75 (5.97)	80.03 (6.02)	85.68 (3.81)
REM	59.91 (6.65)	69.64 (6.73)	57.41 (6.64)	80.75 (3.30)
Movement	2.88 (0.95)	2.72 (0.52)	2.31 (0.63)	3.3 (0.63)
Sleep efficiency	97.05%	94.43%	93.92%	94.63%
	(0.87%)	(1.74%)	(1.33%)	(0.99%)

*Mean and standard error of mean of the amount of time (in minutes) spent in various sleep stages and of sleep efficiency (%) for the two experimental nights and the two stimulation conditions across subjects.*

Further, we analyzed the number of SW events during sleep using a linear mixed-effects model with subject as a random factor and with fixed factors for stimulation condition (“Active,” “Sham”), experimental night (“Night 1,” “Night 2”), sleep stage (“Wake,” “N1,” “N2,” “N3/SWS,” “REM,” “Movement”), all possible interactions among them, and covariates of stimulation condition order (“Active First,” “Sham First”) and STAMP type (“tDCS,” “tACS”). We only found a significant effect of sleep stage [*F*(5,337.03) = 171.71, *p* < 2e-16]. All other effects were not significant. As SWOs occur predominantly during NREM sleep stages 2 and 3 (Rasch and Born, 2013), we re-ran the above linear mixed-effects model with the sum for “N2” and “N3/SWS” contrasted with the sum for all the other sleep stages. We found only a significant effect of the lumped sleep stage [*F*(1,116) = 230.97, *p* < 2e-16], with the number of SW events about 7× more likely to occur during “N2” and “N3/SWS” compared to all other stages combined. These results verify that the automated SWO detection was sufficiently accurate for the application of STAMPs (see Table 2).

**TABLE 2:** Validation of the application of STAMPs during SWOs in NREM sleep stages 2 and 3.

**Sleep stage**	**Night 1**		**Night 2**	
	**Active**	**Sham**	**Active**	**Sham**
Wake	7.71 (4.74)	58.31 (20.42)	54.67 (28.16)	76.22 (24.37)
N1	29.79 (7.17)	51.13 (11.67)	39.93 (8.30)	48.78 (7.31)
N2	997.43 (122.34)	800.38 (89.70)	859.47 (110.65)	813.78 (53.93)
N3/SWS	668.93 (114.61)	597.56 (82.77)	537.33 (71.76)	538.67 (52.59)
REM	183.71 (59)	94.75 (21.075)	122.87 (34.85)	114.78 (25.43)
Movement	8.43 (4.40)	9.75 (3.18)	5.2 (1.83)	11.44 (2.24)

*Mean and standard error of mean of the number of SW events in various sleep stages for the two experimental nights and the two stimulation conditions across subjects.*

### Waking Sensations

We next investigated the effects of STAMPs on sensations during episode viewing using a repeated measures ANOVA with two within-subjects factors (sensation type: “itching,” “heat/burning,” “tingling”; stimulation condition: “Active,” “Sham”) and two between-subjects factors (STAMP type: “tDCS,” “tACS”; stimulation condition order: “Active First,” “Sham First”). We found a significant effect of stimulation condition [*F*(1,20) = 28.50, *p* = 0.000032], with the sensations during “Active” stimulation rated higher by 1.58 compared to “Sham” when collapsed across sensation types. All other effects were not significant, including between tDCS and tACS STAMPs. Numerically tACS STAMP sensations were rated on average lower than tDCS STAMP sensations when collapsed across within-subject factors. The overall mean values for sensations were below 2.5 out of 10 in the “Active” stimulation condition and below 1 out of 10 in the “Sham” stimulation condition, which are much less than the threshold score of 7 on the 0–10 Likert scale for an intense sensation, suggesting that STAMPs were well tolerated.

Further, we ran a series of chi-squared tests to investigate whether subjects were blind to the stimulation conditions. Due to the within-subjects nature of the design, we assessed if subjects were able to guess both stimulation conditions successfully, which was not the case [χ^2^(1) = 0.053, *p* = 0.82]. We then looked at each stimulation condition separately, regardless of order. For the “Active” stimulation condition, subjects were not able to guess their condition successfully [χ^2^(1) = 0.18, *p* = 0.67]. For the “Sham” stimulation condition, however, all subjects guessed their condition successfully; so a chi-squared test could not be performed. Because of this, we looked at stimulation condition order effects (“Active First” vs. “Sham First”). “Active First” subjects were not able to guess their stimulation conditions successfully [χ^2^(1) = 1.60, *p* = 0.21], whereas “Sham First” subjects were able to do so at a trend level [χ^2^(1) = 2.78, *p* = 0.096]. Overall these results suggest that the subjects were sufficiently blind to the stimulation condition and order assignments.

### Absolute Accuracy

For behavior, we first examined absolute accuracy scores using a linear mixed-effects model with subject as a random factor. Fixed effects included intervention type (“Tag & Cue,” “Tag & No Cue,” “Sham”), day (“Day 2,” “Day 3”), the interaction of intervention type and day, and covariates of baseline performance (“Day 1”), STAMP type (“tDCS,” “tACS”), stimulation condition order (“Active First,” “Sham First”), episode group (“Group A,” “Group B”) in the first week, and episode subgroup that was tagged and cued (“Subgroup 1,” “Subgroup 2”). We only found a significant effect of baseline performance [*F*(1,79.92) = 25.33, *p* = 2.92e-6] and a marginally significant effect of intervention type [*F*(2,104.015) = 2.77, *p* = 0.067]. All other effects were not significant. Given the marginally significant effect of intervention type, we performed two-tailed paired-sample *t*-tests on absolute accuracy scores averaged over Day 2 and Day 3. The measures for “Tag & Cue” episodes were not significantly different from those for both “Tag & No Cue” [*t*(23) = −0.69, adjusted *p* = 0.50, Holm–Bonferroni correction for two comparisons] and “Sham” [*t*(23) = −1.77, adjusted *p* = 0.18, Holm–Bonferroni correction for two comparisons] episodes. While not significant, the absolute accuracy for “Tag & No Cue” and “Sham” episodes averaged over Day 2 and Day 3 and across subjects was numerically higher than that for “Tag & Cue” episodes by 1.83 and 4.58%, respectively. Overall these results suggest that STAMPs did not selectively modulate the absolute accuracy of memory recall as such.

### Metamemory

**FIGURE 4:**
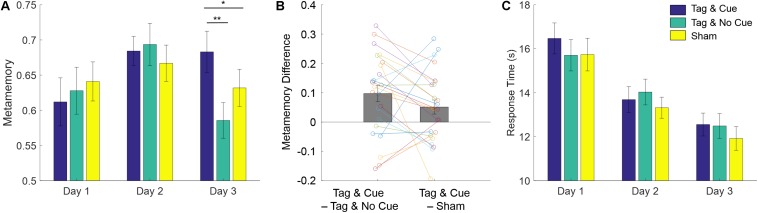
STAMP tagging and cueing enhances episodic metamemory without a speed–accuracy trade-off. **(A)** Metamemory performance on the episodic memory task is reported across the 3 days of testing. Blue bars show the metamemory scores for “Tag & Cue” episodes, green for “Tag & No Cue” episodes, and yellow for “Sham” episodes. As shown in Figure 3, metamemory is computed as the area under the Type-2 ROC curve (0-1). Metamemory scores for “Tag & Cue” episodes are significantly higher than those for “Tag & No Cue” by 19.43% (*p* = 3.796e-3, corrected; paired-sample Cohen’s *d* = 0.72) or “Sham” episodes by 10.01% (*p* = 0.048, corrected; paired-sample Cohen’s *d* = 0.43) on Day 3. The markers correspond to data from individual subjects for Day 3. **(B)** Paired differences in metamemory on Day 3 from individual subjects with respect to “Tag & Cue” episodes. **(C)** Response time (RT) decreased across the 3 days of testing, but there were no significant differences among the intervention types. Error bars represent ± 1 SEM (*N* = 24).

We next analyzed the metamemory scores (see Figure 4A and Table 3) using a linear mixed-effects model with the same fixed and random effects as above. Similar to absolute accuracy, we found marginally significant effects of baseline performance [*F*(1,129.69) = 2.75, *p* = 0.10] and intervention type [*F*(2,113.18) = 2.90, *p* = 0.059]. But unlike absolute accuracy, metamemory significantly differed between days [*F*(1,113.091) = 8.51, *p* = 0.0043], and there was a significant interaction between intervention type and day [*F*(2,113.091) = 3.66, *p* = 0.029]. Based on the significant interaction between intervention type and day, we ran follow-up linear mixed-effects models for each day separately with intervention type and subject as fixed and random factors, respectively. For Day 2, there were no significant effects. However, for Day 3, we found a significant effect of intervention type [*F*(2,46) = 6.35, *p* = 0.0037]. Thus, the metamemory scores differed significantly across the intervention types by Day 3 following the application of “Tag & Cue” STAMPs during two consecutive nights.

**TABLE 3:** Results of the linear mixed-effects model for the effects of STAMPs on metamemory.

**Effect**	**NumDF**	**DenDF**	***F*-value**	***P*-value**
Baseline performance	1	129.69	2.75	0.10
STAMP type	1	17.97	0.63	0.44
Stimulation condition order	1	18.011	0.19	0.67
Episode group	1	18.089	0.38	0.54
Episode subgroup	1	18.023	0.022	0.88
Intervention type	2	113.18	2.90	0.059
Day	1	113.091	8.51	**0.0043**
Intervention type × day	2	113.091	3.66	**0.029**

*The *P*-values for the significant effects are highlighted in bold.*

Our main hypotheses of interest were the performance for “Tag & Cue” episodes would be better than that for either “Tag & No Cue” or “Sham” episodes following the sleep intervention with “Tag & Cue” STAMPs. Given the significant effect of intervention type on Day 3, we performed two-tailed paired-sample *t*-tests on metamemory scores from Day 3. “Tag & Cue” metamemory scores were significantly greater than both “Tag & No Cue” [*t*(23) = 3.51, adjusted *p* = 3.79e-3, Holm–Bonferroni correction for two comparisons; paired-sample Cohen’s *d* = 0.72] and “Sham” [*t*(23) = 2.089, adjusted *p* = 0.048, Holm–Bonferroni correction for two comparisons; paired-sample Cohen’s *d* = 0.43] metamemory scores (see Figure 4B). And it turned out that “Tag & No Cue” metamemory scores were not significantly different from “Sham” metamemory scores [*t*(23) = −1.56, uncorrected *p* = 0.13]. Thus, the application of STAMPs during SWOs in the two nights following one-shot viewing led to specific enhancement of metamemory for the episodes that were both tagged and cued. The long-term benefit in metamemory for the “Tag & Cue” episodes is remarkable, considering that the episodes were only tagged once during viewing.

We also checked if mere tagging of episodes with STAMPs modulated their immediate metamemory following encoding. In particular, the baseline performance (“Day 1”) was assessed with a linear mixed-effects model with intervention type (“Tag & Cue,” “Tag & No Cue,” “Sham”) and subject as fixed and random factors, respectively. There was no significant effect of intervention type [*F*(2,118) = 0.56, *p* = 0.58], confirming that STAMP-based tagging during episode viewing did neither improve nor impair the baseline performance. To further understand the primary result, we also analyzed response times (RTs) using a linear mixed-effects model with subject as a random factor (see Figure 4C). Fixed effects included intervention type (“Tag & Cue,” “Tag & No Cue,” “Sham”), day (“Day 2,” “Day 3”), the interaction of intervention type and day, and a covariate of baseline performance (“Day 1”). We found significant effects of day [*F*(1,108.95) = 32.11, *p* = 1.21e-7] and baseline performance [*F*(1,137) = 11.75, *p* = 0.00080], and a marginally significant effect of intervention type [*F*(2,109.54) = 2.45, *p* = 0.091]. Importantly, the interaction of intervention type and day was not significant [*F*(2,108.95) = 0.25, *p* = 0.78], suggesting that the subjects responded with similar speeds across the three intervention types. This demonstrates that the benefit to metamemory on Day 3 from STAMP-based tagging and cueing was not simply due to a speed-accuracy trade-off.

### Interaction Between Pre-Sleep Metamemory and Overnight STAMPs

**FIGURE 5:**
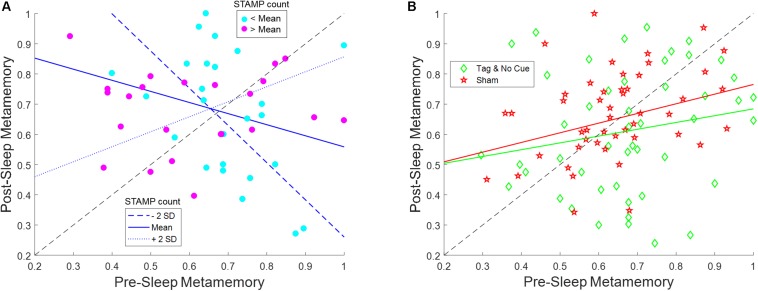
STAMP tagging and cueing on metamemory is modulated by an interaction between pre-sleep metamemory and the number of STAMP applications. **(A)** Post-sleep metamemory for “Tag & Cue” episodes depends on the interaction between the covariates of pre-sleep metamemory and STAMP count for both experimental nights. The three lines represent the linear mixed-effects model predictions for different STAMP counts (ranging from two standard deviations below the mean to two standard deviations above the mean). The markers correspond to data for “Tag & Cue” episodes from individual subjects for both experimental nights (i.e., two per night per subject), with color coding based on whether the overnight STAMP dose was lower (cyan) or greater (magenta) than the mean. **(B)** The interactions of pre-sleep metamemory with STAMP count for “Tag & No Cue” episodes, and with the number of SW events for “Sham” episodes, are not significant for both experimental nights. The lines represent the linear fits to the pertinent data. The markers correspond to data for “Tag & No Cue” (green) and “Sham” (red) episodes from individual subjects for both experimental nights (i.e., two per night per subject).

The evening pre-sleep metamemory and the number of STAMP applications in each “Active” night varied widely across the subjects, with the latter depending on the number of SW events detected. We therefore analyzed the effects and interactions of these covariate variables on the morning post-sleep metamemory for “Tag & Cue” and “Tag & No Cue” episodes within the “Active” stimulation condition using a linear mixed-effects model with subject as a random factor (see Figure 5 and Table 4). Fixed effects of this model included categorical variables of intervention type (“Tag & Cue,” “Tag & No Cue”) and experimental night (“Night 1,” “Night 2”), continuous variables of pre-sleep metamemory and the number of STAMP applications, and all possible interactions among them. We found a marginally significant effect of pre-sleep metamemory [*F*(1,79.009) = 3.61, *p* = 0.061], significant effects of intervention type [*F*(1,69.77) = 10.83, *p* = 0.0016], a marginally significant two-way interaction of pre-sleep metamemory and STAMP count [*F*(1,78.008) = 3.24, *p* = 0.076], significant two-way interactions of intervention type and STAMP count [*F*(1,68.19) = 6.17, *p* = 0.015] and of intervention type and pre-sleep metamemory [*F*(1,70.43) = 9.33, *p* = 0.0032], and a significant three-way interaction of intervention type, pre-sleep metamemory, and STAMP count [*F*(1,68.90) = 5.73, *p* = 0.019]. All other effects were not significant. In particular, post-sleep metamemory was not modulated by experimental night.

**TABLE 4:** Results of the linear mixed-effects model for the interaction between pre-sleep metamemory and the number of STAMP applications on post-sleep metamemory.

**Effect**	**NumDF**	**DenDF**	***F*-value**	***P*-value**
Intervention type	1	69.77	10.83	**0.0016**
Night	1	66.64	0.81	0.37
Pre-sleep metamemory	1	79.009	3.61	0.061
STAMP count	1	79.69	2.44	0.12
Intervention type × night	1	65.92	0.28	0.60
Intervention type × pre-sleep metamemory	1	70.43	9.33	**0.0032**
Intervention type × STAMP count	1	68.19	6.17	**0.015**
Night × pre-sleep metamemory	1	66.79	0.46	0.50
Night × STAMP count	1	67.34	1.06	0.31
Pre-sleep metamemory × STAMP count	1	78.008	3.24	0.076
Intervention type × night × pre-sleep metamemory	1	66.31	0.38	0.54
Intervention type × night × STAMP count	1	69.49	0.0003	0.99
Intervention type × pre-sleep metamemory × STAMP count	1	68.90	5.73	**0.019**
Night × pre-sleep metamemory × STAMP count	1	67.75	0.30	0.59
Intervention type × night × pre-sleep metamemory × STAMP count	1	70.21	0.066	0.80

*The *P-*values for the significant effects are highlighted in bold.*

Based on these results, we ran follow-up linear mixed-effects models for “Tag & Cue” and “Tag & No Cue” episodes separately with subject as a random factor and with pre-sleep metamemory, STAMP count, and the interaction of pre-sleep metamemory and STAMP count as fixed effects. There were no significant effects for “Tag & No Cue” episodes (see Figure 5B). However, for “Tag & Cue” episodes, we found significant effects of both pre-sleep metamemory [*F*(1,42.24) = 8.40, *p* = 0.0059] and STAMP count [*F*(1,39.72) = 6.86, *p* = 0.012], and a significant interaction of pre-sleep metamemory and STAMP count [*F*(1,38.68) = 7.14, *p* = 0.011]. Given that the application of STAMPs occurred during SWOs, we considered if the effects involving the STAMP count for the “Tag & Cue” episodes were confounded by the number of concomitant SW events. To resolve this, we analyzed post-sleep metamemory for the “Sham” episodes using a linear mixed-effects model with subject as a random factor and with pre-sleep metamemory, the number of SW events, and the interaction between them as fixed effects. We did not find any significant effects (see Figure 5B). These results provide further evidence for the specific modulation of episodic memories that were both tagged during waking and cued during sleep.

For “Tag & Cue” episodes, subjects with weak pre-sleep metamemory who received more than the mean dose of STAMP cueing during sleep had a lower overnight increase in metamemory than those who received less than the mean dose. On the other hand, subjects with strong pre-sleep metamemory who received more than the mean dose of STAMP cueing had less of an overnight decrease in metamemory compared to those who received less than the mean dose (see Figure 5A). It is worth noting that the overall boost in metamemory for “Tag & Cue” episodes on Day 3 (see Figures 4A,B) occurred despite the presence of this significant interaction between pre-sleep metamemory and the number of STAMP applications on post-sleep metamemory, suggesting that the effect size can be further enhanced by regulating the number of STAMP applications based on pre-sleep metamemory.

### Sleep Biomarkers

Finally, we investigated the neurophysiological effects of “Tag & Cue” STAMPs during Night 2 owing to significant differences between “Tag & Cue” episode and other intervention types on Day 3. First, we contrasted average post-SW event changes in spectral power between “Active” and “Sham” Night 2 using non-parametric cluster-based permutation tests for five frequency bands (SW: 0.5–1.2 Hz, delta: 1–4 Hz, theta: 4–8 Hz, slow-spindle: 8–12 Hz, fast-spindle: 12–15 Hz) of relevance to memory consolidation (Mölle et al., 2011; Cox et al., 2014). Note high-amplitude stimulation artifacts preclude the inspection of EEG data during the application of STAMPs and up to 3 s following their offset. Significant “contrast clusters” of channel × time bins that differed in spectral power changes between “Active” and “Sham” Night 2 within 3–10 s following the offset of SW events were determined for each frequency band using a non-parametric permutation test to correct for multiple comparisons in the channel × time space (Maris and Oostenveld, 2007). A similar non-parametric cluster-based permutation test was then carried out for correlations of differences in overnight changes in metamemory between “Tag & Cue” and “Sham” episodes with the corresponding differences in spectral power changes following SW events to obtain “correlation clusters.” This analysis was restricted to the channel × time bins in the “contrast clusters” in order to relate overnight metamemory changes with specific spectral power modulations induced by STAMPs.

**FIGURE 6:**
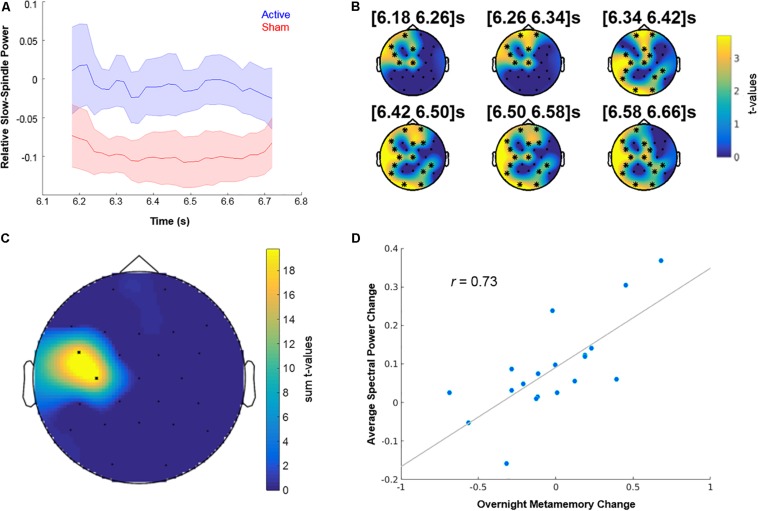
Neurophysiological and behavioral effects of STAMPs. **(A)** Dynamics of spectral power changes for the slow-spindle (8–12 Hz) “contrast cluster,” relative to the offset of SW events, showing significant differences between “Active” and “Sham” Night 2. **(B)** Scalp topography of *t*-values for the slow-spindle “contrast cluster” (clusterwise *p* = 0.025, additionally corrected for five frequency band comparisons) at six time points within the significant window (6.18–6.7 s following the offset of SW events). EEG channels participating in the cluster are marked by asterisks. **(C)** Scalp topography of summed *t*-values for the slow-spindle “correlation cluster” (clusterwise *p* = 0.013) from significant time bins. **(D)** Correlation of the differences in average spectral power change between “Active” and “Sham” Night 2 from “correlation cluster” bins and the differences in overnight metamemory change between “Tag & Cue” and “Sham” episodes (*r* = 0.73). Dots correspond to data from individual subjects (*N* = 19).

The analysis revealed a “contrast cluster” only in the slow-spindle band (8–12 Hz), such that the average post-SW event change in spectral power was lower (and negative) in the “Sham” Night 2 compared to the “Active” Night 2 (see Figure 6A and Supplementary Figure S2). This “contrast cluster” temporally extended from 6.18 to 6.7 s relative to the offset of SW events, and had a clusterwise *P*-value of 0.025 after the additional Bonferroni correction for the five frequency bands (*p* = 0.005, uncorrected). Scalp topography of *t*-values for the spectral power changes in the cluster indicated an early distribution over pre-frontal, left frontal, and left temporal areas, which then widened to include occipital and parietal regions (see Figure 6B and Supplementary Figure S2). These areas are consistent with the known involvement of dorsolateral prefrontal and parietal cortices (Chua et al., 2009) in the neural mechanisms of metamemory. Next, we correlated the differences in overnight metamemory changes between “Tag & Cue” and “Sham” episodes with the differences in average spectral power change within this “contrast cluster” between “Active” and “Sham” Night 2. We found a positive “correlation cluster” within the slow-spindle band between 6.56 and 6.64 s relative to the offset of SW events, with a clusterwise *P*-value of 0.013 (see Figure 6C), such that there was a positive correlation between the average STAMP-induced increase in slow-spindle power and the overnight improvement in metamemory (*r* = 0.73; see Figure 6D). Scalp topography of the summed *t*-values for the spectral power changes in this “correlation cluster” indicated a concentration on the left temporal region.

## Discussion

Applied neuroscience aims to develop technologies to affect behavior by modulating brain activity at the right spatiotemporal scale. It has been suggested that an intervention to enhance specific episodic memories needs to operate with high spatiotemporal resolution in the hippocampus (Suthana and Fried, 2014; Hampson et al., 2018). Contrary to this conventional wisdom, we have demonstrated a novel form of tES in healthy humans to boost the metamemory of specific episodes that were viewed only once in immersive VR. In particular, we discovered that unique spatiotemporal patterns (namely, STAMPs) of tES can be used for one-shot tagging of naturalistic episodes during waking and subsequent cueing during SWOs in sleep, and that those STAMPs when re-applied during SWOs can not only boost but also impair the metamemory of individual episodes depending on an interaction between pre-sleep metamemory and the number of STAMP applications through the night. Moreover, we found that post-stimulation increases in slow-spindle (8–12 Hz) power for left temporal areas in scalp EEG during sleep serve as a STAMP-induced biomarker of overnight metamemory improvements.

Spatiotemporal amplitude-modulated patterns did not modulate either the sleep architecture or the sleep efficiency of the subjects. In particular, there was no difference in the amount of time they were awake during the nights between the “Active” and “Sham” stimulation conditions. We therefore can conclude that subjects were able to sleep normally, without any disruption, despite the application of STAMPs. Moreover, we found that cueing with “Tag & Cue” STAMPs during sleep did not boost the long-term metamemory of “Tag & No Cue” episodes. This implies that the mere application of STAMP cues during SWOs does not have a non-specific effect on the metamemory of all recently viewed episodes.

There was a significant effect of stimulation condition on sensations during episode viewing with higher sensation ratings for the “Active” stimulation condition. This does not necessarily indicate a failure in blinding the subjects to the stimulation conditions. Even though the “Sham First” subjects were able to guess their stimulation conditions at a trend level, we did not find an effect of stimulation condition order on sensation ratings. Further, the significant difference in the metamemory of “Tag & Cue” and “Tag & No Cue” episodes on Day 3 within the “Active” stimulation condition cannot be accounted for by the potential confound of blinding. Nonetheless, blinding is an important concern in within-subjects designs, and in future studies we would suggest adding an additional control condition that employs one STAMP to tag an episode during waking and a different STAMP to cue that episode during sleep.

The STAMPs for this study were designed to induce different electric fields in different brain regions. Similar to sensory cue-based TMR studies (e.g., Rasch et al., 2007; Rudoy et al., 2009; Antony et al., 2012), our paradigm is based on the co-occurrence of STAMP-induced electric fields (and consequent neural effects) in various brain regions and the distributed neural activity evoked by the encoding of an episode. Previous TMR studies can be argued to take advantage of existing memory pathways from the pertinent sensory cortices to the medial temporal regions. Because STAMPs induce a wide range of effects in distributed regions, it can be argued that STAMPs may be stimulating these exact pathways along with other unknown pathways that can influence memory processes. We intentionally make no claims about the specific pathways that are stimulated, but rather suggest that the mechanism is related to the distributed pattern across many potential pathways. Further work is necessary to understand the degree to which any particular STAMP influences memory performance and the mechanisms related to those STAMPs.

We postulate that one-shot tagging is feasible owing to what appears to be the rapid associations that are formed between the STAMP-induced neural effects and the distributed memory traces. Just as in sensory cue-based TMR studies, these functional associations are then subsequently leveraged to potentially cue the reactivation of the memory traces during offline periods using the STAMPs alone for improved likelihood of consolidation. Memory traces are understood to be re-generated (or replayed) in response to partial or associated cues due to the pattern completion property of dense recurrent networks in the hippocampus. It is important to note that we are not employing STAMPs to selectively enhance the distributed neural activity of a memory trace during encoding as such. Indeed, in our experiment, the pairing between the 14 STAMPs and episodes in the “Active” stimulation condition was arbitrary and randomly chosen for each subject.

Absolute accuracy is a function of both memory sensitivity and response bias and is hence not considered a good metric for episodic memory recall. We found that STAMPs did not modulate the absolute accuracy of memory recall, but we are not able to draw any conclusions regarding whether STAMPs can instead modulate more sensitive bias-free metrics of episodic memory such as those that use Type-1 ROC curve analysis of confidence ratings on a scale from “Definitely False” to “Definitely True” for the declarative statements about the episodes (MacMillan and Creelman, 2005; Mickes et al., 2012). Our experimental design only supports Type-2 ROC curve analysis (which measures metamemory) owing to the two-level responses – one for whether a declarative statement was “True” or “False,” and the other to rate the correctness of the response on a scale from “Least Confident” to “Most Confident.” Though pseudo confidence rating distributions for Type-1 true positives and false positives can be theoretically constructed by spanning the range from “Definitely False” to “Definitely True” using highest confidence “False” and “True” responses, respectively (Gombos et al., 2012), the subjects’ Type-1 responses can only be predicted (with certain assumptions) rather than empirically measured (Galvin et al., 2003).

While it remains an open question if STAMPs also modulate episodic memory in a targeted manner, previous studies have suggested that memory and metamemory are likely correlated (Sacher et al., 2009; Yacoby et al., 2015). Broadly speaking, it has been proposed that metamemory is driven by either the familiarity of the recall cue, or the accessibility of any available pertinent information including that retrieved from memory in response to the cue (Koriat and Levy-Sadot, 2001). In either case, judgments about memories likely leverage the same neural representations of memory traces as the memory recall itself (Fleming and Dolan, 2012). The interaction between pre-sleep metamemory and STAMP count during sleep for “Tag & Cue” episodes on post-sleep metamemory can be understood with the framework of complementary learning systems (McClelland et al., 1995). According to this theory, episodic experiences are rapidly encoded in the hippocampus during waking for the short term. Before the hippocampal memory traces fade out, episodic memories are consolidated into long-term storage in the slow-learning neocortex during sleep through replays of pertinent neural activity patterns. The lack of an effect on metamemory on Day 2 could be due to the presence of sufficiently strong memory traces in the hippocampus for the episodes across the three intervention types, which will likely fade out by Day 3. Subjects with weak metamemory prior to sleep benefit from STAMP-based cueing because the sequential structure of the episodes can be strengthened in the hippocampus as well as consolidated in the cortex. We speculate that an excessive number of STAMPs can, however, roll back this benefit by learning remote, higher order links between events within the episodes. Subjects with strong metamemory prior to sleep do not benefit from STAMP cueing, on average, due to the same reason. So, the prescription for boosting the metamemory of episodic experiences is to apply an optimal number of STAMPs during sleep for subjects with weaker pre-sleep metamemory, and to not intervene for subjects with stronger pre-sleep metamemory (cf., Schapiro et al., 2018).

Our study has many potential extensions. First, we need to ascertain if STAMPs can also boost the memory of targeted episodic experiences by measuring Type-1 confidence ratings, which can be used to compute both memory and metamemory using ROC curve analyses (Galvin et al., 2003). Second, the neural effects of STAMPs in scalp EEG underlying the one-shot tagging of episodes during wake need to be investigated (similar to the analyses of sleep biomarkers). Third, we need to validate whether STAMPs can indeed tag distributed neural representations in a single trial by altering the functional connectivity as well as the spike timing within the brain in unique ways. This can be done using simultaneous multi-site intracranial recordings of local field potential, multi-unit activity, and single-neuron activity during wake and sleep in non-human primates and clinical human populations (cf., Krause et al., 2017, 2019b). Fourth, we need to assess the longevity of preservation of episodic metamemory with a longer longitudinal study and whether there would be any adverse effects in other aspects of behavior. Fifth, the library of mutually orthogonal STAMPs can be personalized for each individual user’s head as well as optimized for particular brain areas that are pertinent to a given task to further boost the efficacy of STAMPs to tag and cue memories. Sixth, new sleep studies are in order that aim to maximize the benefits of STAMPs during sleep by regulating the number of STAMP applications based on pre-sleep metamemory (see Figure 5), as well as the intensity and frequency of particular STAMPs based on post-stimulation biomarkers of metamemory (see Figure 6). Seventh, the application of STAMPs was limited to the presence of SWOs during sleep, and so the optimal dose may not be deliverable to a subject due to impoverished SW activity. A potential strategy is to employ SW tACS to boost SWOs (Jones et al., 2018; Ketz et al., 2018) in alternating blocks with STAMPs. However, our recent attempt at this strategy failed to boost the consolidation of individual sequential experiences (Lerner et al., 2019). Potential reasons for the failure include a much smaller sample size for a between-subjects design (*N* = 12), the usage of a hybrid task with both procedural and declarative memory elements, the presence of a hidden temporal regularity shared across all sequence types, and mutual interference between specific STAMPs and non-specific SW tACS. Finally, we can perform a control experiment to rule out the potential but unlikely possibility that the behavioral effects of tES STAMPs are mediated indirectly via tactile sensations on the scalp, instead of directly through electric fields induced in the brain volume. In this regard, the spatial and frequency specificity of tACS to entrain single neurons in the hippocampus of awake macaque monkeys (Krause et al., 2019b; see also Johnson et al., 2019) has been shown to be preserved even when the somatosensory afferents in the scalp are blocked using topical anesthesia (Vieira et al., 2019; see also Krause et al., 2019a).

The effect of STAMPs on overnight metamemory improvements was correlated with an increase in slow-spindle (8–12 Hz) power for left temporal areas following STAMPs during sleep. Spindles are demonstrably a critical component of sleep-dependent memory consolidation (Fogel and Smith, 2011) as shown in rodents (Eschenko et al., 2006) as well as humans (Cox et al., 2012). Recent optogenetic work in rats demonstrated a causal role for sleep spindles in coupling SWOs and hippocampal sharp wave ripples for effective consolidation to long-term memory storage (Latchoumane et al., 2017). Spindles reverberate in circular wave-like patterns across temporal, parietal, and frontal regions repeatedly throughout the night, regulating the process of memory re-organization over time and space (Muller et al., 2016). To the best of our knowledge, there have been no studies on the hemispheric differences in the sleep-dependent consolidation of episodic memory and/or metamemory. In this regard, our results suggest that the left temporal lobe may play a key role in boosting the consolidation of episodic metamemory. However, given the limited spatial resolution of EEG measurements, further work will be necessary to localize the specific neural sources contributing to the observed left temporal slow-spindle cluster. Also, volume conduction effects may influence the non-parametric cluster analysis, but the only impact would be on the spatial specificity and not the false positive rate of the presence of an effect. We therefore cannot make any conclusive statements on the cortical source of the observed effects.

## Conclusion

In summary, we have developed a novel non-sensory non-invasive method to tag naturalistic episodic experiences in one shot and cue them during offline periods to boost their metamemory in a targeted manner. We overcome the limitations of previous TMR studies (e.g., Rasch et al., 2007; Rudoy et al., 2009; Antony et al., 2012) by demonstrating one-shot tagging during wake and cueing during sleep with tES STAMPs, which are not only scalable but also do not suffer interference from ambient environmental cues in real-world settings. Our results suggest that, unlike relatively localized brain circuits responsible for regulating mood (Rao et al., 2018) and movement (Follett et al., 2010), episodic memories are processed by a much more widespread network of brain areas. We believe that our study will pave the way for next-generation transcranial brain–machine interfaces that can boost learning and memory in healthy humans for real-world tasks. Such a non-invasive approach can also potentially benefit a majority of patients with learning and memory deficits at much lower cost and risk than required for implanting intracranial electrode arrays. It could also be possible to enhance the efficacy of exposure behavioral therapy with immersive VR using STAMP-based tagging and cueing for the treatment of post-traumatic stress disorder.

## Data Availability Statement

The datasets generated for this study are available on request to the corresponding author.

## Ethics Statement

This study involving human subjects was reviewed and approved by the Chesapeake Institutional Review Board. The subjects provided their written informed consent to participate in this study.

## Author Contributions

PP and MH designed the research. PP, SS, NK, SR, IL, AJ, BR, NB, AH, TM, JC, VC, and MH performed the research. PP, RH, NK, AJ, BR, and TM analyzed the data. PP wrote the manuscript.

## Conflict of Interest

PP, SS, RH, NK, SR, JC, and MH were employed by HRL Laboratories, LLC. PP, JC, and MH have a patent on using tES for targeted memory reactivation (US Patent No. 10,307,592). The remaining authors declare that the research was conducted in the absence of any commercial or financial relationships that could be construed as a potential conflict of interest.

## References

[B1] Antony J. W. Gobel E. W. O’Hare J. K. Reber P. J. Paller K. A. (2012). Cued memory reactivation during sleep influences skill learning. *Nat. Neurosci.* 15:1114. 10.1038/nn.3152 22751035 PMC3498459

[B2] Bendor D. Wilson M. A. (2012). Biasing the content of hippocampal replay during sleep. *Nat. Neurosci.* 15 1439–1444. 10.1038/nn.3203 22941111 PMC4354843

[B3] Berry R. B. Brooks R. Gamaldo C. E. Harding S. M. Marcus C. L. Vaughn B. V. (2012). *The AASM Manual for the Scoring of Sleep and Associated Events. Rules, Terminology and Technical Specifications, Version 2.0.* Darien, IL: American Academy of Sleep Medicine.

[B4] Buzsáki G. (1996). The hippocampo-neocortical dialogue. *Cereb. Cortex* 6 81–92. 10.1093/cercor/6.2.81 8670641

[B5] Chua E. F. Schacter D. L. Sperling R. A. (2009). Neural correlates of metamemory: a comparison of feeling-of-knowing and retrospective confidence judgments. *J. Cogn. Neurosci.* 21 1751–1765. 10.1162/jocn.2009.21123 18823230 PMC2709699

[B6] Ciuparu A. Mureşan R. C. (2016). Sources of bias in single-trial normalization procedures. *Eur. J. Neurosci.* 43 861–869. 10.1111/ejn.13179 26797876

[B7] Cox R. Hofman W. F. Talamini L. M. (2012). Involvement of spindles in memory consolidation is slow wave sleep-specific. *Learn Mem.* 19 264–267. 10.1101/lm.026252.112 22700468

[B8] Cox R. Korjoukov I. de Boer M. Talamini L. M. (2014). Sound asleep: processing and retention of slow oscillation phase-targeted stimuli. *PLoS One* 9:e101567. 10.1371/journal.pone.0101567 24999803 PMC4084884

[B9] Crowley R. Javadi A. (2019). The Modulatory Effect of Oscillatory Reinstatement During Slow-wave Sleep on declarative Memory Consolidation. *OSF Preprints.* 10.31219/osf.io/8yxge.

[B10] Delorme A. Makeig S. (2004). EEGLab: an open source toolbox for analysis of single-trial EEG dynamics including independent component analysis. *J. Neurosci. Methods* 134 9–21. 10.1016/j.jneumeth.2003.10.009 15102499

[B11] Eschenko O. Mölle M. Born J. Sara S. J. (2006). Elevated sleep spindle density after learning or after retrieval in rats. *J. Neurosci.* 26 12914–12920. 10.1523/jneurosci.3175-06.2006 17167082 PMC6674950

[B12] Fleming S. M. Dolan R. J. (2012). The neural basis of metacognitive ability. *Philos. Trans. R. Soc. B* 367 1338–1349. 10.1098/rstb.2011.0417 22492751 PMC3318765

[B13] Fleming S. M. Lau H. C. (2014). How to measure metacognition. *Front. Hum. Neurosci.* 8:443. 10.3389/fnhum.2014.00443 25076880 PMC4097944

[B14] Fogel S. M. Smith C. T. (2011). The function of the sleep spindle: a physiological index of intelligence and a mechanism for sleep-dependent memory consolidation. *Neurosci. Biobehav. R* 35 1154–1165. 10.1016/j.neubiorev.2010.12.003 21167865

[B15] Follett K. A. Weaver F. M. Stern M. Hur K. Harris C. L. Luo P. (2010). Pallidal versus subthalamic deep-brain stimulation for Parkinson’s disease. *New Engl. J. Med.* 362 2077–2091. 10.1056/NEJMoa0907083 20519680

[B16] Galvin S. J. Podd J. V. Drga V. Whitmore J. (2003). Type 2 tasks in the theory of signal detectability: discrimination between correct and incorrect decisions. *Psychon. Bull. Rev.* 10 843–876. 10.3758/bf03196546 15000533

[B17] Gombos V. Pezdek K. Haymond K. (2012). Forced confabulation affects memory sensitivity as well as response bias. *Mem. Cogn.* 40 127–134. 10.3758/s13421-011-0129-5 21786086

[B18] Hampson R. E. Song D. Robinson B. S. Fetterhoff D. Dakos A. S. Roeder B. M. (2018). Developing a hippocampal neural prosthetic to facilitate human memory encoding and recall. *J. Neural Eng.* 15:036014. 10.1088/1741-2552/aaaed7 29589592 PMC6576290

[B19] Javadi A.-H. Glen J. C. Halkiopoulos S. Schulz M. Spiers H. J. (2017). Oscillatory reinstatement enhances declarative memory. *J. Neurosci.* 37 9939–9944. 10.1523/jneurosci.0265-17.2017 28912159 PMC5637119

[B20] Ji D. Wilson M. A. (2007). Coordinated memory replay in the visual cortex and hippocampus during sleep. *Nat. Neurosci.* 10 100–107. 10.1038/nn1825 17173043

[B21] Johnson L. Alekseichuk I. Krieg J. Doyle A. Yu Y. Vitek J. (2019). Dose-dependent effects of transcranial alternating current stimulation on spike timing in awake nonhuman primates. *bioRxiv [Preprint].* 10.1126/sciadv.aaz2747 PMC7467690 32917605

[B22] Jones A. P. Choe J. Bryant N. B. Robinson C. S. H. Ketz N. A. Skorheim S. W. (2018). Dose-dependent effects of closed-loop tACS delivered during slow-wave oscillations on memory consolidation. *Front. Neurosci.* 12:867. 10.3389/fnins.2018.00867 30538617 PMC6277682

[B23] Ketz N. A. Jones A. P. Bryant N. B. Clark V. P. Pilly P. K. (2018). Closed-loop slow-wave tACS improves sleep dependent long-term memory generalization by modulating endogenous oscillations. *J. Neurosci.* 38 7314–7326. 10.1523/JNEUROSCI.0273-18.2018 30037830 PMC6596034

[B24] Koriat A. Levy-Sadot R. (2001). The combined contributions of the cue-familiarity and accessibility heuristics to feelings of knowing. *J. Exp. Psychol. Learn. Mem. Cogn.* 27 34–53. 10.1037/0278-7393.27.1.34 11204106

[B25] Krause M. R. Vieira P. G. Csorba B. A. Pilly P. K. Pack C. C. (2019a). Reply to Khatoun et al.: speculation about brain stimulation must be constrained by observation. *Proc. Natl. Acad. Sci. U.S.A.* 116 22440–22441. 10.1073/pnas.1914483116 31615881 PMC6842577

[B26] Krause M. R. Vieira P. G. Csorba B. A. Pilly P. K. Pack C. C. (2019b). Transcranial alternating current stimulation entrains single-neuron activity in the primate brain. *Proc. Natl. Acad. Sci. U.S.A.* 116 5747–5755. 10.1073/pnas.1815958116 30833389 PMC6431188

[B27] Krause M. R. Zanos T. P. Csorba B. A. Pilly P. K. Choe J. Phillips M. E. (2017). Transcranial direct current stimulation facilitates associative learning and alters functional connectivity in the primate brain. *Curr. Biol.* 27 3086–3096. 10.1016/j.cub.2017.09.020 29033331

[B28] Latchoumane C. F. V. Ngo H. V. V. Born J. Shin H. S. (2017). Thalamic spindles promote memory formation during sleep through triple phase-locking of cortical, thalamic, and hippocampal rhythms. *Neuron* 95 424–435. 10.1016/j.neuron.2017.06.025 28689981

[B29] Lerner I. Ketz N. A. Jones A. P. Bryant N. B. Robert B. Skorheim S. W. (2019). Transcranial current stimulation during sleep facilitates insight into temporal rules, but does not consolidate memories of individual sequential experiences. *Sci. Rep.* 9:1516. 10.1038/s41598-018-36107-7 30728363 PMC6365565

[B30] Liao H. I. Wu D. A. Halelamien N. Shimojo S. (2013). Cortical stimulation consolidates and reactivates visual experience: neural plasticity from magnetic entrainment of visual activity. *Sci. Rep.* 3:2228. 10.1038/srep02228 23863977 PMC3714647

[B31] MacMillan N. A. Creelman C. D. (2005). *Detection Theory: A user’s Guide*, 2nd Edn Mahwah, NJ: Erlbaum Press.

[B32] Maris E. Oostenveld R. (2007). Nonparametric statistical testing of EEG-and MEG-data. *J. Neurosci. Methods* 164 177–190. 10.1016/j.jneumeth.2007.03.024 17517438

[B33] McClelland J. L. McNaughton B. L. O’Reilly R. C. (1995). Why there are complementary learning systems in the hippocampus and neocortex: insights from the successes and failures of connectionist models of learning and memory. *Psychol. Rev.* 102:419. 10.1037/0033-295X.102.3.419 7624455

[B34] Mickes L. Flowe H. D. Wixted J. T. (2012). Receiver operating characteristic analysis of eyewitness memory: comparing the diagnostic accuracy of simultaneous versus sequential lineups. *J. Exp. Psychol.* 18 361. 10.1037/a0030609 23294282

[B35] Mölle M. Bergmann T. O. Marshall L. Born J. (2011). Fast and slow spindles during the sleep slow oscillation: disparate coalescence and engagement in memory processing. *Sleep* 34 1411–1421. 10.5665/SLEEP.1290 21966073 PMC3174843

[B36] Muller L. Piantoni G. Koller D. Cash S. S. Halgren E. Sejnowski T. J. (2016). Rotating waves during human sleep spindles organize global patterns of activity that repeat precisely through the night. *eLife* 5:e17267. 10.7554/eLife.17267 27855061 PMC5114016

[B37] Nelson T. O. Narens L. (1990). Metamemory: a theoretical framework and new findings. *Psychol. Learn. Motiv.* 26 125–173. 10.1016/s0079-7421(08)60053-5

[B38] Oldfield R. C. (1971). The assessment and analysis of handedness: the Edinburgh inventory. *Neuropsychologia* 9 97–113. 10.1016/0028-3932(71)90067-4 5146491

[B39] Oostenveld R. Fries P. Maris E. Schoffelen J. M. (2011). FieldTrip: open source software for advanced analysis of MEG, EEG, and invasive electrophysiological data. *Comp. Intel. Neurosci.* 2011:1. 10.1155/2011/156869 21253357 PMC3021840

[B40] Rao V. R. Sellers K. K. Wallace D. L. Lee M. B. Bijanzadeh M. Sani O. G. (2018). Direct electrical stimulation of lateral orbitofrontal cortex acutely improves mood in individuals with symptoms of depression. *Curr. Biol.* 28 3893–3902. 10.1016/j.cub.2018.10.026 30503621

[B41] Rasch B. Born J. (2013). About sleep’s role in memory. *Physiol. Rev.* 93 681–766. 23589831 10.1152/physrev.00032.2012 PMC3768102

[B42] Rasch B. Buchel C. Gais S. Born J. (2007). Odor cues during slow-wave sleep prompt declarative memory consolidation. *Science* 315 1426–1429. 10.1126/science.1138581 17347444

[B43] Rothschild G. Eban E. Frank L. M. (2017). A cortical–hippocampal–cortical loop of information processing during memory consolidation. *Nat. Neurosci.* 20:251. 10.1038/nn.4457 27941790 PMC5783826

[B44] Rudoy J. D. Voss J. L. Westerberg C. E. Paller K. A. (2009). Strengthening individual memories by reactivating them during sleep. *Science* 326 1079–1079. 10.1126/science.1179013 19965421 PMC2990343

[B45] Sacher M. Taconnat L. Souchay C. Isingrini M. (2009). Divided attention at encoding: effect on feeling-of-knowing. *Conscious. Cogn.* 18 754–761. 10.1016/j.concog.2009.04.001 19423362

[B46] Schapiro A. C. McDevitt E. A. Rogers T. T. Mednick S. C. Norman K. A. (2018). Human hippocampal replay during rest prioritizes weakly learned information and predicts memory performance. *Nat. Commun.* 9:3920. 10.1038/s41467-018-06213-1 30254219 PMC6156217

[B47] Suthana N. Fried I. (2014). Deep brain stimulation for enhancement of learning and memory. *Neuroimage* 85 996–1002. 10.1016/j.neuroimage.2013.07.066 23921099 PMC4445933

[B48] Vieira P. G. Krause M. R. Pack C. C. (2019). tACS entrains neural activity while somatosensory input is blocked. *bioRxiv [Preprint].* 10.1371/journal.pbio.3000834 PMC7553316 33001971

[B49] Yacoby A. Dudai Y. Mendelsohn A. (2015). Metamemory ratings predict long-term changes in reactivated episodic memories. *Front. Behav. Neurosci.* 9:20. 10.3389/fnbeh.2015.00020 25709571 PMC4321599

