# Supplementary material for: One-Shot Tagging During Wake and Cueing During Sleep With Spatiotemporal Patterns of Transcranial Electrical Stimulation Can Boost Long-Term Metamemory of Individual Episodes in Humans
Source: Front Neurosci. 2020 Jan 10;13:1416. doi: 10.3389/fnins.2019.01416 (PMC6967741; doi:10.3389/fnins.2019.01416)
Supplement: Supplementary file 1 [file Data_Sheet_1.PDF]

Supplementary Material

1. Supplementary Figures

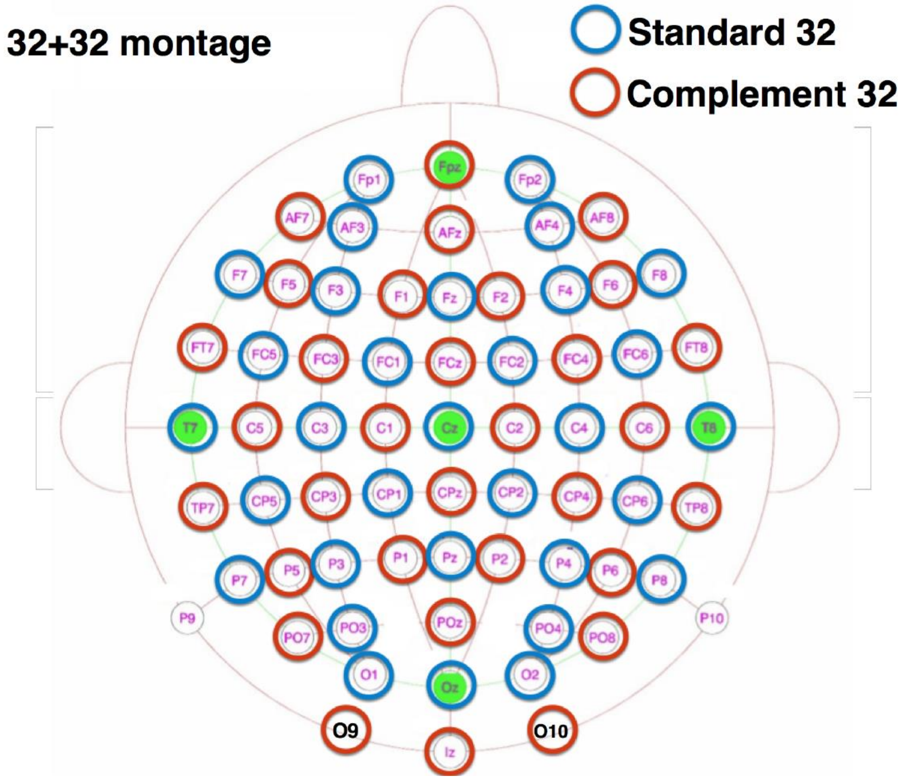

Figure S1. Scalp layout of the 32 EEG and 32 stimulation electrodes used in the waking and sleep interventions. The EEG electrodes were placed according to the international 10-20 system.

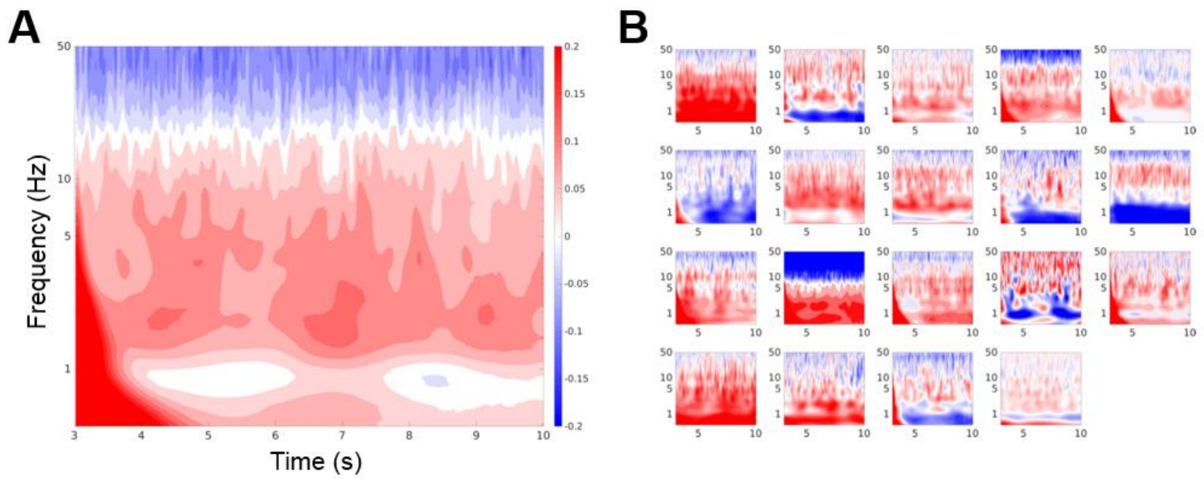

Figure S2. Time-frequency plots of the contrast between 'Active' and 'Sham' stimulation conditions in the STAMP-induced spectral power changes, averaged across channels and trials through Night 2. Post-stimulation time (s) is on the x-axis, frequency (Hz) is on the y-axis, and the color represents the contrast in the spectral power changes (Active – Sham). (A) Grand average across the subjects (N=19). (B) Time-frequency plots for individual subjects. Note that only the slow-spindle (8-12 Hz) 'contrast cluster' shown in Figure 6B was significant.

2. Supplementary Movies

Movie Captions
Movie S1. Video of the "Fire Response" episode used in the experiment.

Movie S2. Video demonstration of the GUI that was used to administer the memory recall tests.

3. Supplementary Table

Table S1. Names of the episodes in each of the four subgroups (color coded) that were used in the experiment.

Episode Group A	Episode Group B
Bomb Drop	Call The Car
Car Bomb Assassination	Car Bomb Attack
Cardiac Arrest	Domestic Incident
Deadly Argument	Drive By
Failed Rescue	Exposed Rendezvous
Handoff	Fire Response
Helicopter Scare	Missile Strike
Interrupted Meeting	Shopping
Missed the Bus	Sniper
No Escape	Street Hawker
Pick Pocket	Suicide Bomb
Repair Man	The Dare
Timely Exit	The Meeting
Waiting Gunman	The Serenade

4. Supplementary Note

Details for each of the 28 episodes that were used in the experiment

(1) Bomb Drop

Textual prompt: In the episode where two characters meet in front of the building, one enters the building, and an explosion occurs

Episode snapshot:

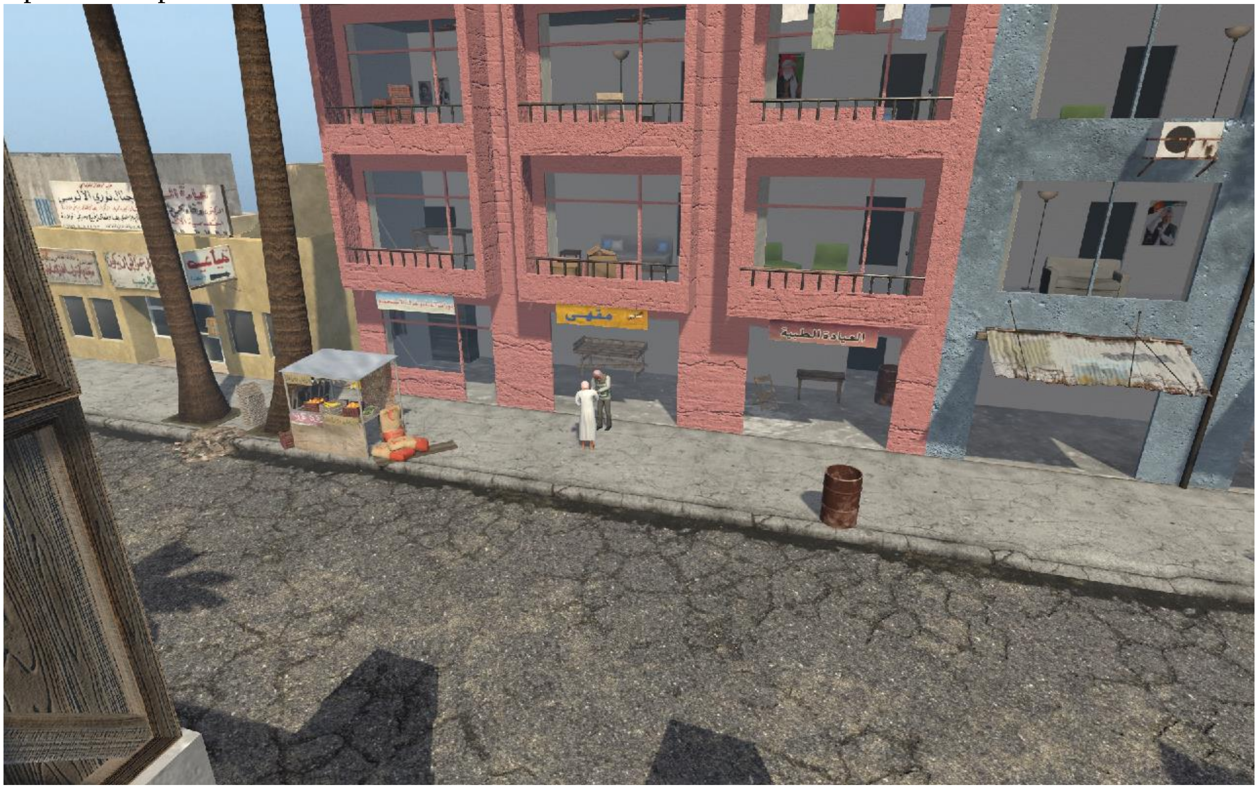

Pictures of characters:

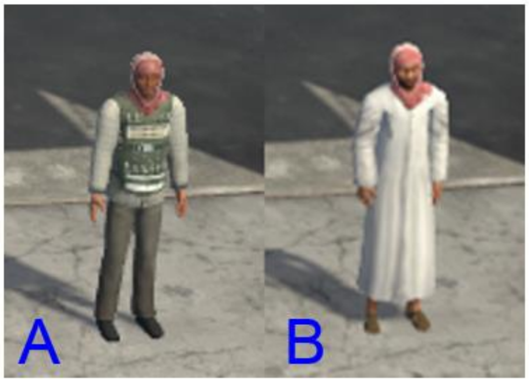

Question 1: The bomb exploded on the 2nd floor
Answer: False

Question 2: Character A gets out of a car
Answer: False

Question 3: Character A exits the building before an explosion occurs
Answer: True

Question 4: Character A fell down when the explosion occurred
Answer: False

Question 5: Character B runs away from the building (to the right) before the blast
Answer: True

Question 6: Character A was inside building when blast occurred
Answer: False

Question 7: Character B enters car and drives away
Answer: False

Question 8: Character A enters the building after interacting with Character B on the sidewalk
Answer: True

Question 9: Character B runs away from building before explosion occurs
Answer: True

Question 10: Neither Character A nor Character B came from the building
Answer: True

(2) Call The Car
Textual prompt: In the episode where one character arrives in a vehicle, converses with the other, then they both leave in the vehicle

Episode snapshot:
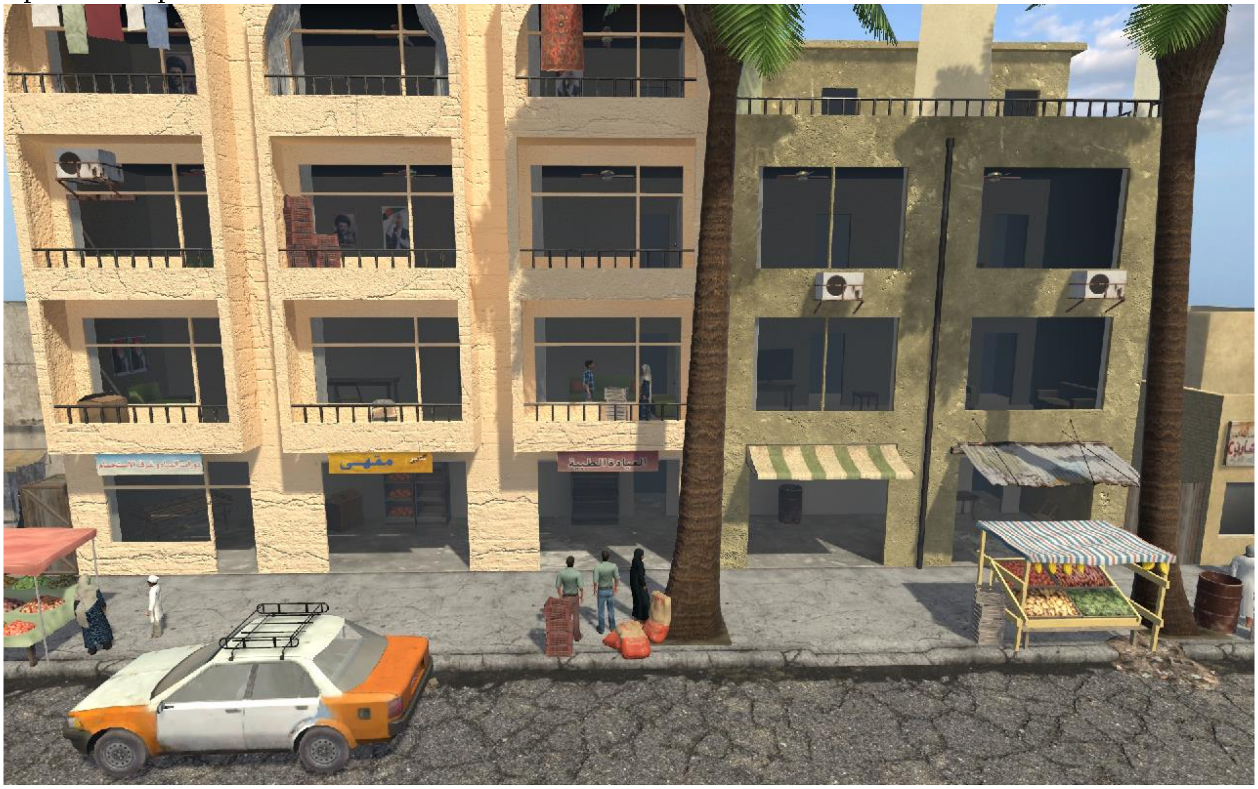

Pictures of characters:
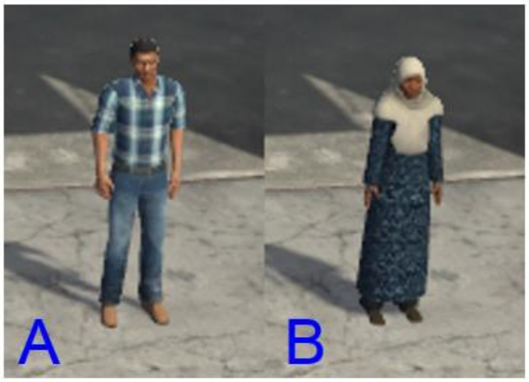

Question 1: Character B looked out a window before a car arrived
Answer: True

Question 2: Character A got out of a car and ran into the building
Answer: True

Question 3: The two characters met on the street
Answer: False

Question 4: Character B is only seen in a single room before being met by character A
Answer: True

Question 5: CharatersA and B converse a second time on the sidewalk
Answer: False

Question 6: Characters A and B converse in multiple rooms in the building
Answer: False

Question 7: Characters A and B left the building through the same exit
Answer: True

Question 8: A police car arrived
Answer: False

Question 9: A helicopter flew over while Characters A and B were conversing
Answer: False

Question 10: Character B followed Character A to the car
Answer: True

(3) Car Bomb Assassination
Textual prompt: In the episode where several characters converse inside the building, exit the building, and the police car explodes after some get into the vehicle

Episode snapshot:

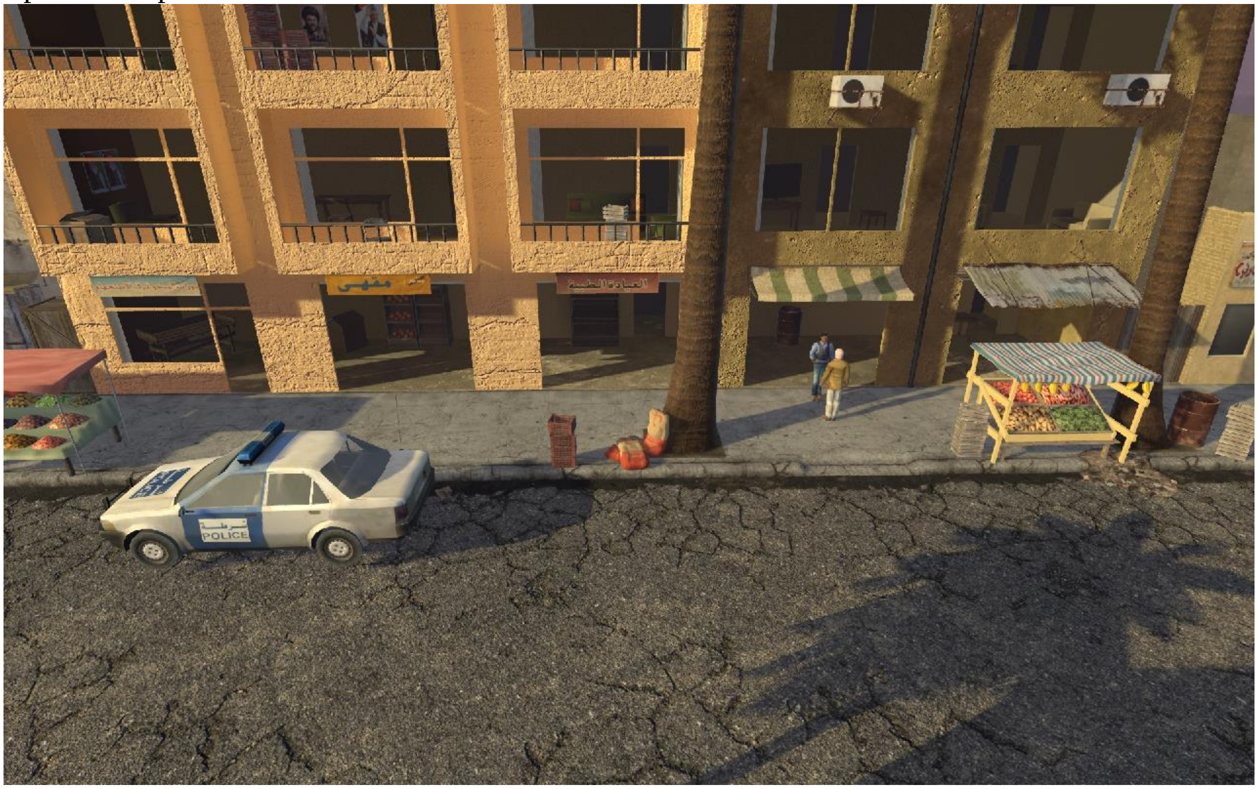

Pictures of characters:

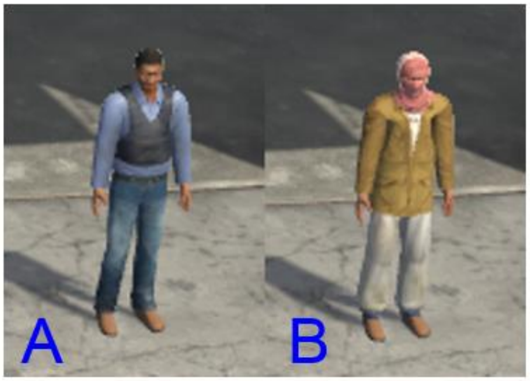

Question 1: The characters met inside the building
Answer: True

Question 2: The characters left the building and went in different directions
Answer: False

Question 3: A person was seen running from the car before it exploded
Answer: False

Question 4: The car burned after it blew up
Answer: True

Question 5: There was only one vehicle in the scene
Answer: False

Question 6: An ambulance pulled up after the vehicle exploded
Answer: True

Question 7: Character A and B exit the building together
Answer: True

Question 8: The car started smoking shortly before exploding
Answer: False

Question 9: Character A runs down the street after the explosion
Answer: False

Question 10: Gunshots are heard before explosion
Answer: False

(4) Car Bomb Attack

Textual prompt: In the episode where a car arrives, one of the characters exits the car, and the car explodes

Episode snapshot:

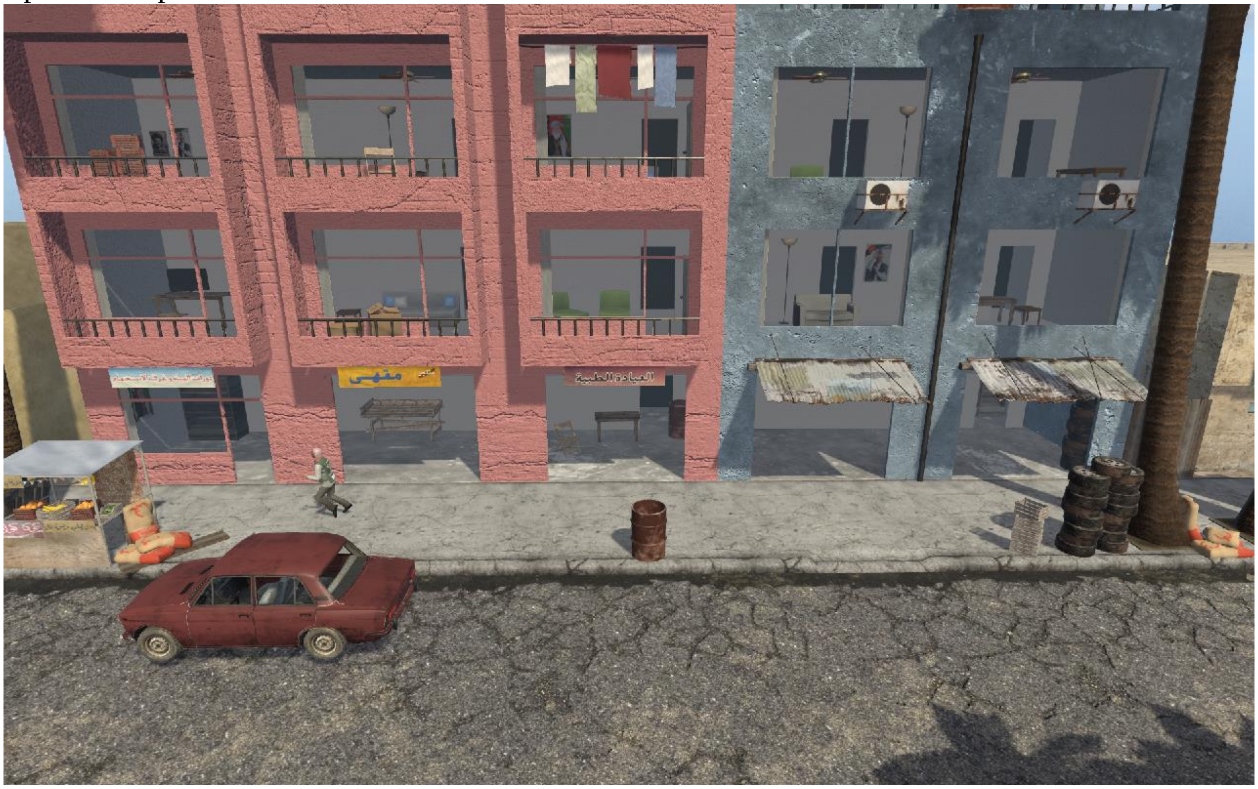

Pictures of characters:

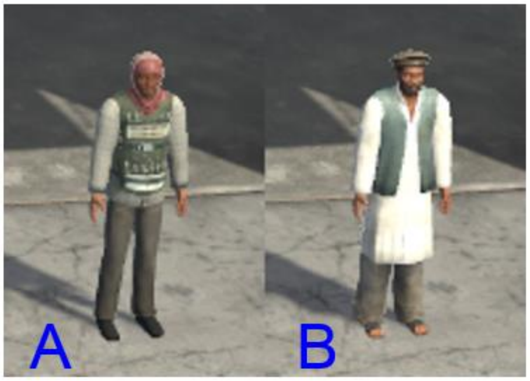

Question 1: Character B entered the building just before a car arrived
Answer: True

Question 2: Character A ran out of the car and into the building
Answer: False

Question 3: Character B left the building before the car blew up
Answer: False

Question 4: Characters A and B conversed before the explosion
Answer: False

Question 5: Character B ran away from the building after the explosion
Answer: True

Question 6: Character A falls down on street when explosion occurs
Answer: False

Question 7: Character A arrives in a police vehicle
Answer: False

Question 8: Fire starts in the building
Answer: True

Question 9: Character A does not run into the building before the car explodes
Answer: True

Question 10: The car catches fire after the explosion
Answer: True

(5) Cardiac Arrest
Textual prompt: In the episode where one character falls over on the sidewalk, another goes inside, and an ambulance arrives

Episode snapshot:
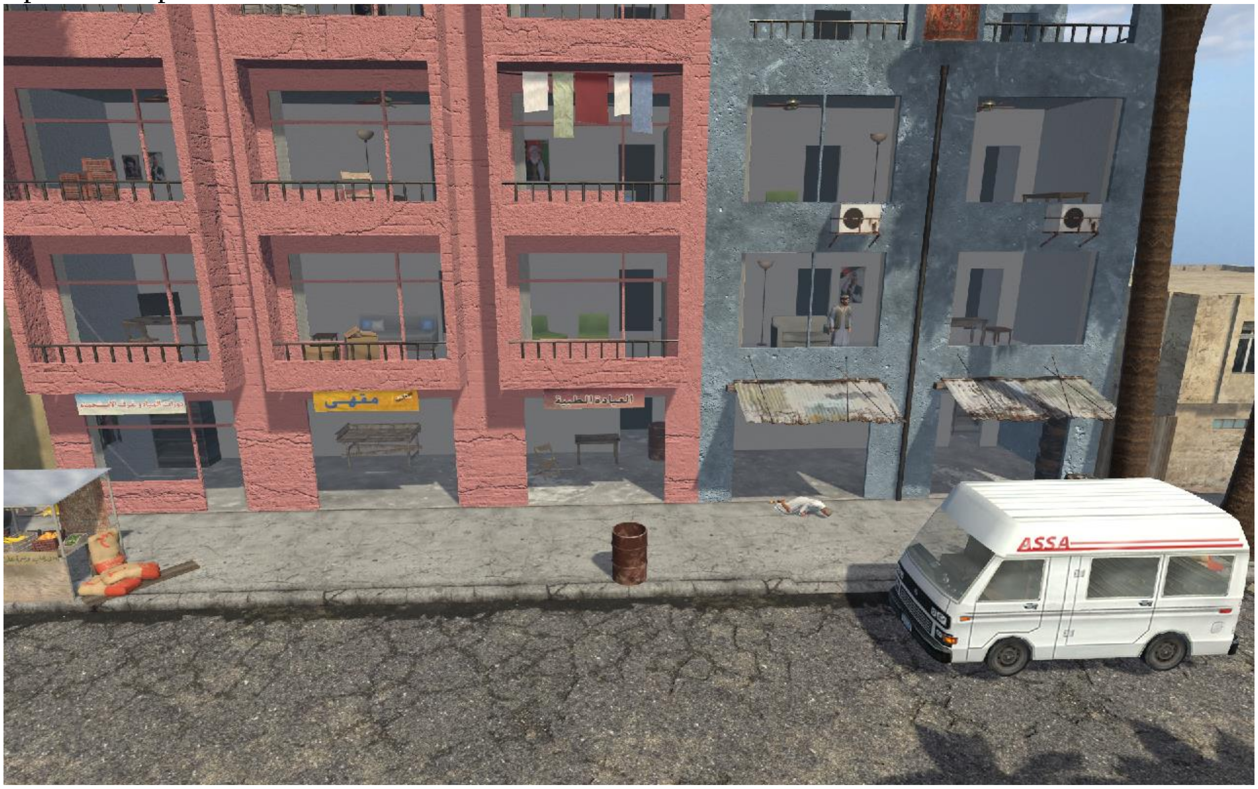

Pictures of characters:
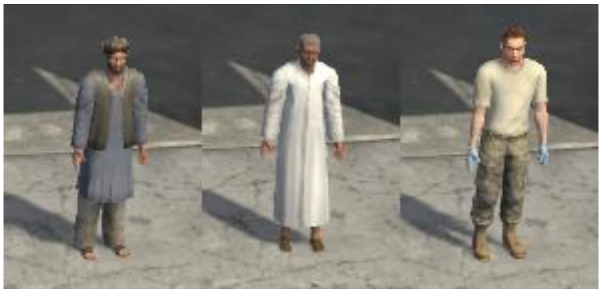

Question 1: Character B is loaded into the ambulance
Answer: False

Question 2: Character B stands back up after the ambulance arrives
Answer: False

Question 3: Gunshots are heard before Character B falls down
Answer: False

Question 4: Character C arrives before the ambulance
Answer: False

Question 5: Characters A and B exit the building start talking on the sidewalk
Answer: True

Question 6: Character A does not come back downstairs after the ambulance arrives
Answer: True

Question 7: A police car arrives
Answer: False

Question 8: Character C gets out of an ambulance to help Character B
Answer: True

Question 9: After the ambulance arrives, Character A leaves the building and runs down the sidewalk
Answer: False

Question 10: Character A closes the blinds after the ambulance arrives
Answer: False

(6) Deadly Argument

Textual prompt: In the episode where two characters have a disagreement and one shoots the other in the building

Episode snapshot:

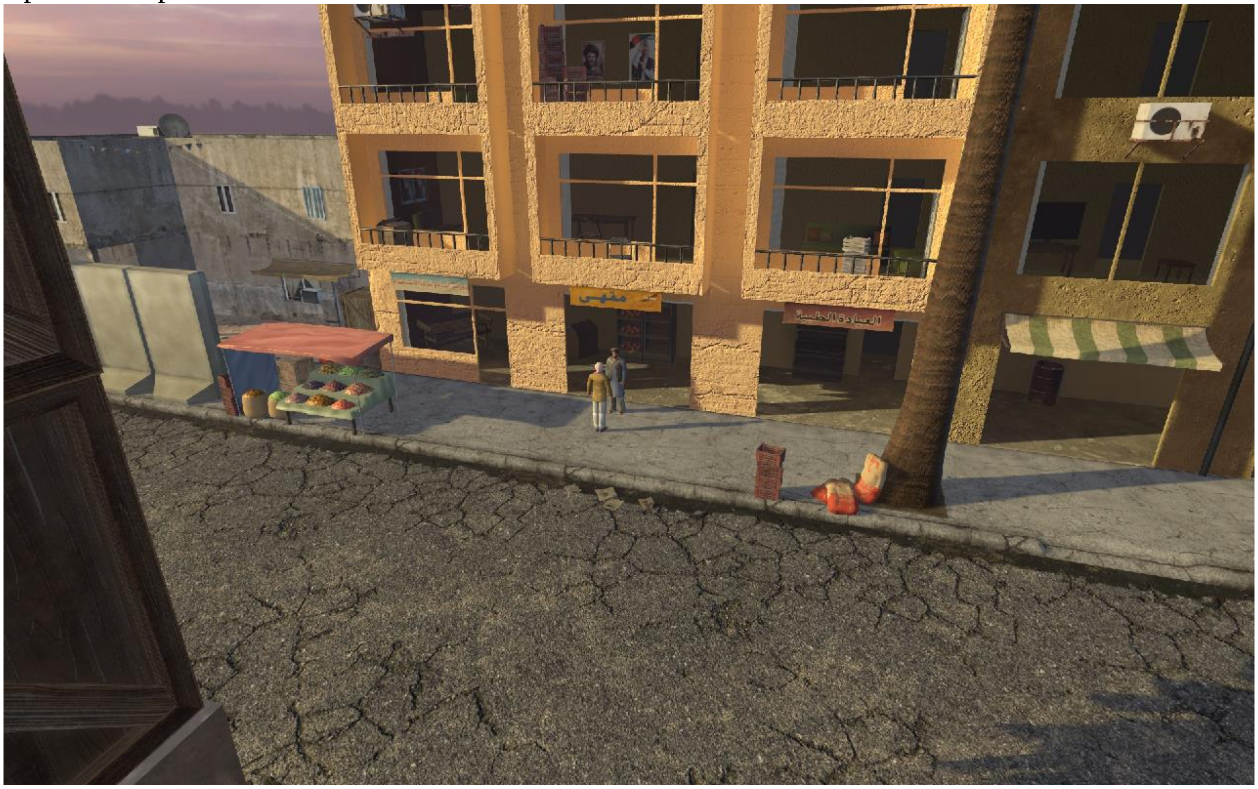

Pictures of characters:

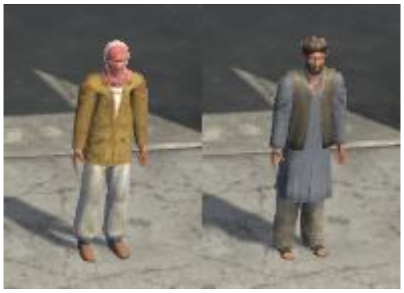

Question 1: Characters A and B walked into view together
Answer: False

Question 2: Character B entered the building while Character A walked away from him
Answer: True

Question 3: Character A enters the building with Character B
Answer: False

Question 4: Characters A and B converse again inside the building
Answer: False

Question 5: Character A ran from the building after shooting Character B
Answer: True

Question 6: Characters A and B entered the building together
Answer: True

Question 7: After Character B fell to the floor, Character A brought him outside the building
Answer: False

Question 8: Characters A and B were seen together in a room in the middle of the building
Answer: True

Question 9: Character A shot Character B from a different room
Answer: False

Question 10: An ambulance drove up to the front of the building
Answer: False

(7) Domestic Incident

Textual prompt: In the episode where two characters have a disagreement and one shoots the other inside the building

Episode snapshot:

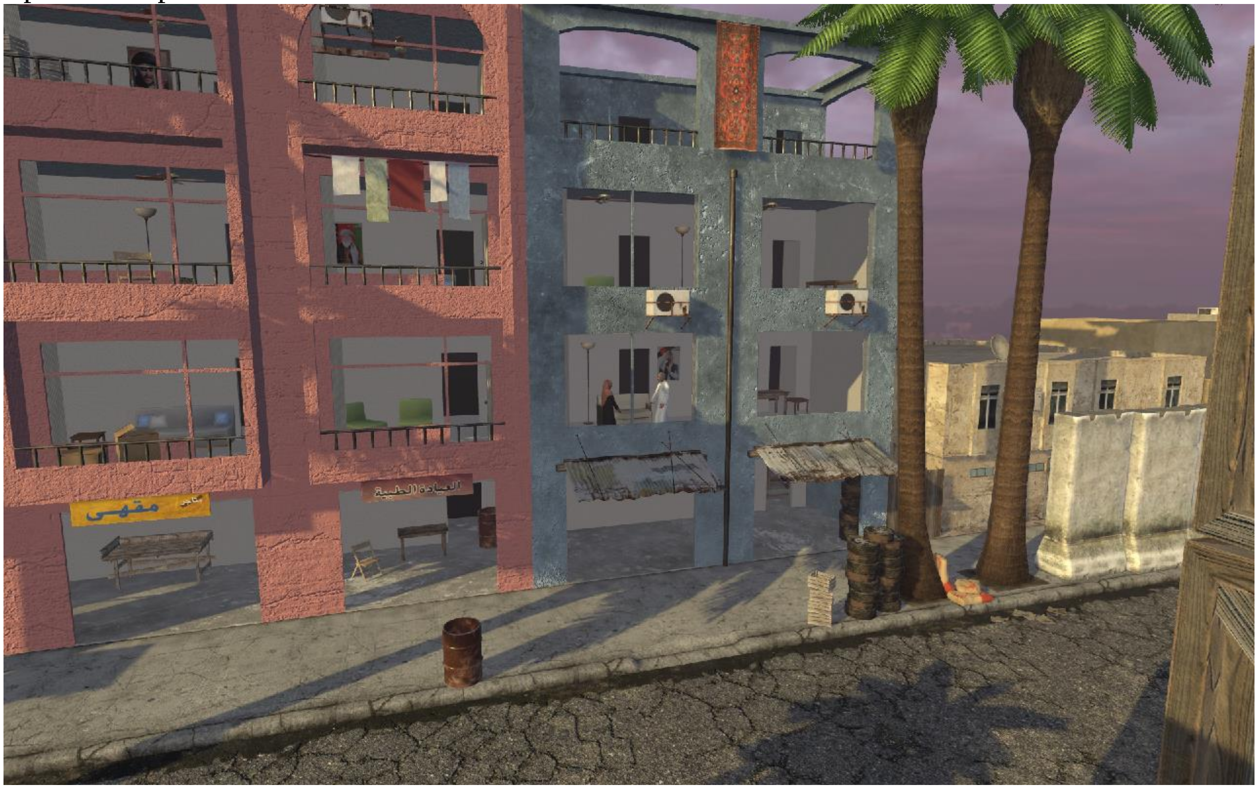

Pictures of characters:

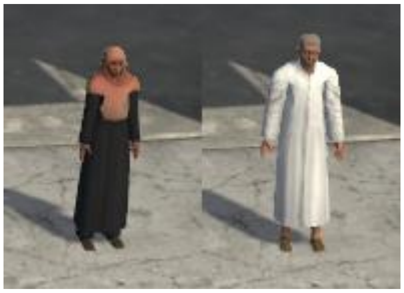

Question 1: Character A stays in the room she is in while Character B goes into another room
Answer: True

Question 2: Character B speaks to Character A in a room on the second floor
Answer: True

Question 3: A police car pulled up while Characters A and B were in separate rooms
Answer: False

Question 4: Character B left the room and then came back
Answer: True

Question 5: Character A shot Character B
Answer: False

Question 6: Character B runs from the building after the shooting
Answer: True

Question 7: Character B fires many gun shots
Answer: False

Question 8: Character A runs down the street away from the building
Answer: False

Question 9: Character B arrives by car
Answer: False

Question 10: Character B shoots Character A from behind
Answer: True

(8) Drive By

Textual prompt: In the episode where two characters are in front of the building as a vehicle drives past and gunshots are heard

Episode snapshot:

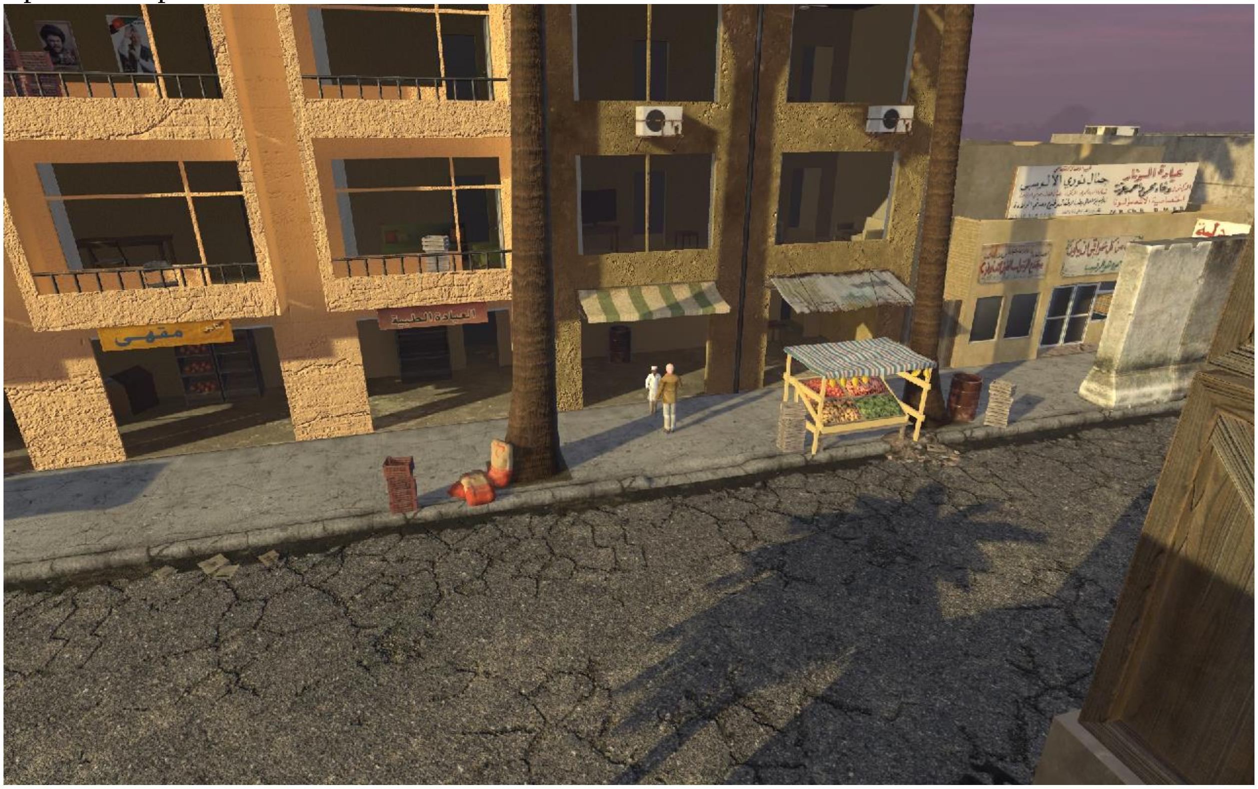

Pictures of characters:

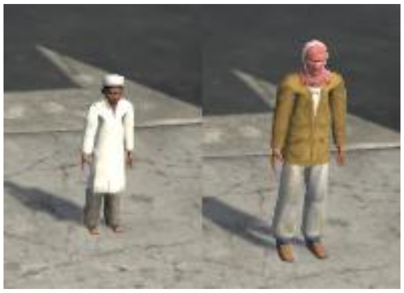

Question 1: Characters A and B were walking before stopping in front of the building
Answer: True

Question 2: A helicopter flew overhead
Answer: False

Question 3: A vehicle drove by and Character B fell to the sidewalk
Answer: True

Question 4: Both characters ran away after the vehicle passed by
Answer: False

Question 5: Only one of the people on the sidewalk fell when the truck passed
Answer: True

Question 6: Characters A and B came from the same direction
Answer: True

Question 7: Only one gunshot is heard
Answer: False

Question 8: Multiple police vehicles passed by after the first vehicle
Answer: True

Question 9: Characters A and B arrive in a car
Answer: False

Question 10: After Character A enters the building a room catches fire
Answer: False

(9) Exposed Rendezvous

Textual prompt: In the episode where two characters meet behind closed blinds, and when the blinds suddenly open, they are exposed and run away

Episode snapshot:

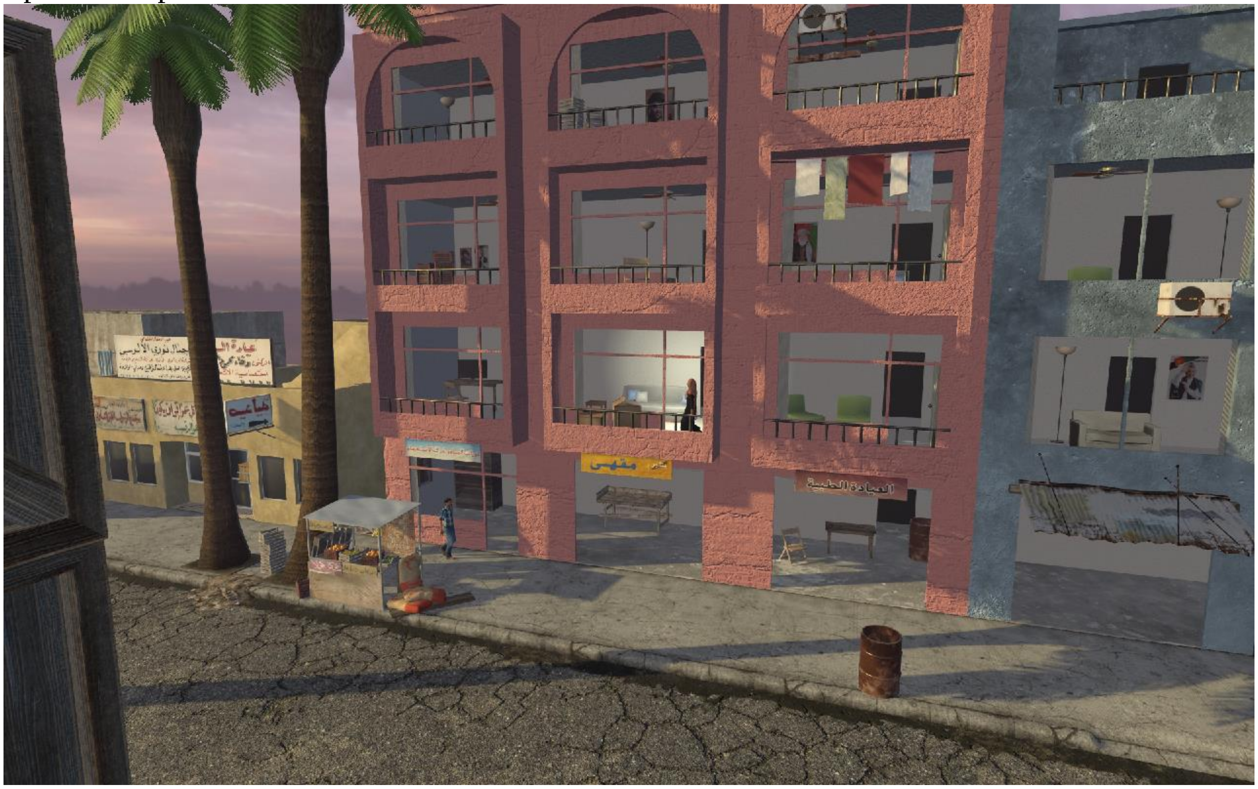

Pictures of characters:

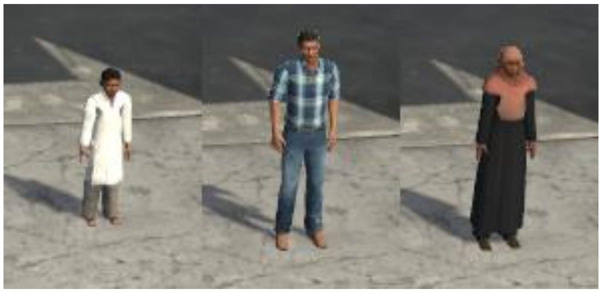

Question 1: Character C is seen entering the building
Answer: False

Question 2: Character B entered the building and is next seen moving up across rooms on every floor
Answer: True

Question 3: The curtains closed when Characters B and C are in a room together
Answer: True

Question 4: Character A is seen moving toward the room just before the curtains open again
Answer: True

Question 5: Character C looked out the window just before the curtain closes
Answer: False

Question 6: When the blinds pull back Character B and C are seen in the room
Answer: True

Question 7: Character C turns the light off when Character B is seen on the street
Answer: False

Question 8: Character A is seen in the room with Characters B and C
Answer: False

Question 9: Characters B and C leave the building and run down the street together
Answer: False

Question 10: When the curtain opens Characters B and C both look out the window
Answer: True

(10) Failed Rescue
Textual prompt: In the episode where a fire breaks out in the building while two characters are talking outside

Episode snapshot:
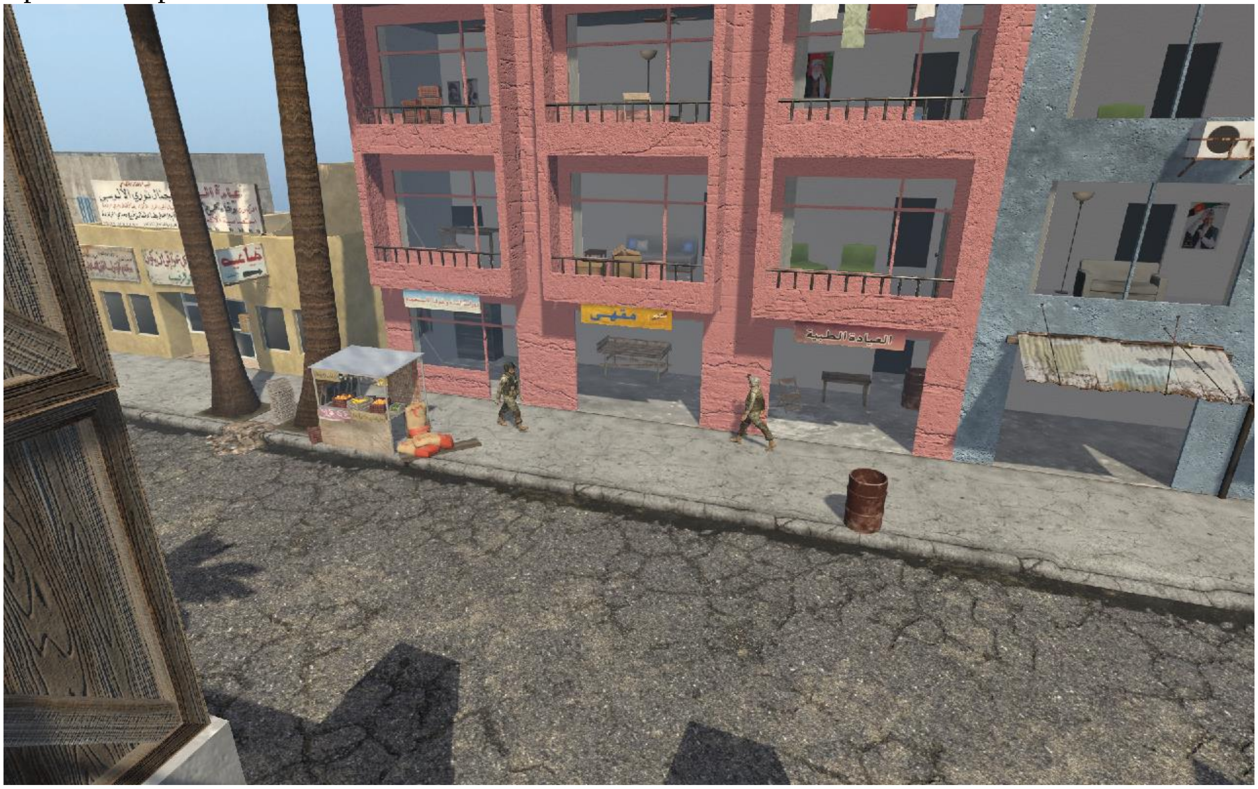

Pictures of characters:
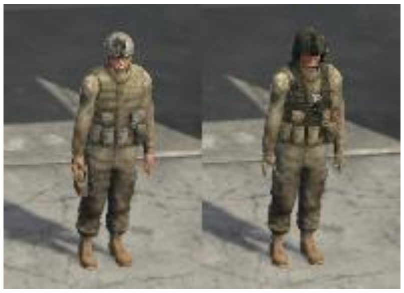

Question 1: The character that enters the building falls while the room he is in is on fire
Answer: True

Question 2: Characters A and B entered the building after fire was seen
Answer: False

Question 3: A woman was seen on inside the building
Answer: True

Question 4: The character in the building fell down as the fire spread
Answer: True

Question 5: The fire spread to many rooms in the building
Answer: True

Question 6: Characters A and B converse in a room inside the building
Answer: False

Question 7: One soldier waited outside while the other soldier entered the building
Answer: True

Question 8: The man who did not enter the building ran away
Answer: False

Question 9: An ambulance drove up to the building
Answer: False

Question 10: A helicopter flew by during the fire
Answer: False

(11) Fire Response
Textual prompt: In the episode where two characters are in or around the building as a fire breaks out in one of the rooms and an ambulance arrives

Episode snapshot:
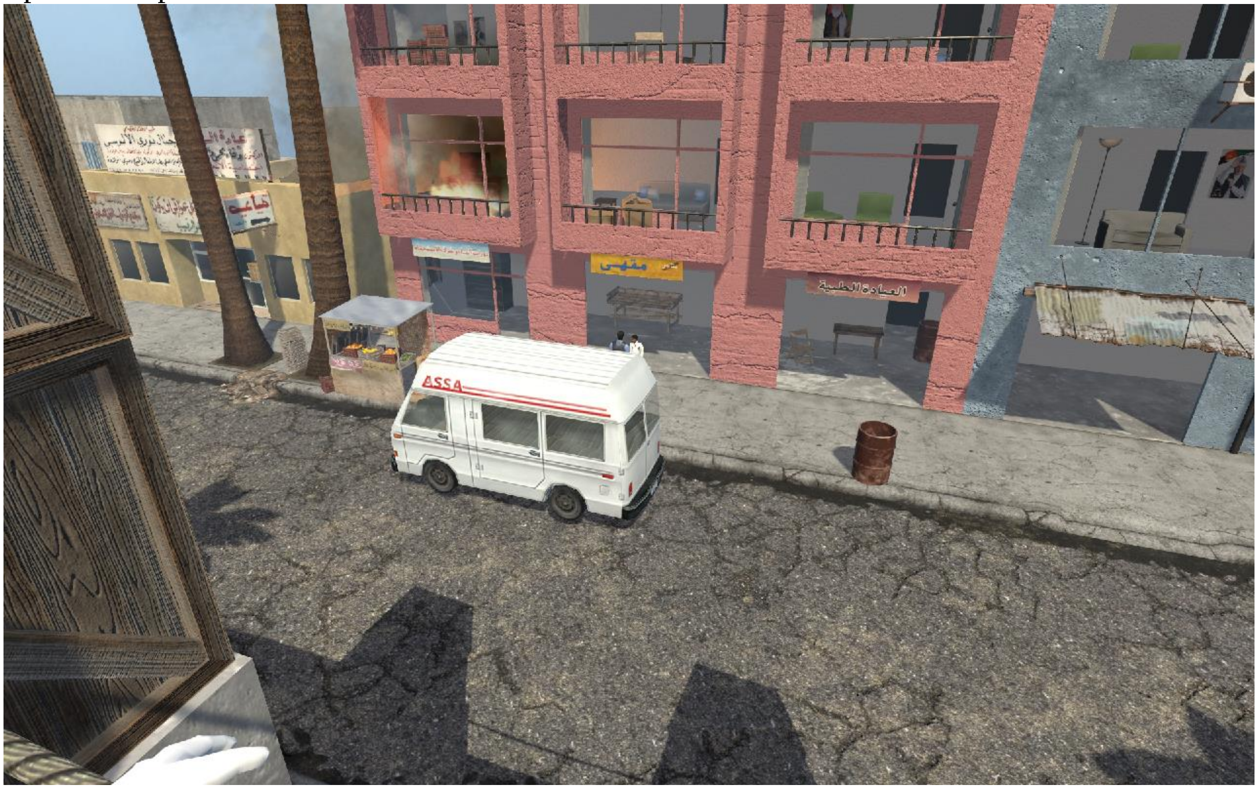

Pictures of characters:
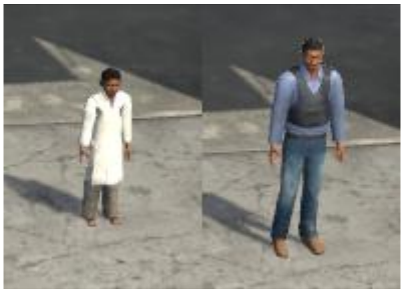

Question 1: Character B is inside the building before Character A
Answer: False

Question 2: Character B looked up at the building before entering
Answer: True

Question 3: Character A was seen in the same room as the fire
Answer: True

Question 4: Character B left the building shortly after the fire started
Answer: False

Question 5: Character B entered the room that was on fire, and left with the child in white
Answer: True

Question 6: Characters A and B talk inside the building
Answer: False

Question 7: An ambulance arrives after Characters A and B exit the building
Answer: True

Question 8: Character A got into the ambulance and it drove away
Answer: False

Question 9: Character A is inside the building before the fire starts
Answer: True

Question 10: Character A runs away after talking to Character B
Answer: False

(12) Handoff

Textual prompt: In the episode where one character is waiting in a room when the other arrives, they meet, they part, and then close the blinds

Episode snapshot:

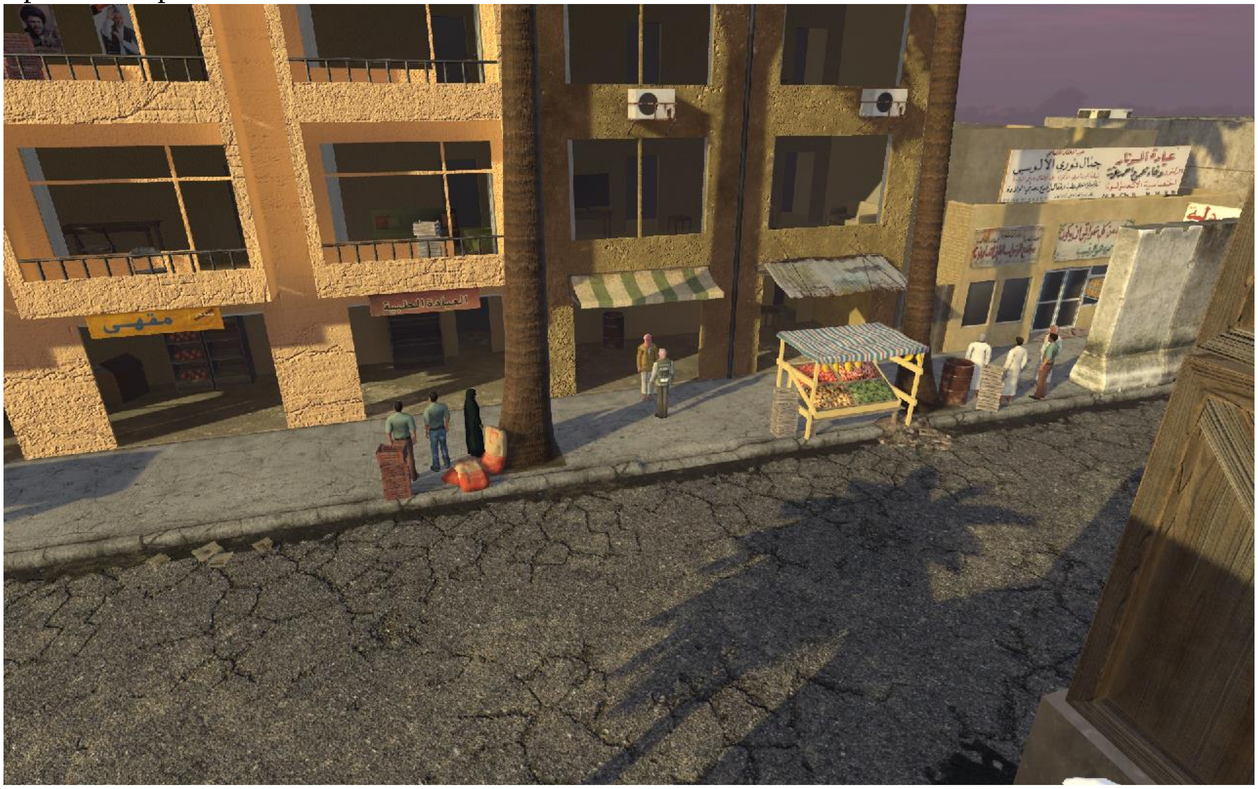

Pictures of characters:

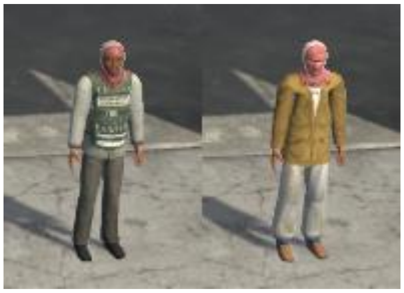

Question 1: Character B is seen pacing and then looking out from the 2nd floor
Answer: True

Question 2: Characters A and B converse inside the building before moving outside
Answer: False

Question 3: Character A leaves in a vehicle that pulls up after conversing with Character B
Answer: True

Question 4: Characters A and B meet only one enters the building
Answer: True

Question 5: When Character B gets back to the room, a police car arrives
Answer: True

Question 6: Character A enters the police car
Answer: False

Question 7: After talking to Character A, Character B walks down the sidewalk
Answer: False

Question 8: When the police car arrives, Character B goes down to meet it
Answer: False

Question 9: When Character B sees Character A on the sidewalk, Character B goes down to meet him
Answer: True

Question 10: The police car chases Character A down the street
Answer: False

(13) Helicopter Scare
Textual prompt: In the episode where a character shoots at a passing helicopter

Episode snapshot:
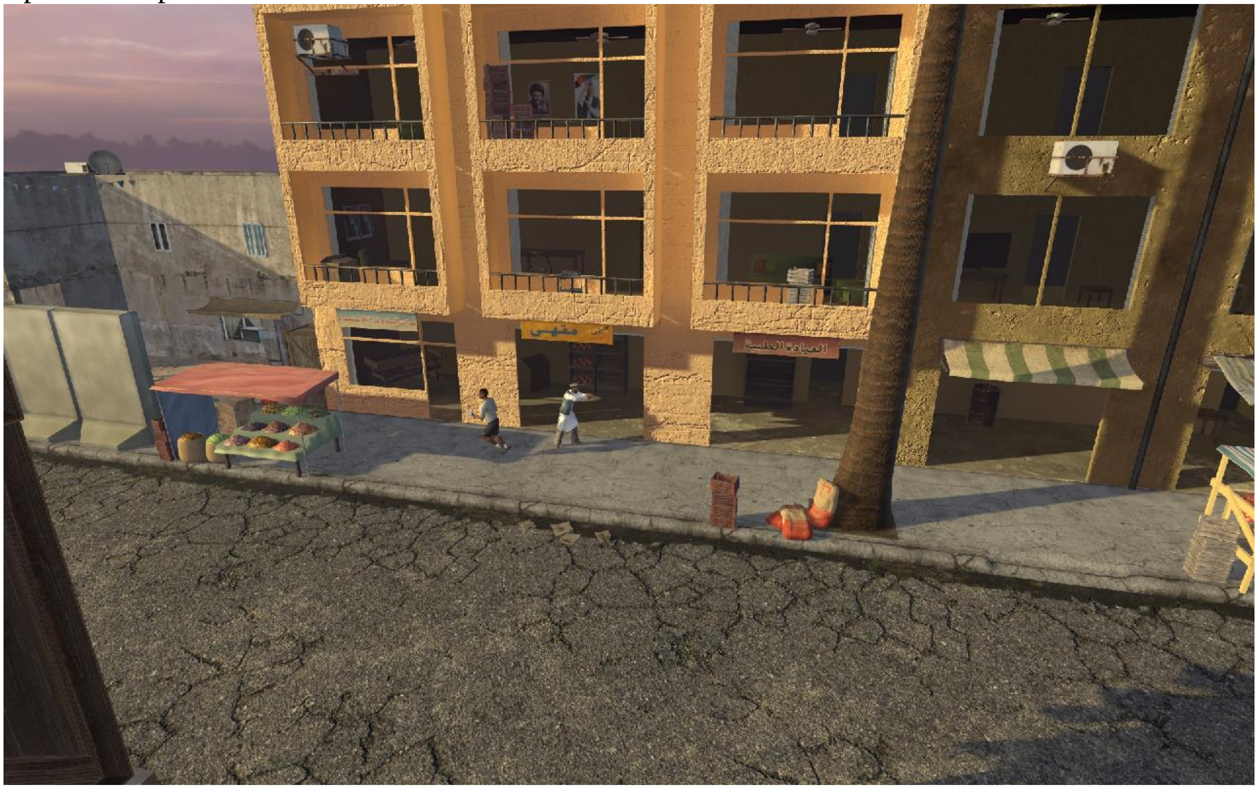

Pictures of characters:
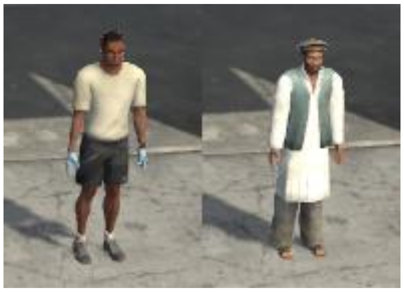

Question 1: When a helicopter is heard Character A runs inside
Answer: True

Question 2: The second time the helicopter is heard Character B runs down the sidewalk
Answer: True

Question 3: Character A ran into the left side entrance of the building
Answer: True

Question 4: An explosion occurs down the street from the building
Answer: False

Question 5: Character A exits the building after the helicopter passes by
Answer: False

Question 6: Character B takes a shooting stance when the helicopter passes overhead the first time
Answer: True

Question 7: Characters A and B ran into the building together
Answer: False

Question 8: Character B ran down the sidewalk to the right
Answer: True

Question 9: When the helicopter passes the second time an explosion occurs
Answer: True

Question 10: An ambulance drives up
Answer: False

(14) Interupted Meeting

Textual prompt: In the episode where two characters are conversing in the building, another arrives and one of the characters leaves with the new arrival

Episode snapshot:

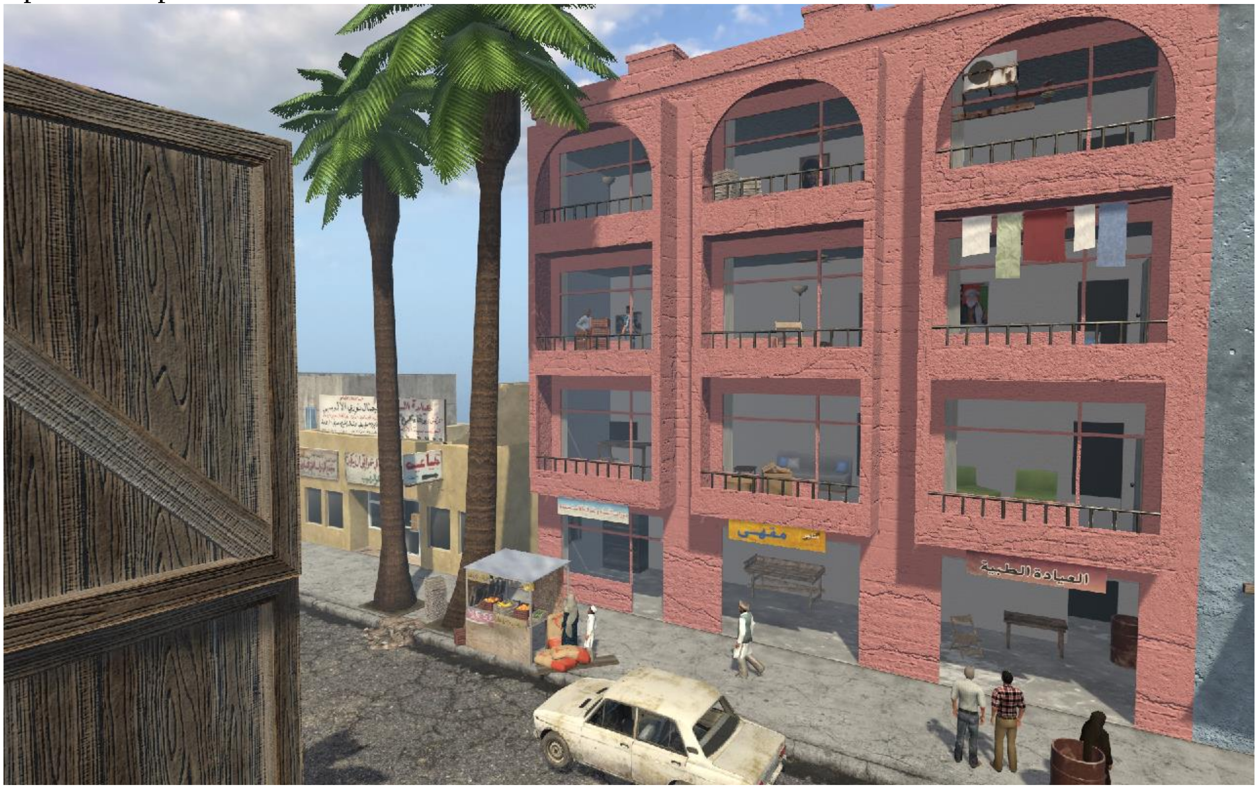

Pictures of characters:

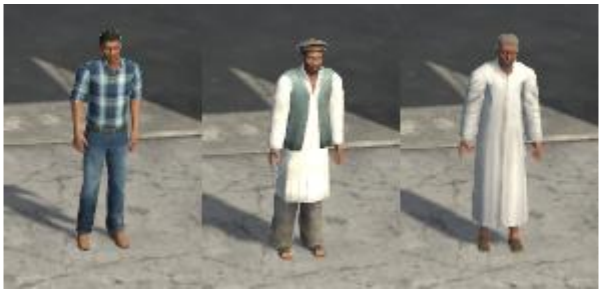

Question 1: Characters A and B are talking together in the room before Character C arrives
Answer: False

Question 2: Character B waits on the street and waits for Character A to come down
Answer: False

Question 3: A bomb explodes on the street
Answer: False

Question 4: Character B leaves the room with Character A
Answer: True

Question 5: Character C leaves and runs down the street
Answer: False

Question 6: After the two men leave in the car, the blinds close in the room
Answer: True

Question 7: Character C appears in the next room after the other two men leave
Answer: False

Question 8: Character C left the building
Answer: False

Question 9: Characters A and B converse in multiple rooms in the building
Answer: False

Question 10: Character C remains in the same room after Characters A and B depart
Answer: True

(15) Missed The Bus
Textual prompt: In the episode where two characters get into a vehicle and another runs after it down the sidewalk

Episode snapshot:

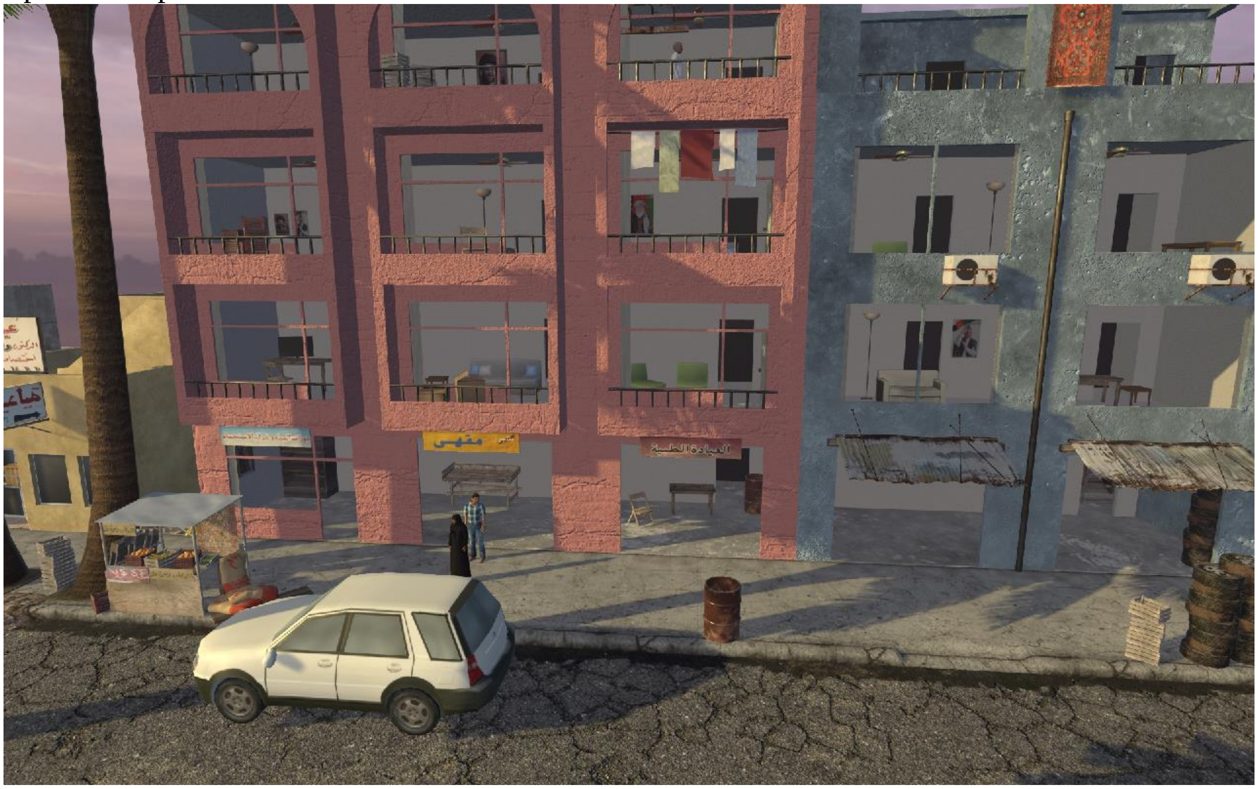

Pictures of characters:

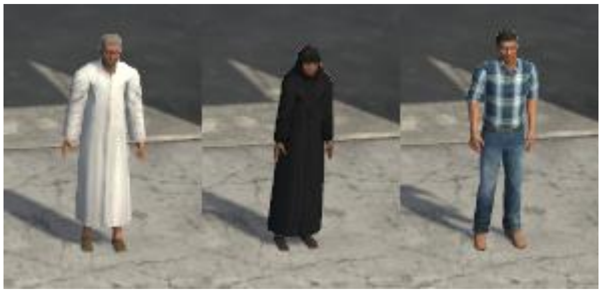

Question 1: Character B was never seen in an upstairs floor prior to the arrival of the car
Answer: True

Question 2: The car arrived after people were already waiting on the sidewalk
Answer: True

Question 3: After running down the stairs Character A gets into the vehicle
Answer: False

Question 4: Character A went back into the building
Answer: False

Question 5: Character A entered the car first
Answer: False

Question 6: Characters B and C exited the building from different exits
Answer: False

Question 7: Characters B and C argued with the driver and the car drove away leaving them on the sidewalk
Answer: False

Question 8: Characters B and C were waiting on the sidewalk when the car drove up
Answer: True

Question 9: There was no indication that Characters B and C knew each other
Answer: True

Question 10: Character A ran down the sidewalk after the car
Answer: True

(16) Missile Strike

Textual prompt: In the episode where two characters are in different rooms of the building when an explosion occurs

Episode snapshot:

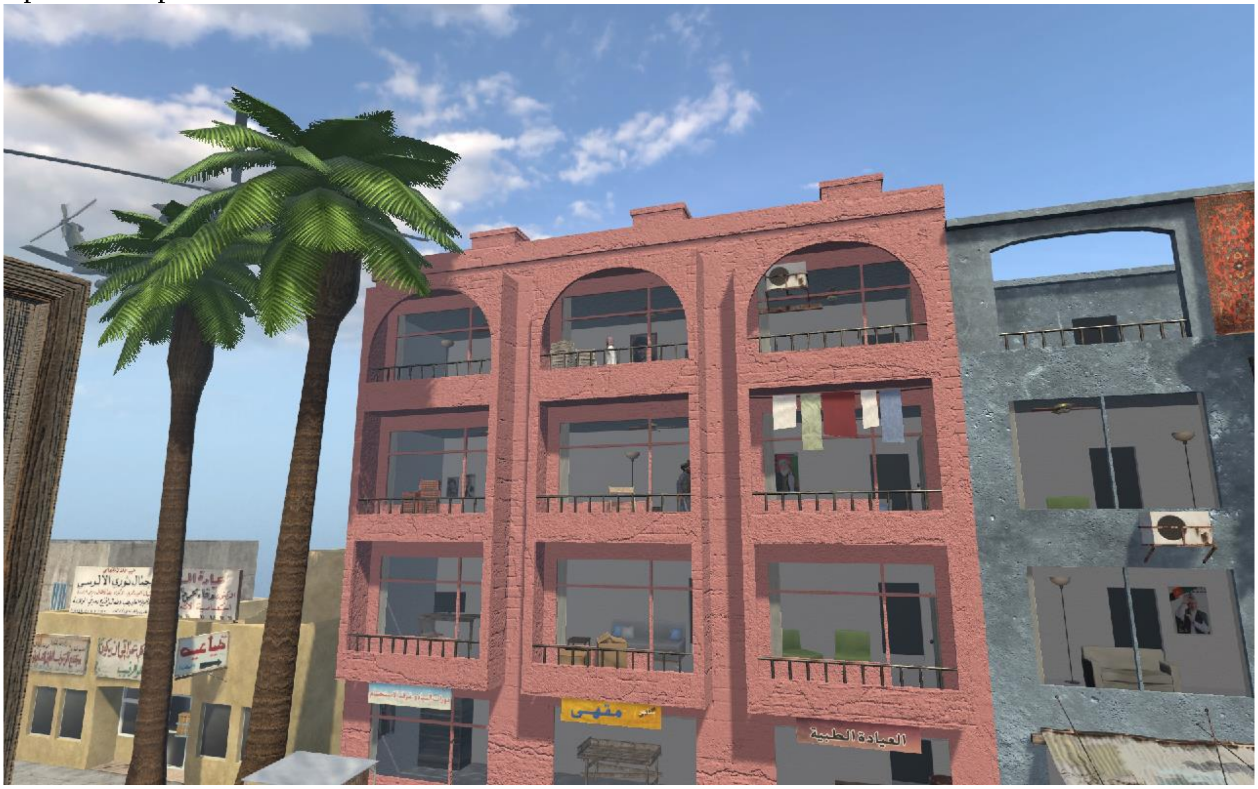

Pictures of characters:

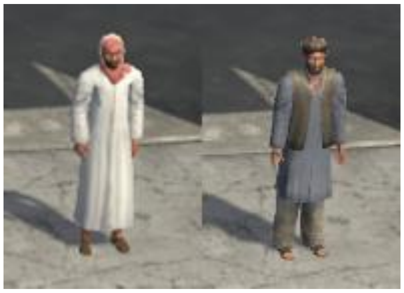

Question 1: Character A enters the building before anyone else was seen in it
Answer: True

Question 2: Character A peeked out of a window before the explosion in that room
Answer: True

Question 3: Character B is visible in the room with the explosion when it occurs
Answer: False

Question 4: Character B ran out of the building right after the explosion
Answer: True

Question 5: An ambulance drives up after explosion
Answer: False

Question 6: A helicopter is heard right before the explosion occurs
Answer: True

Question 7: Character B enters the building after the explosion
Answer: False

Question 8: Character A falls down while running on the street
Answer: False

Question 9: Character A is not seen after explosion
Answer: True

Question 10: Character A fires multiple gunshots before helicopter is heard
Answer: False

(17) No Escape
Textual prompt: In the episode where two characters are in the building when a fire starts and spreads throughout the structure

Episode snapshot:

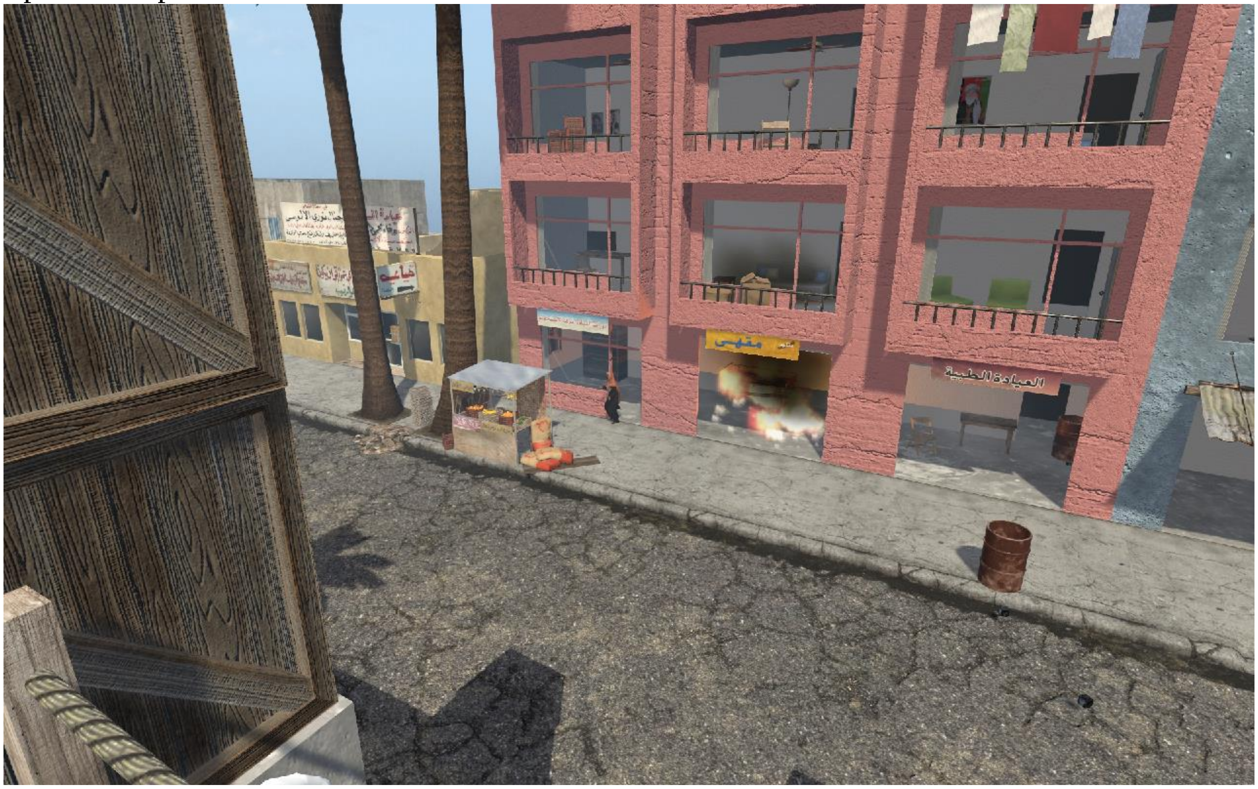

Pictures of characters:

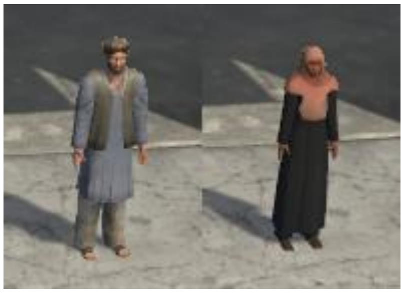

Question 1: An explosion occurs on the bottom floor at the start of the episode
Answer: True

Question 2: Character A runs out of the building after the explosion
Answer: False

Question 3: As the fire spreads, an ambulance arrives
Answer: False

Question 4: The fire spreads across the bottom floor before spreading upward
Answer: True

Question 5: Fire eventually spread to all floors of the building
Answer: True

Question 6: Character B is first seen the 3rd floor, and later is seen on the top floor
Answer: False

Question 7: Character A falls down in a room that is on fire
Answer: True

Question 8: Character B reenters the building after leaving it
Answer: False

Question 9: Characters A and B meet in the building before the fire
Answer: False

Question 10: Character A is seen on the top floor
Answer: True

(18) Pick Pocket

Textual prompt: In the episode where one character takes something from the other while their back is turned, and then a chase ensues down the sidewalk

Episode snapshot:

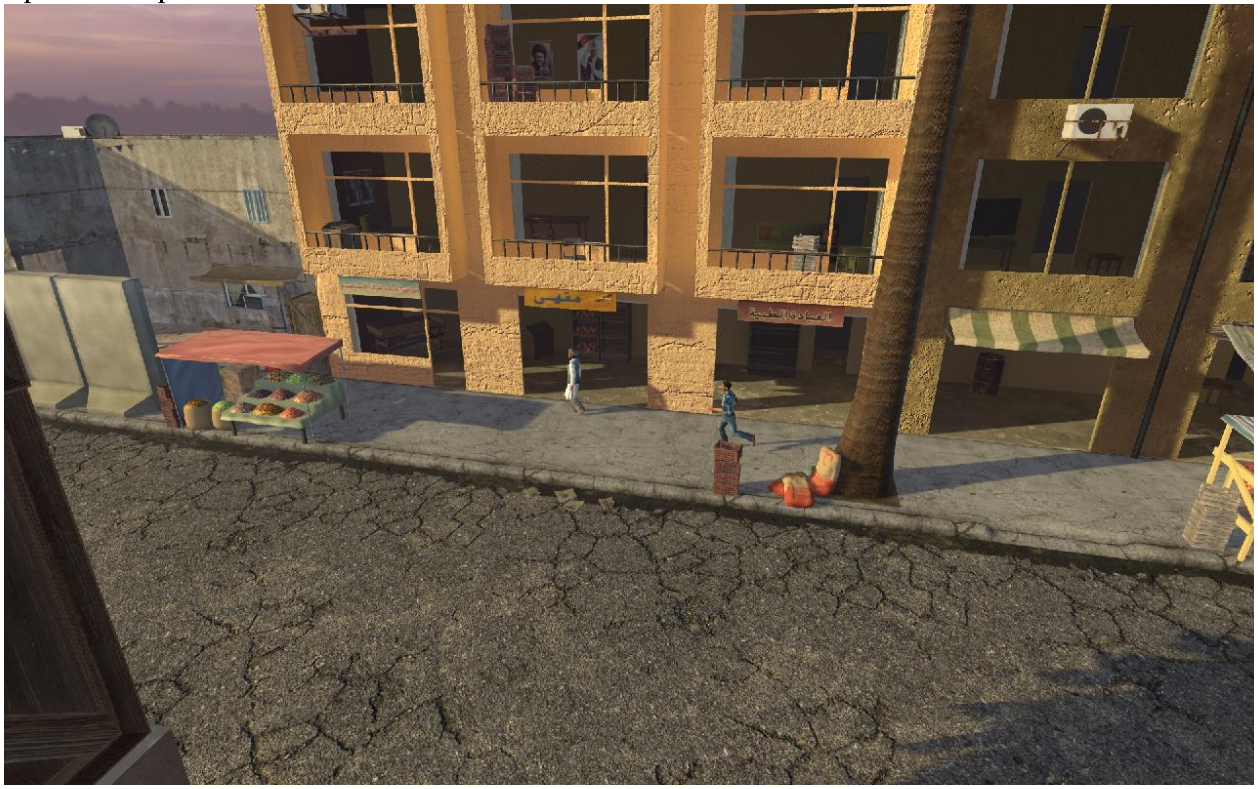

Pictures of characters:

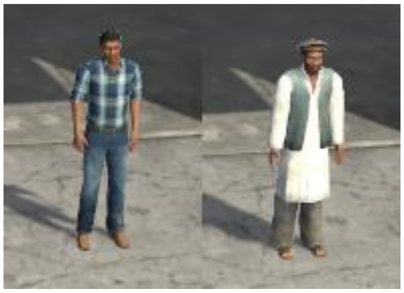

Question 1: Character B faces the building after leaving it
Answer: True

Question 2: Character A got out of a car and stood close to Character B
Answer: False

Question 3: Characters A and B continue running in the same direction
Answer: True

Question 4: Character B entered the building
Answer: False

Question 5: A police car arrived
Answer: False

Question 6: Character B shoots at Character A
Answer: False

Question 7: Character B is not seen catching Character A
Answer: True

Question 8: Character A followed Character B down the sidewalk
Answer: True

Question 9: Character A jumped in a car and drove off
Answer: False

Question 10: Character B walks by Character A before Character A takes something from Character B
Answer: True

(19) Repair Man

Textual prompt: In the episode where smoke is coming from a car by the sidewalk until a character fixes it

Episode snapshot:

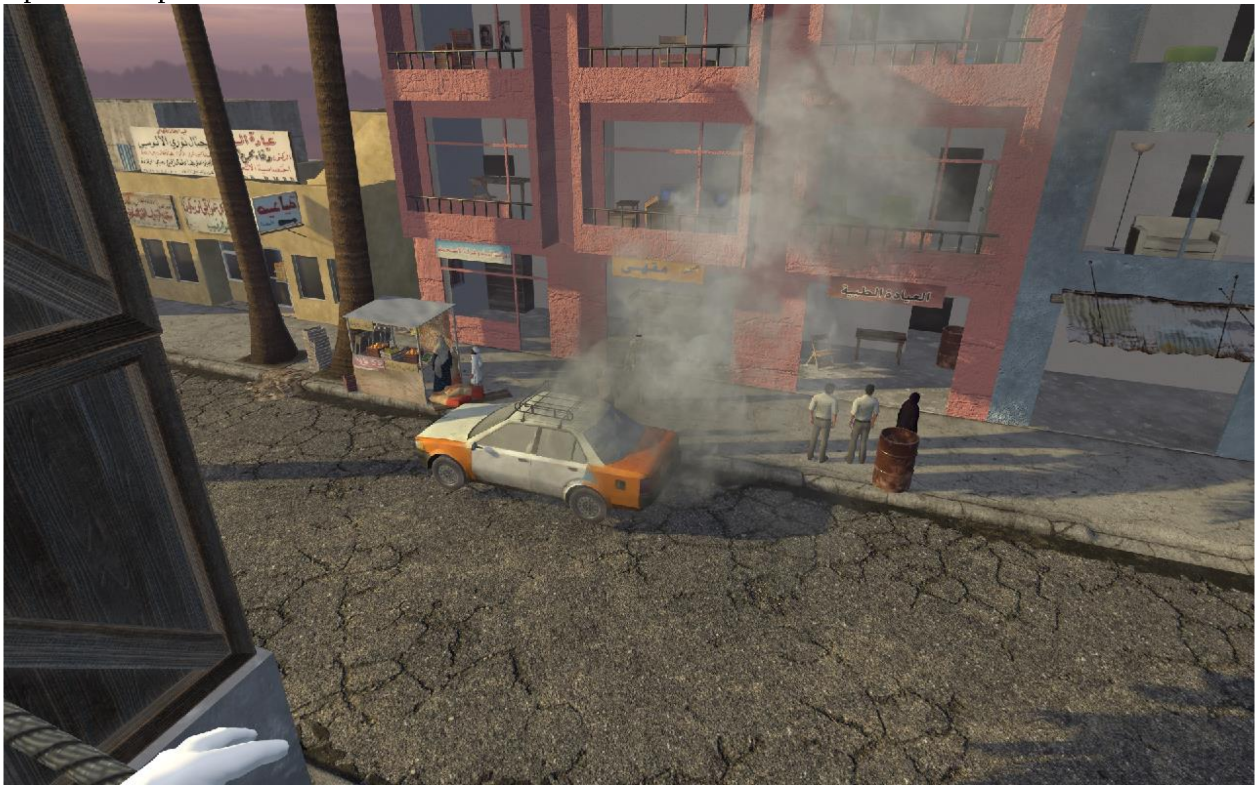

Pictures of characters:

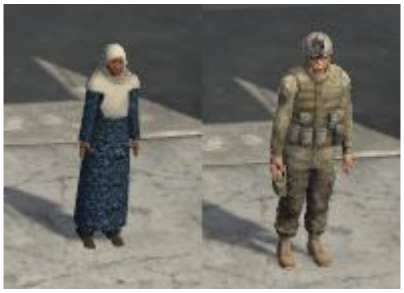

Question 1: The car is already parked by the sidewalk at the start of the episode
Answer: False

Question 2: Character B approaches the car from the left
Answer: True

Question 3: Characters A and B both enter the car
Answer: False

Question 4: Character A goes into the building while Character B is interacting with the car
Answer: False

Question 5: Character A drives off in the car after it stops smoking
Answer: True

Question 6: Character A gets out the car after it starts smoking
Answer: True

Question 7: Character B goes into the building with Character A
Answer: False

Question 8: Character B joins Character A on the sidewalk while the car is smoking
Answer: True

Question 9: Character B steps toward the car and appears to take something out of his pocket, and then the smoke disappears
Answer: True

Question 10: Characters A and B converse again after the car stops smoking
Answer: True

(20) Shopping
Textual prompt: In the episode where several characters enter the shop, some speak to the owner, and some leave

Episode snapshot:
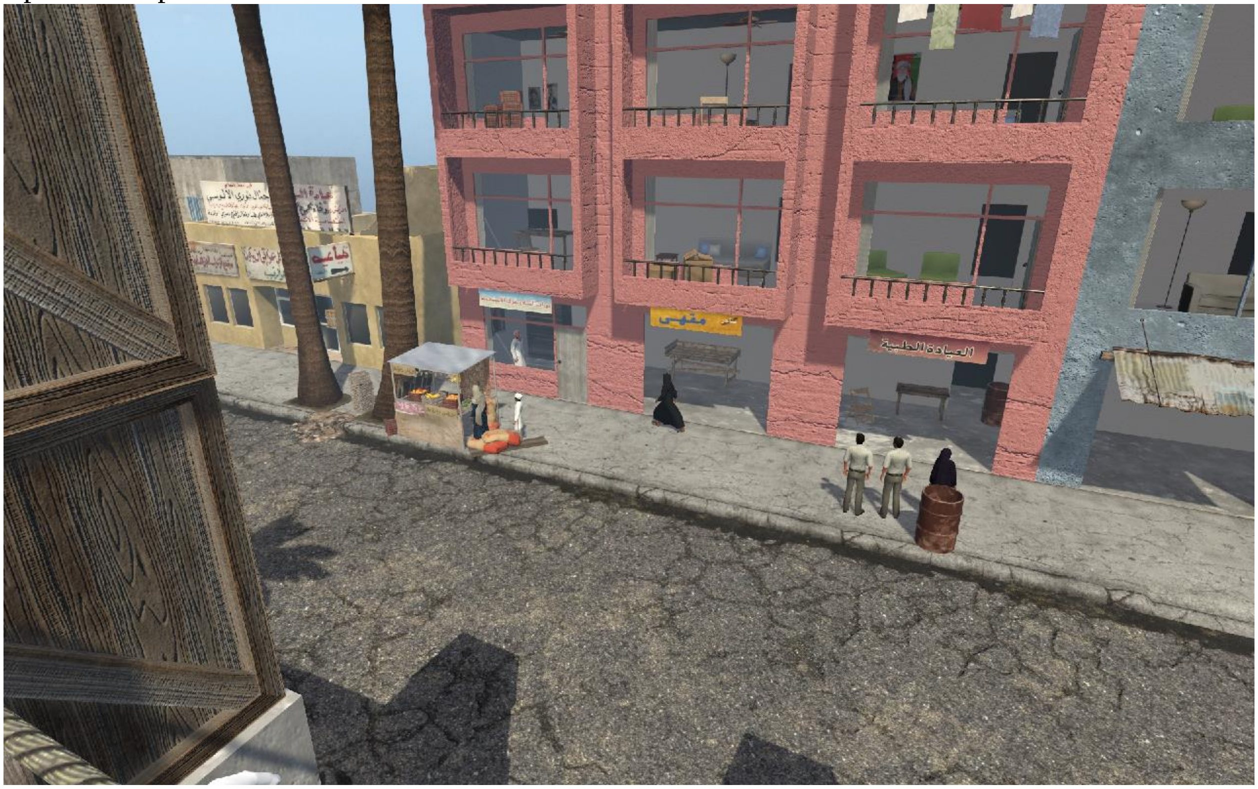

Pictures of characters:
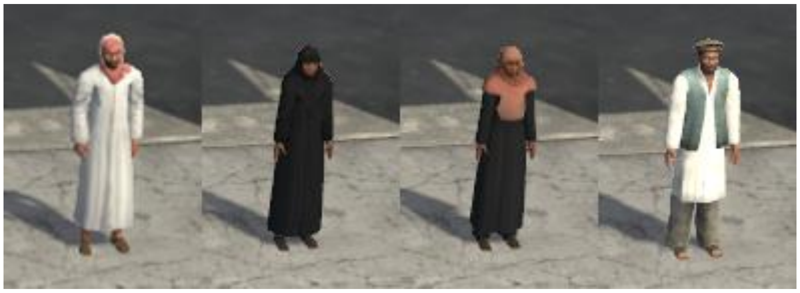

Question 1: Character B got out of a truck and went into a shop
Answer: False

Question 2: Character B paused to look in a window before entering the shop
Answer: True

Question 3: Character A shot Character B in the shop
Answer: False

Question 4: Character C entered the room Character B had left
Answer: True

Question 5: Character D left the building and drove away in a car
Answer: False

Question 6: Character C got out of a car and entered the shop
Answer: True

Question 7: Characters C and D got out of the same car
Answer: False

Question 8: Character D fell down on the sidewalk outside
Answer: False

Question 9: Character A remains in the shop as Character C enters
Answer: True

Question 10: Character B is seen speaking with the owner
Answer: True

(21) Sniper

Textual prompt: In the episode where two characters are conversing on the sidewalk while another is on the roof and gunshots are heard

Episode snapshot:

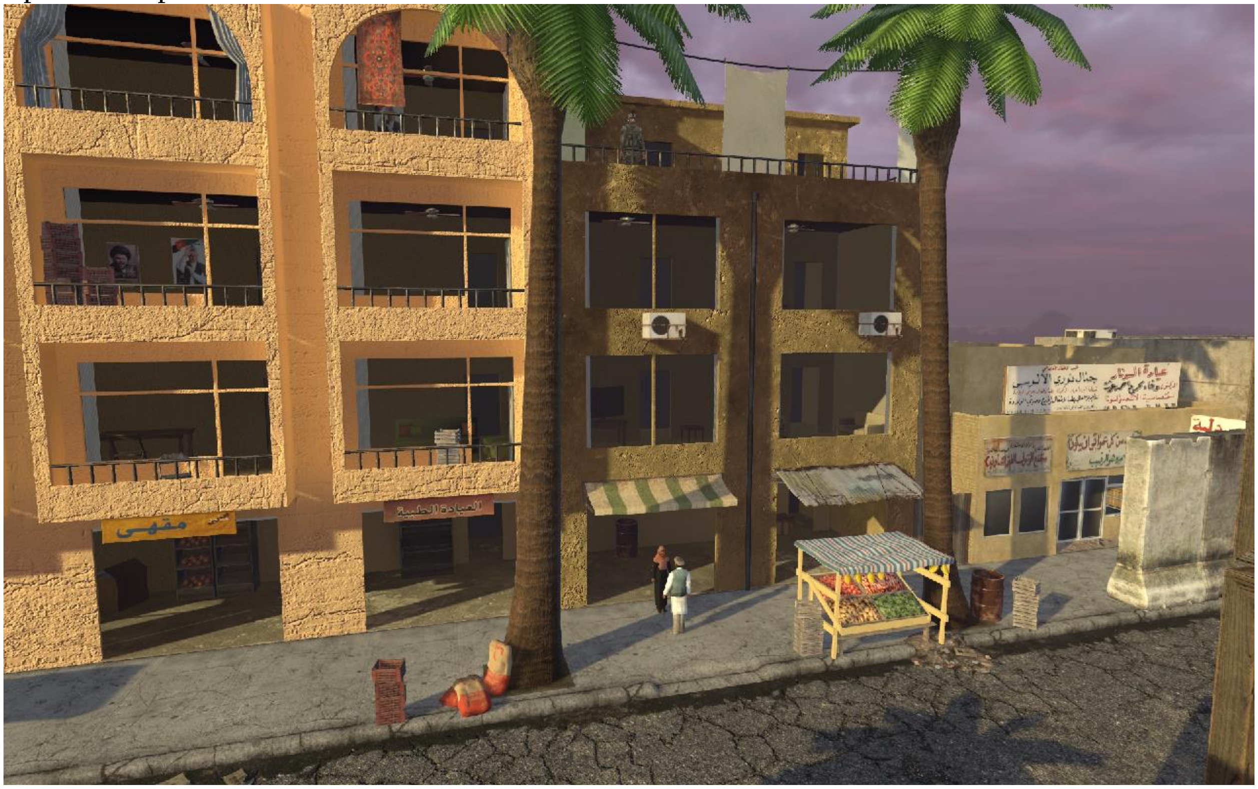

Pictures of characters:

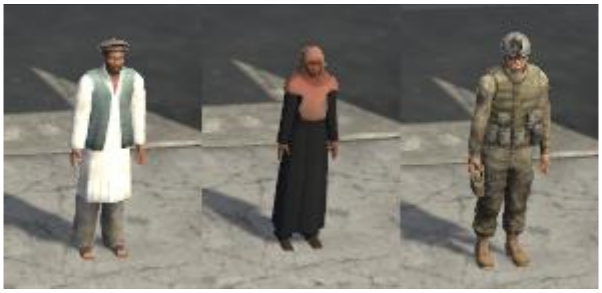

Question 1: Character B ran away alone after gunshots were heard
Answer: True

Question 2: Character B ran into the building after shots were heard
Answer: False

Question 3: Characters A and B ran away when Character C appeared on roof
Answer: False

Question 4: Character C appeared on the roof, and then disappeared and automatic gunfire was heard
Answer: True

Question 5: After the shots were heard, Character C reappeared on the roof
Answer: True

Question 6: Character A got shot and Character B ran down the sidewalk to the left
Answer: True

Question 7: Character B left more quickly than they arrived
Answer: True

Question 8: An ambulance arrived
Answer: False

Question 9: Characters A and B came out of the building
Answer: False

Question 10: Character B got in a car and drove away
Answer: False

(22) Street Hawker

Textual prompt: In the episode where one character on the sidewalk stops the other to talk, follows them, and then convinces them to enter the building

Episode snapshot:

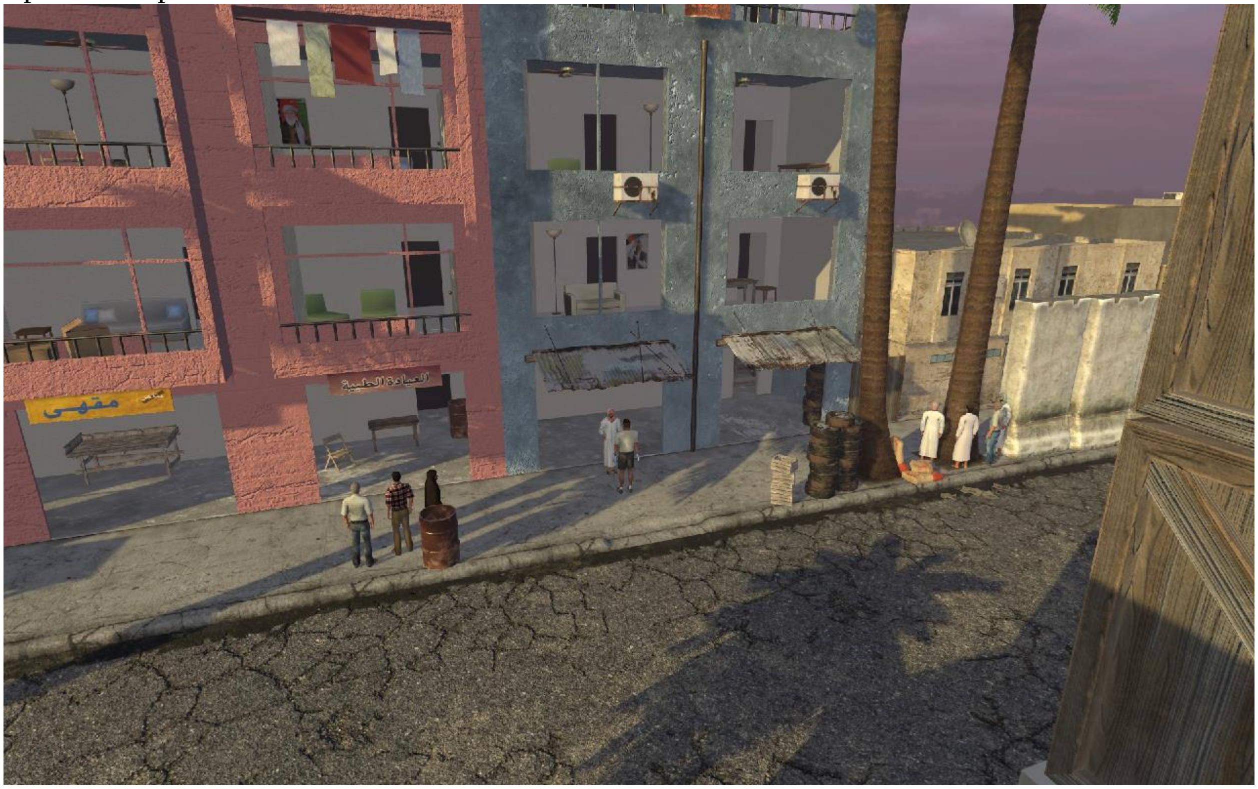

Pictures of characters:

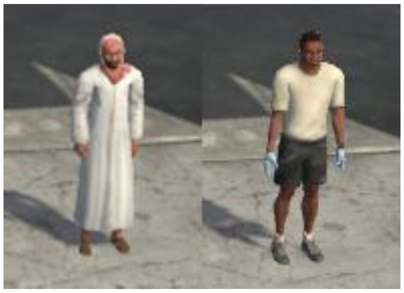

Question 1: Two people met on the sidewalk at right side of building, then walked to left side of building
Answer: True

Question 2: After stopping at left side of building, Character A got in a car and drove away
Answer: False

Question 3: Characters A and B conversed a second time after Character B walks further down the street
Answer: True

Question 4: Gunshots are heard before the two enter the building
Answer: False

Question 5: The people talking on the sidewalk entered the building on the right side, and the garage door closed behind them
Answer: True

Question 6: Smoke is seen coming from the room the characters entered
Answer: True

Question 7: An emergency vehicle arrives after smoke is seen
Answer: False

Question 8: Characters A and B enter the building together
Answer: True

Question 9: Characters A and B converse in a room on an upper floor of the building
Answer: False

Question 10: Character A enters the building while Character B remains outside
Answer: False

(23) Suicide Bomb
Textual prompt: In the episode where one character enters the building, meets with the other character in a room, then there is an explosion in that room

Episode snapshot:

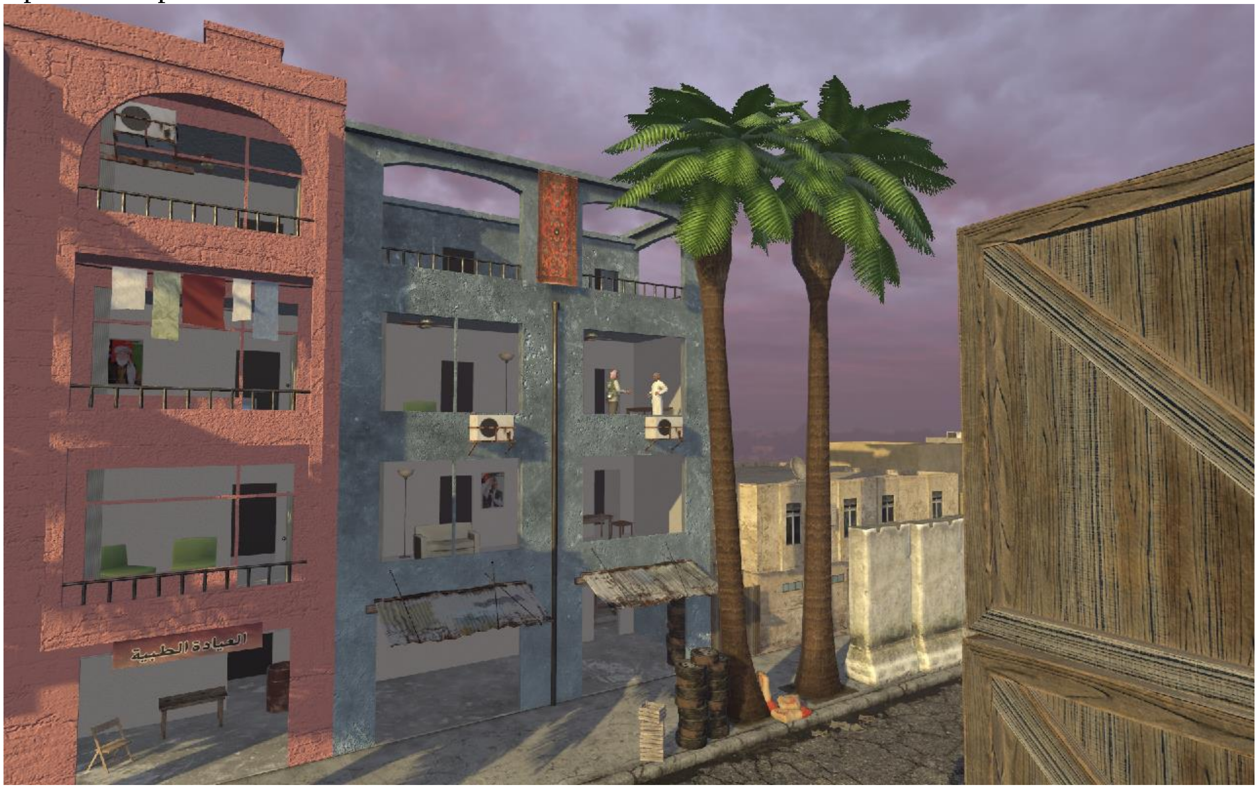

Pictures of characters:

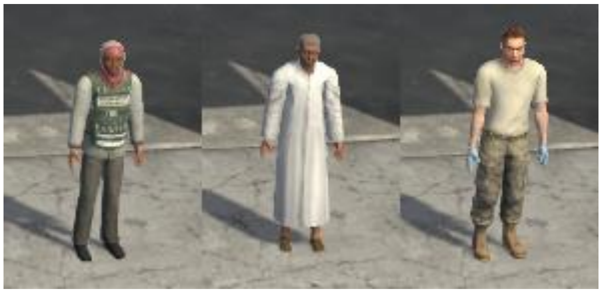

Question 1: Character A entered the left entrance of the building
Answer: False

Question 2: Character B was already in the building when Character A entered
Answer: True

Question 3: An ambulance arrives after explosion
Answer: True

Question 4: Character B tries to run before the explosion occurs
Answer: False

Question 5: Fire starts in the room after the explosion
Answer: True

Question 6: Character A runs from building after the explosion
Answer: False

Question 7: Character C arrives in a vehicle
Answer: True

Question 8: Character B leaves in a car
Answer: False

Question 9: Characters A and B are not seen after the explosion
Answer: True

Question 10: Characters A and B converse outside after the explosion
Answer: False

(24) The Dare
Textual prompt: In the episode where two characters start a fire in the building

Episode snapshot:
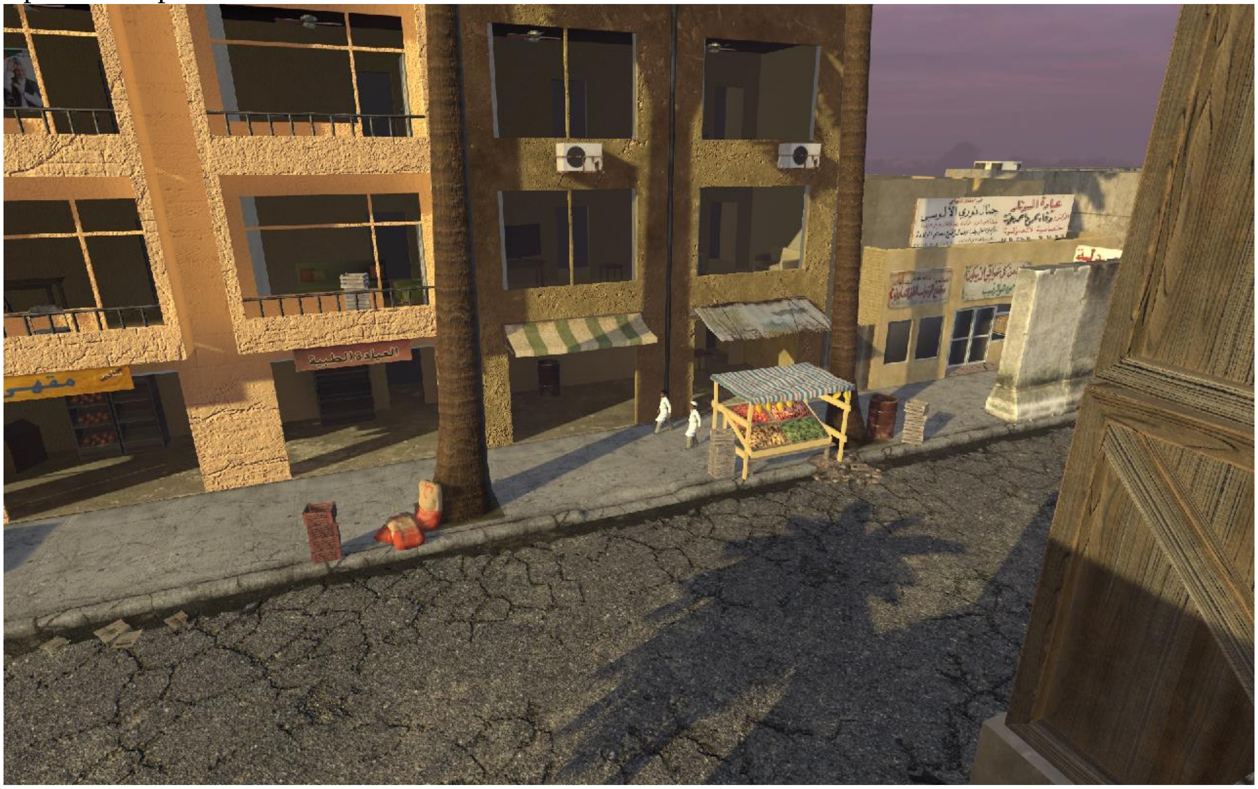

Pictures of characters:
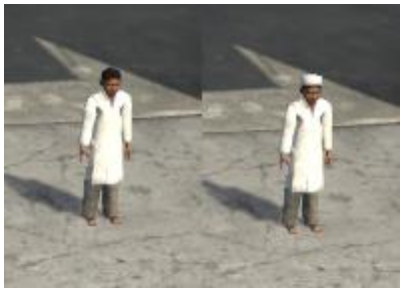

Question 1: Characters A and B stop to talk outside building
Answer: True

Question 2: Characters A and B enter the building together
Answer: False

Question 3: After Character B enters the building a room catches fire
Answer: True

Question 4: A police vehicle drives in the direction Characters A and B ran away
Answer: False

Question 5: Character B leaves the building after the fire starts
Answer: True

Question 6: Character B passes through a room while it is on fire
Answer: False

Question 7: Character A waits outside while Character B enters the building
Answer: True

Question 8: Character B gets in a car and drives off
Answer: False

Question 9: The fire spreads to many rooms
Answer: False

Question 10: Characters A and B leave headed the same direction
Answer: True

(25) The Meeting
Textual prompt: In the episode where two characters meet in a room and a third character is seen on the sidewalk

Episode snapshot:

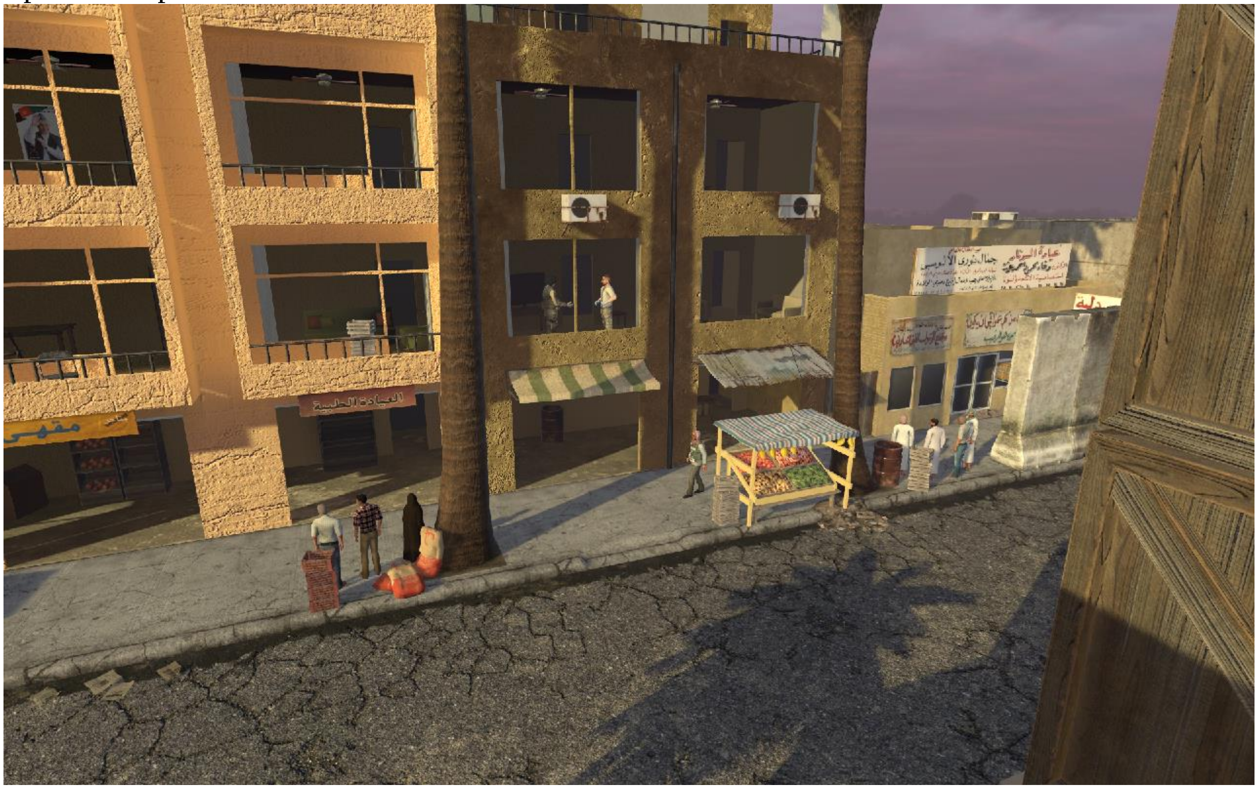

Pictures of characters:

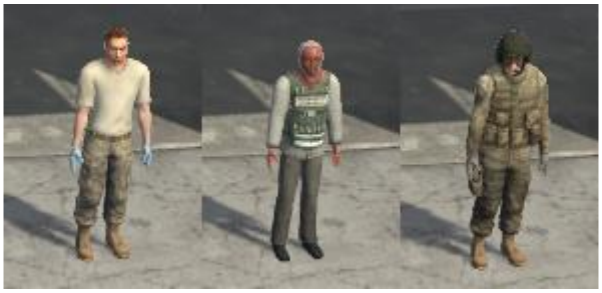

Question 1: Character A enters the building before Character C
Answer: True

Question 2: Character B came out of the building
Answer: False

Question 3: Characters A and C leave the building and go the same direction as Character B
Answer: True

Question 4: Character B enters the building through the same entrance as Characters A and C
Answer: False

Question 5: Character A watches out the window as Character C approaches
Answer: True

Question 6: Characters A and C close the curtains before they leave the room
Answer: False

Question 7: Character B got out of a car
Answer: False

Question 8: A police car drove by
Answer: False

Question 9: Characters A and C both entered the same building entrance
Answer: True

Question 10: Character B ran away when he saw the other two in the building
Answer: True

(26) The Serenade
Textual prompt: In the episode where one character on the sidewalk sings to the other character in the building

Episode snapshot:
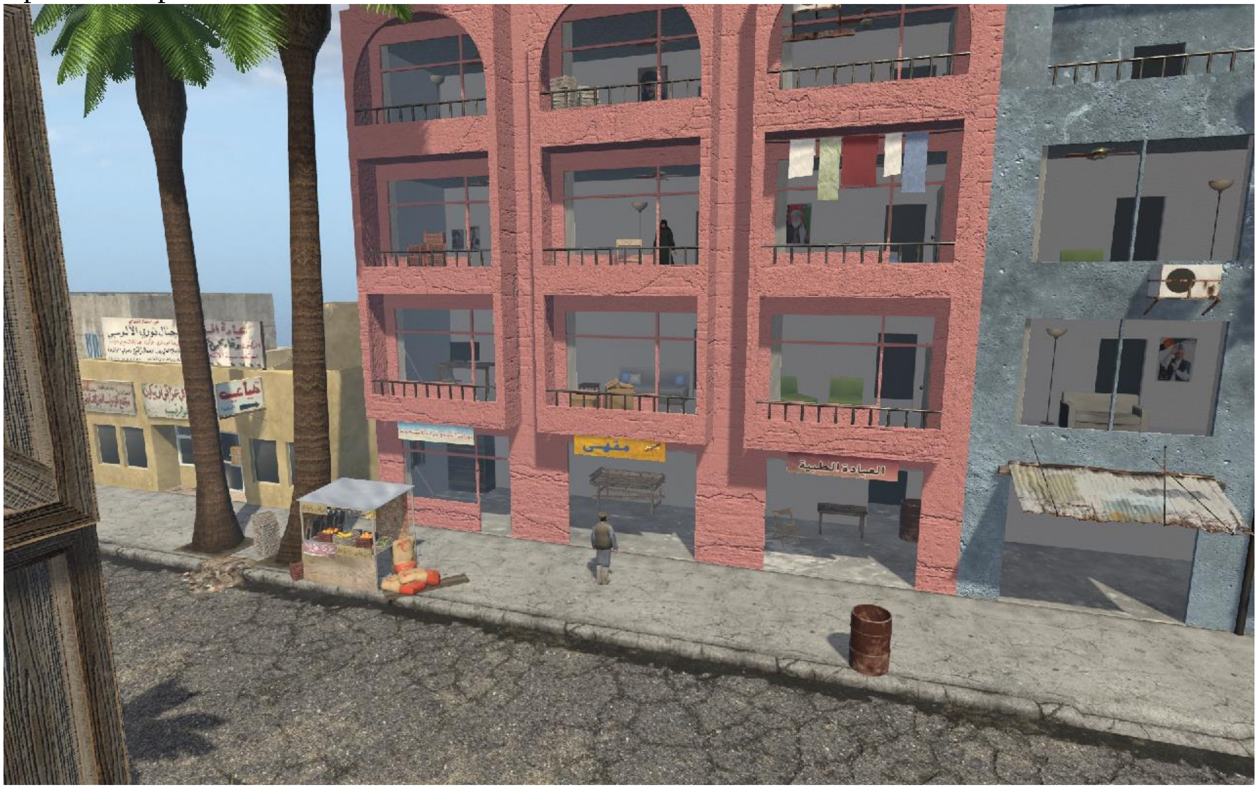

Pictures of characters:
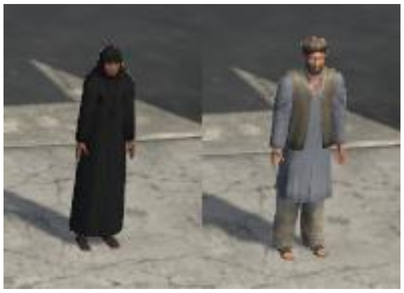

Question 1: Character B came walking down the sidewalk from the right to the front of the building
Answer: True

Question 2: Character A waited for Character B to finish singing before coming down
Answer: True

Question 3: Character A in the building drew the blinds after listening
Answer: False

Question 4: Character B started singing before Character A came to the window
Answer: True

Question 5: Character A came down and joined Character B, and they went down the sidewalk together
Answer: True

Question 6: Character B arrived in a car
Answer: False

Question 7: Character A came down and joined Character B, and they left in a car
Answer: False

Question 8: Character B entered the building and met Character A in her room
Answer: False

Question 9: There was no vehicle of any kind in this vignette
Answer: True

Question 10: After speaking on the side walk the two characters walked in opposite directions
Answer: False

(27) Timely Exit
Textual prompt: In the episode where one character speaks with the other character in the building, and they leave before an explosion occurs in the room they were just in

Episode snapshot:
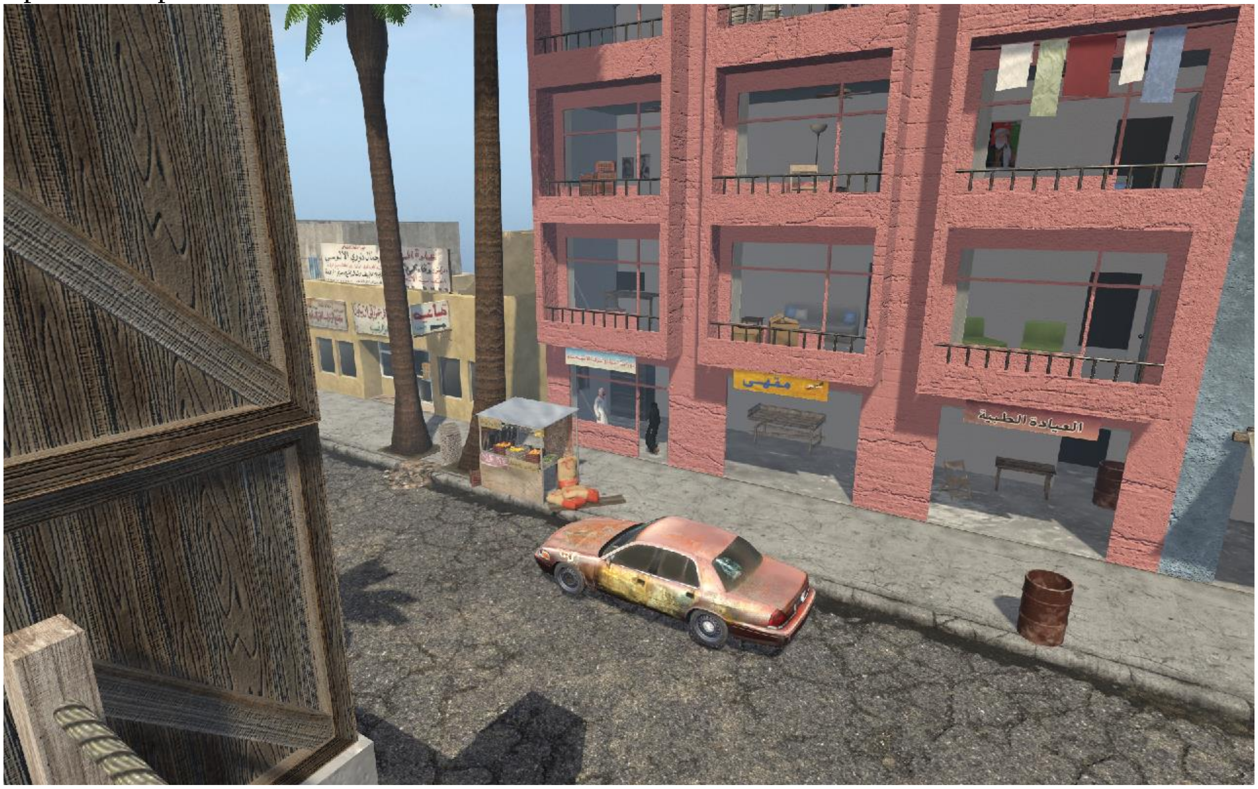

Pictures of characters:
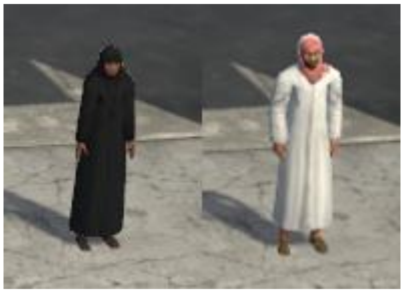

Question 1: Character A arrived in a car
Answer: True

Question 2: Character A entered the building to converse with Character B
Answer: True

Question 3: The door of the store was open while the two people talked inside
Answer: False

Question 4: A second vehicle arrives before first leaves
Answer: False

Question 5: There was a second explosion after the first
Answer: False

Question 6: A helicopter flew over just before the explosion
Answer: True

Question 7: An ambulance arrives after the explosion
Answer: False

Question 8: Character A falls down in the street after explosion
Answer: False

Question 9: Character B runs down the street after explosion
Answer: False

Question 10: Characters A and B entered a car and drove away
Answer: True

(28) Waiting Gunman
Textual prompt: In the episode where one character shoots the other as they leave the building

Episode snapshot:
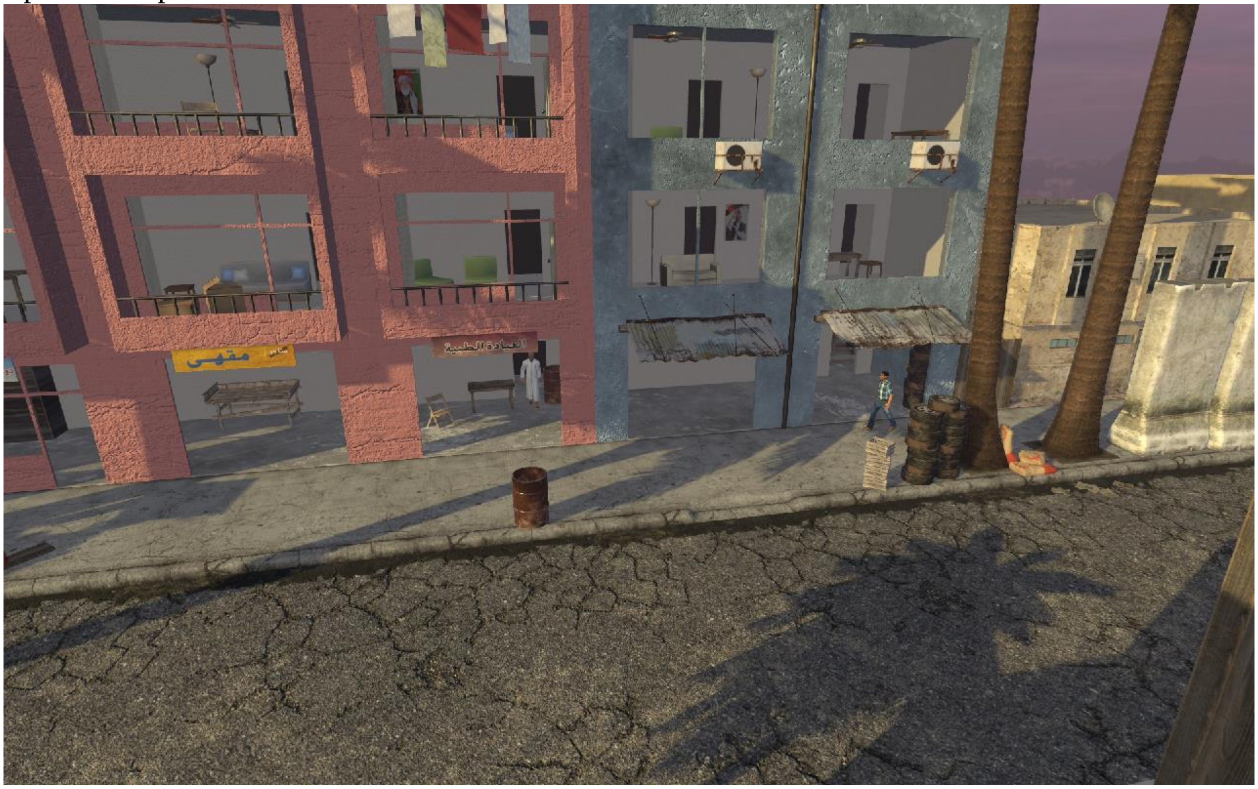

Pictures of characters:
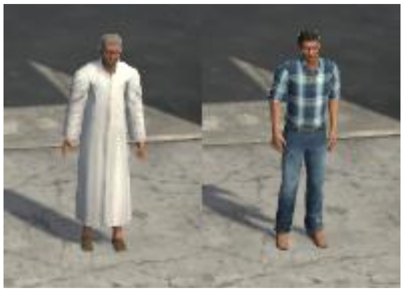

The 10 questions:
Question 1: Character B fires many shots
Answer: True

Question 2: Character B shoots at Character A
Answer: True

Question 3: Character A falls while running away
Answer: True

Question 4: Character B runs past Character A
Answer: True

Question 5: A car pulls up and Character B gets in
Answer: False

Question 6: Character A starts to run when shooting begins
Answer: True

Question 7: Characters A and B are facing each other when shots are fired
Answer: False

Question 8: Character A looks out of a window before leaving the building
Answer: False

Question 9: An emergency vehicle arrives
Answer: False

Question 10: Character B turns to run away from the place where Character A has fallen
Answer: False
